# RNA targeting therapy for a prenatally enriched potassium channel associated with severe childhood epilepsy and premature death

**DOI:** 10.1038/s41467-026-72334-7

**Published:** 2026-04-29

**Authors:** Sean R. Golinski, Karla Soriano, Alex C. Briegel, Madeline C. Burke, Sheng Tang, Gemma L. Carvill, Emma Sherrill, Claudia Lentucci, Timothy W. Yu, Tojo Nakayama, Ruilong Hu, Richard S. Smith

**Affiliations:** 1 Northwestern University, Feinberg School of Medicine, Chicago, IL USA; 2 Division of Genetics and Genomics, Boston Children’s Hospital, Boston, MA USA

**Keywords:** Target validation, Disease model

## Abstract

Dysfunction of the sodium-activated potassium channel K_Na_1.1 (encoded by *KCNT1)* is associated with a severe neurodevelopmental condition characterized by frequent seizures (up to hundreds per day), treatment resistance, and increased mortality during childhood. Yet, recent progress with an RNA therapy targeting KCNT1 offers clinical promise^1^. We characterize the early developmental onset of K_Na_1.1 channels in prenatal and neonatal brain tissue, establishing a timeline for pathophysiology and a window for therapeutic intervention. Using patch-clamp electrophysiology, we observe functional prenatal K_Na_1.1 conductance that is developmentally regulated. In excitatory and inhibitory neurons derived from a child’s induced pluripotent stem cells with a *KCNT1* pathogenic variant (p.R474H), we detect gain-of-function K^+^ currents. We use an antisense oligonucleotide RNA therapy developed for two individuals with the p.R474H variant—which results in dramatic reductions in seizure occurrence and severity^1^—to profile cellular neurophysiology in patient-derived excitatory and inhibitory neurons. We observe a knockdown of p.R474H gain-of-function K^+^ currents, resulting in a stimulation-dependent change in spiking output in patient-derived induced excitatory and inhibitory neurons. In mid-gestation primary human neurons, ASO knockdown suppresses current-evoked firing, suggesting a potential early therapeutic target before the onset of infantile encephalopathy.

## Abstract (web summary)

In Golinski et al., antisense RNA silenced KCNT1, a high-mortality epilepsy gene, correcting overactive potassium currents and shaping neuron firing in patient-derived and fetal human neurons, indicating prenatal precision therapy may be feasible.

## Introduction

RNA therapeutics offer clinical opportunities for treatment-resistant neurodevelopmental disorders, including strategies to modify the RNA expression of proteins that are otherwise difficult to selectively target, such as through precision antisense oligonucleotides (ASOs)—an approach first successfully applied in the treatment of spinal muscular atrophy (SMA)^2^. Additional investigational ASO therapies for orphan neurogenetic diseases have also shown clinical success^3,4^; for example, ASO treatment was effective in two children with drug-resistant *KCNT1*-associated epilepsy of infancy with migrating focal seizures (EIMFS)^1^, a severe condition with high mortality^5,6^. Following ASO treatment, these *KCNT1* patients showed a marked reduction in seizure frequency and intensity^1^, highlighting the clinical promise of early therapeutic intervention prior to the onset of encephalopathy, loss of developmental milestones, and death^5^.

*KCNT1*-associated developmental and epileptic encephalopathy (DEE14) is a severe epilepsy syndrome affecting newborns^7^, resulting from gain-of-function (GoF) de novo variants that increase outward K^+^ currents^8,9^. In the central nervous system, *KCNT1* encodes a brain-enriched sodium-activated potassium channel (K_Na_1.1)^10^ that supports neuronal functions in the postnatal brain such as burst firing and action potential (AP) kinetics^11^. Functional characterization of a recurrent DEE14 variant (p.R474H) in *KCNT1* demonstrated a robust GoF channel activity phenotype that is resistant to clinical treatment in newborns^8,9,12^. While ion channel dysfunction is known to disrupt human prenatal processes, resulting in severe neurodevelopmental disease^13^, the onset of K_Na_1.1 during brain development and the potential for therapeutic targeting before birth, or in premature birth, is unknown.

Here, we utilized an RNase H-dependent gapmer ASO, recently applied to reduce seizures in two individuals affected with *KCNT1*-associated EIMFS^1^, to define the mechanistic recovery of AP pathophysiology in patient-derived excitatory neurons (ENs) and inhibitory neurons (INs). Furthermore, we functionally profiled the developmental emergence of K_Na_1.1 currents in primary human neurons, which can be effectively modulated using ASOs as early as 15 weeks’ gestation. The emergence of functional K_Na_1.1 during fetal brain development and its contribution to neuronal excitability *in utero* suggest that GoF variants likely contribute to disease pathophysiology before birth.

## Results

### DEE14 patient-derived ENs treated with ASO generate pronounced afterhyperpolarizations

**Fig. 1:**
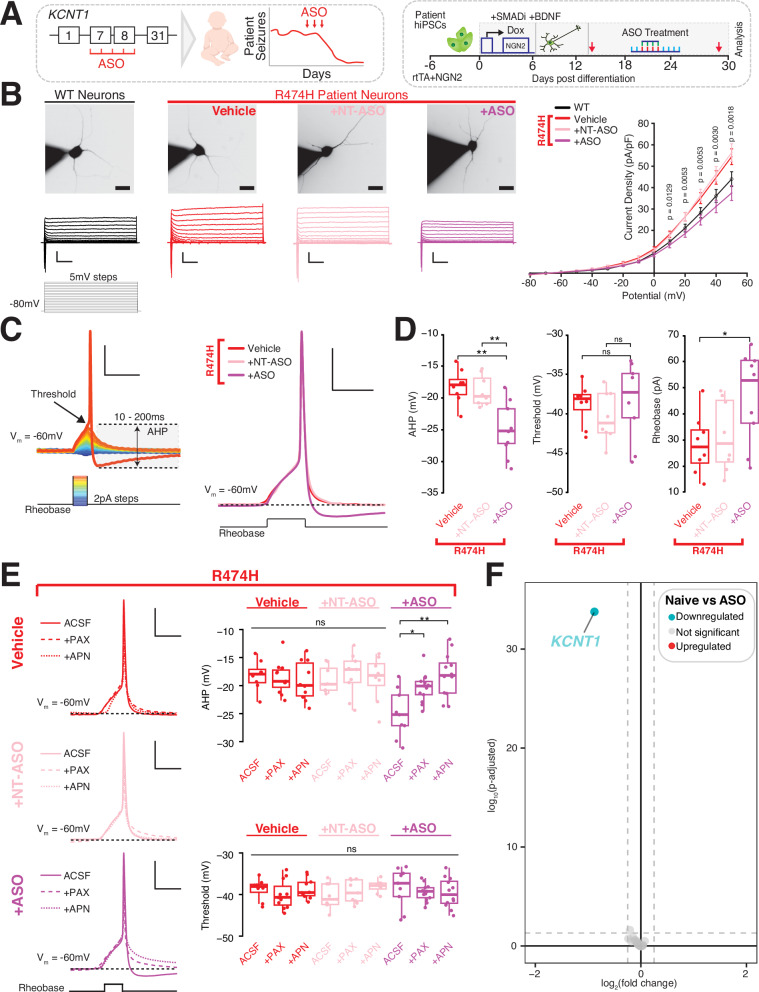
ASO-treated KCNT1-p.R474H neurons display large AHPs. **A** *Left*, ASO design (5-10-5 gapmer) targeting exons 7 and 8 of *KCNT1*, and a representative time graph of seizure reduction in a *KCNT1*-p.R474H patient following ASO treatment^1^. *Right*, schematic of NGN2 neuronal differentiation of iPSCs from a patient with the *KCNT1*-p.R474H variant**. B** *Top*, representative epifluorescence images of NGN2 neurons loaded with Alexa-Fluor 488 dye (1 µM) during patch recordings (scale bar: 10 µm). *Bottom*, voltage-clamp traces evoked by step activation routine from –80 to +50 mV at 10 mV increments (scale bars: 1 nA/100 ms). *Right*, IV plot showing WT control neurons (PGP-1 line) and KCNT1-p.R474H NGN2 neurons that have increased GoF steady-state outward K^+^ current densities sensitive to ASO knockdown (*p* = 0.0018 at 50 mV) and unaffected by control NT-ASO (*p* = 0.74 at 50 mV). *P*-values shown are between KCNT1-p.R474H and ASO-treated neurons. Line graph data presented as mean ± SEM. See Table S1 for “n” and statistics. **C** *Left*, representative AP from NGN2 neuron and sequential depolarizations leading to first firing using a rheobase routine (40 ms current injections from –5 pA to +80 pA at 2 pA increments) with *V*_*m*_ = –60 mV. *Right*, average first AP elicited across conditions, showing significantly larger mAHP in cells treated with ASO (scale bars: 20 mV/100 ms). Single-cell APs overlaid with average AP in S1. **D** *Left*, *KCNT1*-p.R474H neurons display larger mAHPs following ASO treatment (*p* = 0.013). *Middle*, average thresholds are comparable across all conditions (n.s., *p* > 0.8). *Right*, KCNT1-p.R474H neurons treated with ASO require significantly more stimulation to achieve the first AP, rheobase current (*p* = 0.036). **E** *Left*, average first AP elicited across conditions following bath perfusion of BK blocker paxilline and SK blocker apamin (PAX 500 nM and APN 50 nM, respectively), showing mAHP reduction in ASO-treated neurons. *Right*, only ASO-treated *KCNT1*-p.R474H neurons were sensitive to mAHP reduction following bath application of PAX and APN (*p* = 0.044, *p* = 0.002, respectively). Threshold values are unaffected by PAX and APN. For **D** and **E**, data presented as median withIQR in boxplots, whiskers represent the range of the data, excluding outliers. See Table S1 for “*n*” and statistics. **F** Bulk RNAseq volcano plot displays differential gene expression of AP-associated genes in control neurons following ASO treatment. Only *KCNT1* is significantly downregulated, *p* = 10^-34^ (see Tables S5 and S6 for complete raw values). Two-sample two-tailed Wilcoxon test (box plots) or two-sample two-tailed *t*-test performed, depending on sample size and normality, ^*^p < 0.05, ^**^p < 0.01. See Table S1 for patch summary statistics, and Source data are provided as a Source Data file.

The pathogenic variant *KCNT1*-p.R474H is associated with DEE14 in children with dozens to hundreds of treatment-resistant multifocal seizures per day that can be reduced in number and severity by a *KCNT1*-targeted ASO^1^, yet with limited understanding of the changes to cellular neurophysiology. To assess the effect of the ASO on p.R474H ENs, we performed voltage clamp of ENs generated via NGN2-directed differentiation from human induced pluripotent stem cells (hiPSCs) from a DEE14 individual with the p.R474H variant. After maintaining p.R474H ENs in culture for 14 days (DIV 14; see “Methods”), we treated them for 14 days (until DIV 28) with a *KCNT1*-targeting ASO (herein, ASO) or a control non-targeting sequence ASO with related thiophosphate chemistry (herein, NT-ASO). We demonstrated ASO knockdown of *KCNT1* in p.R474H and control ENs using quantitative reverse transcription polymerase chain reaction (qRT-PCR; Figs. S1B and S2B) and raw RNA counts via bulk RNA sequencing (RNA-seq; ASO-treated control ENs; Figs. 1F and S1H). In contrast, NT-ASO did not affect *KCNT1* RNA levels in either p.R474H or control ENs (Figs. S1B and S2B), confirming previous work^1^. Voltage-clamp recordings of *KCNT1*-p.R474H ENs showed an increased steady-state outward K^+^ current (consistent with GoF) compared with control ENs (PGP-1 cell line; Fig. 1B). ASO treatment of *KCNT1*-p.R474H ENs reduced the outward K^+^ current (vehicle vs. ASO: *p* = 0.002; Fig. 1B, Table S1), whereas NT-ASO did not (vehicle vs. NT-ASO: *p* = 0.7). Critically, p.R474H ENs treated with ASO, NT-ASO, or vehicle displayed comparable levels of inward sodium current density (Fig. S1D); therefore, the level of sodium activating the K^+^ current is unlikely to underlie the ASO effects. In control PGP-1 ENs, the ASO did not significantly reduce K^+^ currents (Fig. S2C; *p* = 0.474), and sodium current density was also not different (Fig. S2C). The more modest reduction of K^+^ current in PGP-1 ENs compared to p.R474H ENs is consistent with the respective *KCNT1* mRNA reductions when qPCR was performed on these lines. The ASO knockdown of mRNA levels was not significant in the PGP-1 ENs, but was significant in the p.R474H ENs, translating to a more drastic whole cell K^+^ current knockdown in the p.R474H ENs.

Quantification of AP rheobase, a value that describes the minimum input current necessary to evoke an AP at *V*_*m*_ = –60 mV, revealed an increased rheobase in *KCNT1*-p.R474H ENs treated with ASO (vehicle: 28.3 ± 4.0 pA, *n* = 8; ASO: 46.0 ± 5.6 pA, *n* = 9; *p* = 0.025; Fig. 1C, D), with NT-ASO showing a rheobase comparable to vehicle treated ENs (NT-ASO: 31.73 ± 4.79 pA, *n* = 8; *p* = 0.59; Fig. 1D, Table S1). Conversely, ASO treatment did not affect rheobase in PGP-1 ENs (Fig. S2D, *p* = 0.32). Across *KCNT1*-p.R474H ENs treated with vehicle, NT-ASO, or ASO, we did not detect changes in cell capacitance (vehicle vs. ASO: *p* = 0.565) or input resistance (vehicle vs. ASO: *p* = 0.218; Table S1). Additionally, no change was observed in capacitance or input resistance for ASO-treated PGP-1 ENs (Fig. S2C, and Table S1), suggesting ASO does not produce nonspecific effects on passive electrical properties of neurons.

Next, in ASO-treated *KCNT1*-p.R474H ENs, we analyzed the afterhyperpolarization following an AP (AHP; 10–200 ms analysis window post-AP peak), a key property of AP recovery carried by K^+^ conductance. In the current clamp, we applied the minimal current stimulation needed to elicit a single AP with a reliable AHP. We observed an increased AHP amplitude in ASO-treated *KCNT1*-p.R474H ENs compared with NT-ASO- and vehicle-treated neurons (vehicle: –18.2 ± 0.9 mV, *n* = 8; ASO: –25.1 ± 1.4 mV, *n* = 9; *p* = 0.001; Fig. 1C, D and Table S1). As AHP is measured with reference to the neuron AP threshold, we confirmed that threshold values were comparable across conditions (vehicle: –38.76 ± 0.91 mV, *n* = 8; ASO: –38.34 ± 1.595 mV, *n* = 9; *p* = 0.831; Fig. 1D and Table S1). In PGP-1 ENs, we also observed an ASO-induced increase in AHP (PGP1: –20.07 ± 1.19 mV, *n* = 9; PGP1 + ASO: –24.93 ± 1.27 mV, *n* = 10; *p* = 0.013; Fig. S2D, and Table S1), without affecting AP threshold (*p* = 0.9; Fig. S2D).

To isolate the potential compensatory ionic conductance^14^ underlying the observed ASO-induced increase in AHP amplitude, we used selective antagonists of Ca^2+^-activated large-conductance potassium (BK) channels (paxilline, PAX, 500 nM) and Ca^2+^-activated small-conductance potassium (SK) channels (K_Ca_2) (apamin, APN, 50 nM). The increased AHP observed in ASO-treated p.R474H ENs decreased significantly with PAX and, to a larger degree, with APN (Fig. 1E; baseline [ASO]: –25.05 ± 1.432 mV, *n* = 9; ASO + PAX: –20.36 ± 0.710 mV, *n* = 13, *p* = 0.022; ASO + APN: –18.107 ± 1.132 mV, *n* = 12, *p* = 0.005; see Table S1), yet we did not observe any AHP changes with PAX or APN in vehicle- or NT-ASO-treated neurons (Fig. 1E, and Table S1). Similarly, the BK and SK channel antagonists (PAX and APN, respectively) did not impact thresholds for the vehicle, NT-ASO, or ASO conditions (Fig. 1E, and Table S1). In further experiments to isolate the role of Ca^2+^ in the observed ASO-induced increase in AHP amplitude, we found that the voltage-gated Ca^2+^ channel blocker (L-Type) nifedipine (100 μM) also disrupted AHP decay kinetics (Fig. S2F, AHP area; *p* = 0.029). Another Ca^2+^-activated K^+^ channel associated with AHP kinetics in ENs is KCNQ2 (K_v_7.2), and we found that the K_v_7.2 channel antagonist XE991 reduces the AHP in ASO-treated ENs. However, in the presence of XE991 the AP threshold, which we use as reference for AHP measurement, shifted to hyperpolarized values (Fig. S2G, and Table S1), unlike with APN and PAX (Fig. 1E).

### ASO-treated ENs display *KCNT1* knockdown with action potential genes unaffected

To better understand the ASO-induced changes in AHP and excitability, we performed a bulk RNA-seq transcriptomic analysis of ASO-treated ENs. We focused on the expression of AP-associated genes (Fig. 1F), finding that *KCNT1* was the only AP gene significantly downregulated compared to vehicle (*p* = 1.7 × 10^^−34^, Table S5). Across the entire transcriptome, only 4 other genes passed *p* = <0.01 significance (Fig. S1H, and Table S6), yet were not AP related. Transcriptome-level analysis for cell death or stress-enriched pathways also did not reveal any significant transcriptomic gene ontology changes for ASO-treated ENs (Tables S5 and S6). Taken together, these results suggest that ASO-treated ENs exhibit few off target transcriptional changes.

### ASO-treated DEE14 ENs display a comparable level of neurite complexity and cell health

To analyze potential ASO-induced cytotoxicity, including changes to neuronal morphology, we performed a post-hoc morphological analysis in treated DEE14 ENs (Fig. S1C). We did not detect any differences in the ramification index, soma area, or length of the longest neurite of p.R474H or wild-type (WT) ENs among the three conditions (Fig. S1B; *p* > 0.05, Table S1), suggesting that the 14-day ASO and NT-ASO treatments do not alter complex neuronal morphology. Scholl analysis of patched ASO-treated WT (PGP1) and p.R474H ENs showed similar levels of complexity, but a significant reduction in complexity at a minority of distances from the soma (Figs. S1C and S2E).

We analyzed cytotoxicity using two common markers of apoptosis across treatment conditions, TUNEL assay and cleaved caspase-3 (c-Cas3) antibody staining. No significant differences in the number of apoptotic cells emerged between the vehicle-, NT-ASO-, and ASO-treated p.R474H ENs (Fig. S3B–E). We also found comparable levels of MAP2 complexity across vehicle-, NT-ASO-, and ASO-treated p.R474H ENs (Fig. S3C). Therefore, ~14 days of ASO treatment in culture does not appear to disrupt ENs health or result in apoptosis.

### ASO-treated DEE14 ENs display a bidirectional shift in the dynamic range of excitability

**Fig. 2:**
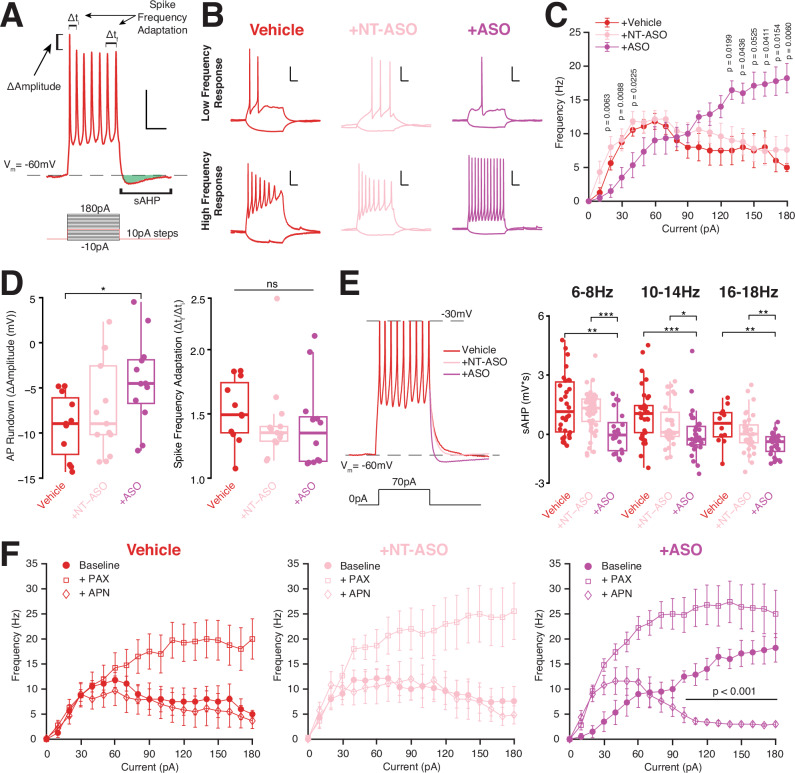
ASO-treated patient-derived KCNT1-p.R474H NGN2 ENs exhibit large sAHP and bidirectional shift in the dynamic range of stimulus evoked AP firing. **A** Representative AP burst of a KCNT1-p.R474H neuron at *V*_m_ = –60 mV in response to prolonged current injection with analysis parameters defined (–10 pA to 180 pA, 10 pA steps, 500 ms). **B** Representative AP response profiles of KCNT1-p.R474H neurons following 14-day ASO treatment protocol (vehicle, ASO, and NT-ASO) at low- and high-frequency stimulation (20 pA and 120 pA, respectively)**. C** Frequency plotted as a function of input current reveals that ASO-treated KCNT1-p.R474H neurons fire at significantly higher frequencies at large current injections compared to vehicle or NT-ASO (*p* = 0.006, *p* = 0.008, respectively). Data presented as mean ± SEM in line graph. See Table S2 for number of cells and additional statistics. **D** KCNT1-p.R474H neurons displayed a marked reduction of first-to-second AP amplitudes (ΔAmplitude, Rundown), which was recovered by the ASO treatment, without any significant effect on spike frequency adaptation. **E** Average sAHP elicited following spiking bursts for all traces between 70 pA and 90 pA (where spiking frequencies were most alike across conditions). ASO-treated neurons display increased (more negative) sAHP area. *Right*, frequency-dependent sAHP area is increased in ASO-treated neurons over a range of frequencies, including low (6–8 Hz), medium (10–14 Hz), and high (16–18 Hz) frequencies. **D**, **E** Data is median with IQR in boxplots, whiskers represent the range of the data, excluding outliers. See Table S2 for “*n*” and statistics. **F** Input–output excitability curves of vehicle-, NT-ASO-, and ASO-treated KCNT1-p.R474H neurons following bath perfusion of PAX (500 nM) and APN (50 nM) demonstrate that ASO-treated neurons rely on SK channels for maintaining firing at higher current injections, likely by enhancing AHP and delaying depolarization block. BK channel blockers enhanced firing in all conditions. Line graph data is mean ± SEM. See Table S2 for “*n*” and statistics. For all ASO treatments of NGN2 neurons (~14 days), the initial dose was 10 µM (at DIV 14), with a second 5 µM maintenance dose at DIV 21. Data are presented as mean ± SEM. Two-sample two-tailed Wilcoxon test (box plots) or two-sample two-tailed *t*-test performed, depending on sample size and normality, ^*^p < 0.05, ^**^p < 0.01, ^***^p < 0.001. See Table S2 for statistics, and Source data are provided as a Source Data file.

K_Na_1.1 regulates neuronal burst rate and AP kinetics^11^, including AHP kinetics and maintenance of rhythmic bursting during sustained depolarization^15^. To characterize the bursting and excitability properties of *KCNT1*-p.R474H ENs treated with ASO, we injected a wide range of currents (–10 to 180 pA in 10-pA steps) to generate sustained depolarizations and AP firing (Fig. 2A). In ASO-treated p.R474H ENs, we observed a bidirectional shift in the input–output AP firing curves compared with vehicle- and NT-ASO-treated ENs. At low current injections, ASO treatment reduced excitability (at 40 pA, vehicle: 10.55 ± 0.86 APs, *n* = 10; ASO: 5.33 ± 1.85 APs, *n* = 12; *p* = 0.023; Fig. 2C); however, at higher current injections, ASO-treated ENs fired at higher frequencies than vehicle- and NT-ASO-treated ENs (at 180 pA, vehicle: 5.00 ± 0.58 APs, *n* = 10; ASO: 18.22 ± 2.17 APs, *n* = 12; *p* = 0.006; Figs. 2C and S4C). To look closer at AP kinetic features, ASO treatment reduced the AP amplitude run-down between spikes, yet did not significantly alter spike frequency adaptation (Fig. 2D). ASO treatment of WT ENs also increased maximum AP firing at high current injections (Fig. S4B).

Because ASO-treated *KCNT1*-p.R474H ENs displayed reduced run-down and increased dynamic range of input–output AP spiking (Fig. 2C, D), we next explored the slow AHP (sAHP). sAHPs are frequency-dependent and critical to neuron firing, as they are generated following trains of synaptic input and burst AP firing, and are carried by Ca^2+^-dependent K^+^ channels^16^. ASO-treated *KCNT1*-p.R474H ENs displayed larger sAHPs than vehicle- and NT-ASO-treated ENs across a range of firing frequencies (*p* = 0.002 at 6–8 Hz; *p* < 0.001 at 10–14 Hz; and *p* = 0.002 at 16–18 Hz; Fig. 2E). ASO-treated WT ENs also displayed an increase in sAHP (Fig. S4B).

Next, we pharmacologically isolated potential ionic conductances underlying ASO increased maximal response of AP firing dynamics using BK and SK channel blockers (PAX, 500 nM; APN, 50 nM, respectively). Intriguingly, while the BK antagonist (PAX) increased high-frequency firing across all three conditions (vehicle, NT-ASO, and ASO), the SK blocker (APN) reduced high-frequency AP firing only in the ASO-treated *KCNT1*-p.R474H condition (Fig. 2F, and Table S2). Moreover, in ASO-treated ENs, the KCNQ2 (K_v_7.2) channel antagonist XE991 also did not reduce the AP input–output curve, but instead increased high-frequency firing (ASO vs. ASO + XE991: *p* < 0.05 at all current injections; Fig. S4D). These highly selective pharmacological treatments indicate that ASO-treated *KCNT1*-p.R474H ENs likely rely on Ca^2+^-activated SK channels (K_Ca_2) to support the observed high-frequency AP firing.

### *KCNT1* is expressed in mid-gestation human cortical plate pyramidal neurons

**Fig. 3:**
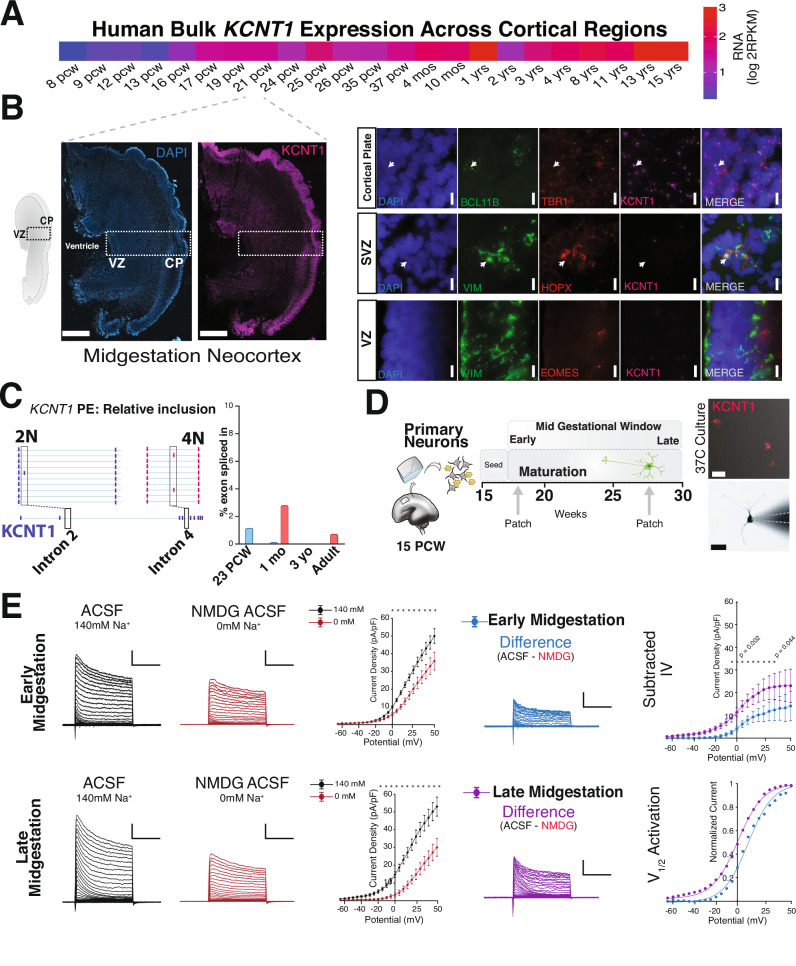
Early expression of KCNT1 and developmental emergence of Slack currents in mid-gestation primary human neurons. **A** Analysis of *KCNT1* expression during human brain development from 8 PCW to late adolescence. **B** *Top*, *KCNT1* RNA in situ hybridization of a 21 PCW human coronal brain section from the perisylvian region demonstrates robust *KCNT1* enrichment in the cortical plate (CP). *Bottom*, spatial transcriptomic analysis of *KCNT1* expression within specific CP markers, including a neuronal marker (RBFOX3) and deep layer markers CTIP2 (BCL11B) and TBR1, with limited *KCNT1* expression in dividing cell types, including oRGCs and progenitor markers (*HOPX, EOMES*, VIM). SP, subplate; SVZ, subventricular zone; VZ, ventricular zone (scale bars: Top, 100 μm; Bottom, 10  μm). Technical replicates *n* = 3. **C** Analysis of rare inclusion of poison exons in *KCNT1* transcripts in the human brain. *Left*, example of reads in Integrative Genomics Viewer (IGV) showing rare inclusion of unannotated exons in intron 2 (top) and intron 4 (bottom). In silico translation of poison exons inserted in-frame into the canonical *KCNT1* transcript introduces premature termination codons. *Right*, percentage spliced-in of *KCNT1* poison exons in human fetal and postnatal brain, as estimated by targeted long-read sequencing of RT-PCR products. (*N* = 1 brain sample per time point). **D** *Left*, experimental schematic of primary neuron plating (15 PCW) and differentiation in cell culture from a healthy individual. *Right*, representative epifluorescence images of a primary neuron demonstrating *KCNT1* expression (red signal, in situ*)* and a dye-loaded neuron during patch recordings (Alexa-Fluor 488 dye, 1 µM) for morphological quantification (scale bars: 10 μm). **E** Representative voltage-clamp recordings of outward K^+^ currents from mid-gestational primary human neurons evoked by an activation routine from –80 mV to +50 mV at 5 mV increments (scale bars: 1 nA/100 ms) in external ACSF with physiological sodium (140 mM) and sodium-free ACSF (NMDG-ACSF, 0 mM Na^+^). IV plot of K^+^ currents in ACSF (140 mm Na^+^) and NMDG-ACSF (0 mM Na^+^) show that K_Na_ current partially underlies the observed increase in steady-state outward current during maturation. Data presented as mean ± SEM in line graph. See Table S3 for number of cells and additional statistics. Boltzmann curves fit to the Na^+^-dependent outward current (total K^+^ current in ACSF minus the sodium-insensitive K^+^ current NMDG-ACSF) show a leftward shift in V_1/2_ activation during the mid-gestational period (95% confidence intervals for nonlinear least-squares parameter estimates). Bulk RNA data from ABA^20^. Two-sample two-tailed *t*-test performed. See Table S3 for complete statistics and comparisons, and Source data are provided as a Source Data file.

Early diagnosis and intervention for epilepsy disorders improve clinical outcomes^17^. DEE14-associated EIMFS are typically observed within 6 months after birth^18,19^, suggesting that early pathology might occur before seizure onset. Therefore, we sought to define a cell-type-specific prenatal profile of *KCNT1* leading up to the neonatal period. Bulk cortical human transcriptome data from the Allen Brain Atlas^20^, from 12 weeks post-conception (PCW) to late adolescence, indicate that *KCNT1* expression is first observed mid-gestation (13–28 PCW) and persists thereafter (Fig. 3A). This developmental emergence of *KCNT1* expression coincides with several overlapping processes—including proliferation, neuronal migration, and differentiation—which can be disrupted by ion channel dysfunction^13^. To investigate *KCNT1* cell-type distribution and potential spatiotemporal-dependent pathology, we performed spatial RNA profiling in the human fetal neocortex during the mid-gestation *KCNT1* onset window (15–21 PCW). Multiplexed fluorescent in situ hybridization analysis of *KCNT1* co-expression within developmental cell types along a neocortical column revealed that *KCNT1* mRNA was not present in the progenitor germinal zones, including within radial glia or immediate progenitors (*EOMES-* and Vimentin-positive cells) of the ventricular and intermediate zones (Fig. 3B). Similarly, we did not observe *KCNT1* expression in progenitors of the outer subventricular zone or within the primate-enriched outer radial glial cells (HOPX^+^) (Fig. 3B). The primary *KCNT1* mid-gestational expression occurs within postmitotic neurons (RBFOX3^+^) in the early cortical plate (Fig. 3). We did not observe significant *KCNT1* expression within immature migrating neurons (Fig. 3B) or subplate neurons (Fig. S5A), in contrast to the cell-type expression patterns observed for other early epilepsy-related ion channel diseases^21^. Neuron-subtype-specific markers revealed *KCNT1* expression within several excitatory cell types in the cortical plate, including deep-layer neuron markers TBR1 and CTIP2 (*BCL11B*) (Fig. 3B). *KCNT1* signal was negative in glial fibrillary acidic protein (GFAP)-positive cells throughout the deep to superficial cortex (Fig. S5B). These results indicate that *KCNT1* is primarily expressed within deep-layer postmitotic neurons in the cortical plate, and does not appear to be expressed in progenitor or glial cells or immature neurons in the perisylvian coronal neocortical column.

While we detect *KCNT1* RNA in developing neocortex tissue, poison exons are naturally occurring genetic elements that introduce premature termination codons^22^. We therefore used targeted long-read sequencing of *KCNT1* transcripts, for evidence of poison exons in neocortical samples across the pre- and post-natal period. We analyzed isolated RNA from the neocortex at four time points (23 PCW, 1 month old, 3 years old, and adult) for two putative poison exons in intron 2 and intron 4 of *KCNT1*, respectively. However, these poison exons were included in fewer than 5% of reads across all ages tested (Figs. 3C and S5F), suggesting that they are unlikely to substantially contribute to the regulation of overall *KCNT1* transcript levels and a poor therapeutic target.

### Increase in sodium-activated K^+^ channels (K_Na_1.1) in the mid-gestation human neocortex

To determine the prenatal emergence of functional K_Na_1.1, we performed patch-clamp recording on primary human neurons isolated from the 15 PCW neocortex (Fig. 3D). We applied voltage-step protocols to elicit steady-state outward K^+^ channel currents over a range of membrane potentials (–80 to +60 mV). To isolate the sodium-activated K^+^ conductance (K_Na_) from the total outward steady-state K^+^ current, we performed patch-clamp experiments in both regular artificial cerebrospinal fluid (ACSF) and in sodium-free ACSF, in which sodium was replaced with an equimolar concentration of N-methyl-D-glucamine (NMDG-ACSF; see “Methods”). To quantify the sodium-activated K^+^ current, we subtracted the sodium-insensitive current (in NMDG-ACSF) from the total K^+^ current (in regular ACSF), leaving only the sodium-sensitive K^+^ current as the difference. In these experiments, the internal recording solution did not contain sodium, ensuring that K_Na_ activation originated from sodium influx into neurons. The sodium-sensitive outward K^+^ current was present as early as 15 PCW (early mid-gestation) and increased in amplitude as primary neurons matured in culture over 2–3 months (Fig. 3E; early vs. late, voltage steps from –25 to +15 mV, *p* < 0.05, *n* = 13 and *n* = 21, respectively; Table S3).

K_Na_1.1 channels are also voltage-sensitive, and epilepsy-associated variants can result in shifts in the voltage dependence of channel opening^23^. Therefore, we determined the half-activation voltage (V_1/2_) of K_Na_1.1 in mid-gestational primary human neurons. Performing voltage steps in both NMDG-ACSF (0 mM sodium) and regular ACSF (140 mM sodium), we isolated the V_1/2_ of the sodium-sensitive portion of the outward current and observed a ~10-mV leftward shift in activation kinetics at later mid-gestational time points (17 PCW: V_1/2_ = 10.03 ± 1.70 mV; 28 PCW: V_1/2_ = 0.39 ± 1.14 mV, shown as 95% confidence intervals; Fig. 3E). These results suggest that in more mature neurons, the open probability of K_Na_1.1 is higher at more hyperpolarized potentials, resulting in K_Na_1.1 activation closer to V_m_. Taken together, the observed increase in sodium-sensitive current and leftward shift in V_1/2_ activation during gestational maturation suggest that *KCNT1* has an early and temporally shifting function during development. Therefore, DEE14 variants that affect K_Na_1.1 biophysics likely alter prenatal neurophysiology.

### ASO knockdown of K_Na_1.1 in mid-gestational human fetal neurons

**Fig. 4:**
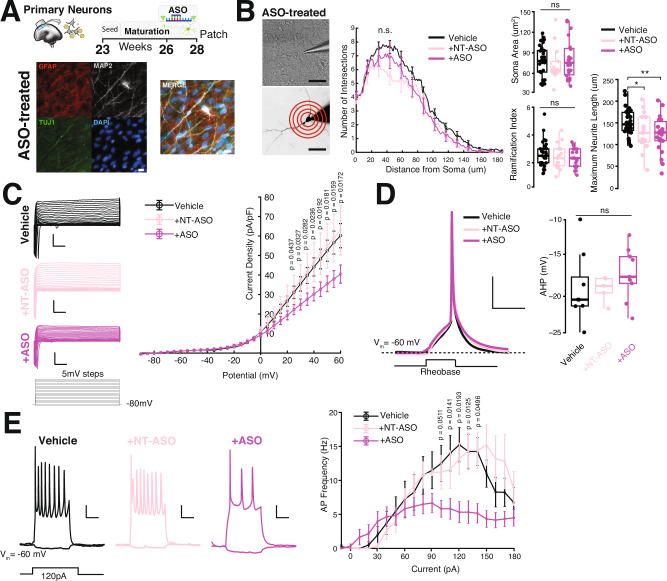
ASO knockdown of K_Na_1.1 currents (KCNT1) in human fetal neurons reduces excitability. **A** *Top*, schematic of primary neuron isolation from mid-gestation human cortex and maturation timeline. *Bottom*, representative immunofluorescence images of primary neurons treated with ASO (14 days) and vehicle, with antibody labeling of neuronal-specific markers, microtubule-associated protein 2 (MAP2) and beta-tubulin III (TUJ1), and the astrocyte marker GFAP (scale bars: 10 µm). Technical replicates = 4. **B** *Left*, representative brightfield and epifluorescence images of ASO-treated primary neurons loaded with Alexa 488 dye (1 µM) with representative Sholl analysis (scale bars: 50 µm). *Right*, analysis of neuromorphological properties of mid-gestation neurons demonstrates comparable neuronal complexity, ramification, and soma size following vehicle, NT-ASO, or ASO treatment. Maximum neurite length was significantly lower in cells treated with ASO or NT-ASO (*p* = 0.0089, *p* = 0.05, respectively). Line graph data is mean ± SEM. Median with IQR in boxplots, whiskers represent the range of the data, excluding outliers. See Table S3 for “n” and statistics. **C** Representative voltage-clamp traces from mid-gestation neurons evoked by a step activation routine, –90 mV to +60 mV at 5 mV increments (scale bars: 1 nA/100 ms). Healthy primary fetal neurons (15 PCW sample) exhibit steady-state outward K^+^ currents sensitive to ASO knockdown (10 µM for 14 days) (*p* = 0.0321 at 60 mV) and are unaffected by control NT-ASO (*p* = 0.7827 at 60 mV). Line graph data is mean ± SEM. See Table S3 for “*n*” and statistics**. D** Analysis of average first AP elicited during the rheobase routine following 14-day ASO treatment. AP kinetics, including the mAHP, are unaffected by the ASO treatment. Median with IQR in boxplots, whiskers represent the range of the data, excluding outliers. See Table S3 for “*n*” and statistics. **E** *Left*, representative maximum spiking of mid-gestation neurons following ASO treatment. *Right*, ASO-treated primary neurons displayed decreased AP firing over a range of current injections and exhibited maximum firing frequency at lower current injections than vehicle- and NT-ASO-treated cells (*p* = 0.012). Data is mean ± SEM in line graph. See Table S3 for “*n*” and statistics. Two-sample two-tailed Wilcoxon test (box plots) or two-sample two-tailed *t*-test performed, depending on sample size and normality. ^*^p < 0.05, ^**^p < 0.01. See Table S4 for summary statistics and Source data are provided as a Source Data file.

We next investigated ASO knockdown of *KCNT1* in primary human neurons from mid-gestation neocortex. We isolated primary neurons from 15- and 23-post-conception-week neocortices and maintained them in culture for 10 weeks (Figs. 4 and S6). We performed ASO treatment for 14 days prior to patch-clamp recording to ensure *KCNT1* knockdown. Analysis of the complexity and health of primary neurons following ASO treatment of patch-clamp-recorded neurons showed no change in dendritic complexity (as measured by Sholl analysis), soma size, or ramification index between vehicle-, NT-ASO-, and ASO-treated neurons (Fig. 4B, and Table S3; *p* > 0.873). However, NT-ASO and ASO treatment both resulted in a mild reduction of the longest neurite length (Fig. 4B; vehicle vs. ASO: *p* = 0.027; vehicle vs. NT-ASO: *p* = 0.050). Using voltage clamp, we demonstrated a significant ASO-mediated reduction of steady-state outward K^+^ currents at depolarized potentials (25 to 60 mV) compared with vehicle- and NT-ASO-treated neurons (Fig. 4C; e.g., at 60 mV, vehicle: 58.02 ± 5.93 pA/pF; ASO: 43.28 ± 2.72 pA/pF; vehicle vs. ASO: *p* = 0.0321). Capacitance values were comparable across all three conditions (vehicle: 31.97 ± 3.20 pF; ASO: 32.63 ± 3.15 pF; NT-ASO: 27.89 ± 3.89 pF; *p* > 0.05). We observed this ASO K^+^ current knockdown in both fetal samples treated (Fig. S6B), yet ASO did not affect sodium channel current density and kinetics (Fig. S6C). In ASO-treated primary fetal neurons, cells exhibited a reduced capacity for repetitive firing over midrange to high current injections (100–150 pA) compared to Vehicle and NT-ASO-treated cells, resulting in a flattened frequency curve (Fig. 4E). Taken together, these results suggest that ASO treatment can be used to modulate K_Na_1.1 currents in primary human fetal neurons, suppressing excitability.

### *KCNT1* is enriched in both INs and ENs in the early childhood neocortex

**Fig. 5:**
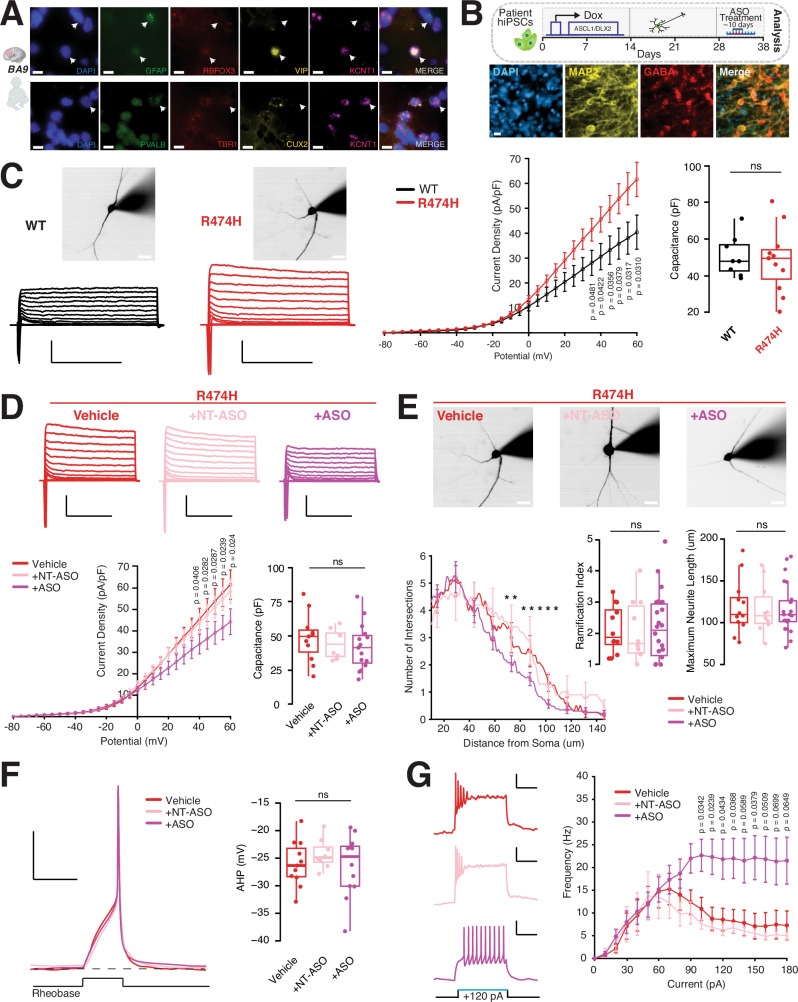
KCNT1-p.R474H iGABA neurons demonstrate improved dynamic range of sustained AP firing following ASO treatment. **A** Analysis of *KCNT1* expression and cell-type markers in the toddler cortex (3 years old), including enrichment in INs (parvalbumin- and VIP-expressing, PVALB, VIP) and *KCNT1* expression in ENs (TBR1^+^ and CUX2^+^), with limited expression within the glial marker GFAP, scale bars: 10 μm. Technical replicates = 3. **B** *Top*, schematic of iGABA neuronal differentiation of iPSCs from a patient with the KCNT1-p.R474H variant. *Bottom*, representative immunofluorescence images of neurons following a 35-day iGABA differentiation protocol, with antibody labeling of the neuronal-specific marker microtubule-associated protein 2 (MAP2) and GABA marker anti-GABA, scale bars: 10 μm. Technical replicates = 4. **C** *Left*, representative voltage-clamp traces evoked by step activation routine from −90 mV to +60 mV at 5 mV increments, and representative iGABA neurons loaded with Alexa-Fluor 488 dye during patch-clamp assays. *Right*, IV curves of WT and KCNT1-p.R474H iGABA neurons showing the GoF steady-state outward K^+^ currents. All currents were capacitance-normalized, and cell capacitances were comparable across conditions, scale bars: 20 μm. **D** *Top*, representative voltage-clamp traces evoked by step activation routine from −90 mV to +60 mV at 5 mV increments under vehicle, NT-ASO, and knockdown ASO conditions. *Bottom*, IV curves display ASO knockdown of outward steady-state K^+^ current. **E** *Top*, representative iGABA neurons loaded with Alexa-Fluor 488. *Bottom*, Sholl analysis of p.R474H iGABA neurons treated with vehicle, NT-ASO, and ASO shows comparable morphology, scale bars: 20 μm. **F** Average AP of iGABA p.R474H neurons following ASO treatments shows no change in AHP amplitude. **G** *Left*, representative spiking traces of p.R474H iGABA neurons during 120 pA current injection. *Right*, spiking frequency curves of p.R474H iGABA neurons following ASO treatments in response to 0 pA to 180 pA current injections. ASO-treated iGABA neurons display significantly higher spiking frequencies than vehicle- and NT-ASO-treated neurons at higher current injections. **C**–**F** Line graph data is mean ± SEM. Data presented as median with IQR in boxplots, whiskers represent the range of the data, excluding outliers. See Table S4 for “*n*” and statistics. Two-sample two-tailed Wilcoxon test (box plots) or two-sample two-tailed *t*-test performed, depending on sample size and normality. ^*^p < 0.05, ^**^p < 0.01. See Table S4 for summary statistics, and Source data are provided as a Source Data file.

Given the severity of *KCNT1*-associated epilepsy in infants—including increased risk of mortality by 5 years of age and severe intellectual disability^6^—we analyzed postnatal human neocortex for *KCNT1*-enriched cell types. We performed multiplexed spatial RNA profiling in an infant neocortex (age: 7 months) and a toddler neocortex (age: 3 years) (Brodmann area: BA9). In the infant neocortex, *KCNT1* was enriched in several neuronal subtypes, including high expression in interneuron subtypes such as somatostatin (SST^+^), vasoactive intestinal peptide (VIP^+^), and parvalbumin (PVALB^+^) subtypes (Fig. S5B). Similarly, in an older neocortex sample (3-year-old individual, healthy), *KCNT1* was also co-enriched in INs, including the same interneuron types observed in the infant neocortex (PVALB^+^, SST^+^, VIP^+^; Fig. 5A). Neither the infant nor toddler sample showed significant KCNT1 expression in GFAP^+^ cells (Figs. 5A and S5B). These results suggest that broad *KCNT1* enrichment across several neuronal subtypes during the early postnatal period, including both excitatory and inhibitory types, likely underlies postnatal developmental pathology.

### ASO-treated *KCNT1*-p.R474H iGABA INs display increased high-frequency AP firing properties

Considering the observed enrichment of *KCNT1* in INs in the early childhood neocortex, we next characterized *KCNT1*-p.R474H INs using a protocol to generate iGABA-INs^24^. After differentiating p.R474H iGABA-INs in culture for 28 days (DIV 28), we observed robust GABA staining across neurons (Fig. 5B) via immunohistochemistry and performed patch clamp at DIV 34. Voltage-clamp recordings of *KCNT1*-p.R474H iGABA-INs showed an increased steady-state outward K^+^ current (GoF) compared with control iGABA-INs (PGP-1 cell line, *p* = 0.031; Fig. 5C). In current clamp, we observed shifted kinetics of AP downstroke and threshold for iGABA-INs (p.R474H vs. PGP-1, *p* = 0.035; Fig. S7B), while rheobase, AHP, and amplitude remained comparable (Figs. 5F and S7B, and Table S4).

Next, we treated iGABA-INs for 10 days (DIV 28–38) with a *KCNT1*-targeting ASO or NT-ASO. ASO treatment of p.R474H iGABA-INs reduced outward K^+^ current (vehicle vs. ASO: *p* = 0.024; Fig. 5D), whereas NT-ASO did not affect K^+^ current. Unlike p.R474H ENs, iGABA-INs treated with ASO exhibited comparable rheobase and AHP across conditions (*p* = 0.726, *p* = 0.852, respectively; Figs. 5F and S7D, and Table S4). Across iGABA-INs treated with NT-ASO or ASO, we did not detect changes in cell capacitance (vehicle vs. ASO: *p* = 0.365) or input resistance (vehicle vs. ASO: *p* = 0.484 Table S4). To analyze potential ASO-induced changes in neuronal morphology in ASO- and NT-ASO-treated INs, we performed a post-hoc morphological analysis after 12 days of ASO treatment, and did not detect any differences in the ramification index or length of the longest neurite among the three conditions (vehicle, ASO, NT-ASO; Fig. 5E, *p* = 0.62; Table S4), suggesting that ASO treatments do not drastically change the neuronal morphology of iGABA-INs.

To characterize the excitability of iGABA-INs, we injected a wide range of currents (10-pA steps) to generate sustained depolarizations and APs. In ASO-treated p.R474H iGABA-INs, we observed an increase in stimulus-evoked action potential firing across a range of high current injections, and AP amplitude is more reliably recovered from first to second spike (less rundown, see Fig. S7E), compared with NT-ASO-treated iGABA-INs (*p* = 0.038 at +150 pA; Fig. 5E, and Table S4).

## Discussion

In this study, we identified the neurophysiological basis of a first-in-human RNA therapy for children with treatment-resistant *KCNT1*-related epilepsy (DEE14)^1^. We identified the emergence of functional K_Na_1.1 during human mid-gestational development and showed that novel ASOs, used to treat DEE14, can effectively modulate K_Na_1.1 currents at this fetal time point. Furthermore, we found that ASO treatment can increase the maximal response for input-output excitability curves in *DEE14* patient-derived ENs and INs. Taken together, our results suggest that early, prenatal targeting of K_Na_1.1 could improve neurophysiological phenotypes and potentially improve clinical outcomes.

Prenatal ion channel dysfunction is associated with severe neurodevelopmental disorders^13^, and *KCNT1* variants likely share a convergent role in neocortical pathophysiology leading up to the first phenotypes (seizures), observed shortly after birth. The observed *KCNT1* enrichment in mid-gestation cortical plate neurons and the presence of K_Na_1.1 currents, as early as 15 PCW suggest an early and temporally sensitive K_Na_ contribution. The increase in current size and shift in voltage dependence of activation (V_1/2_, closer to resting V_m_) as neurons mature could be explained by expression of alternative *KCNT1* splice isoforms, shifting protein binding partners, including a differential expression of KCNT2, which forms heteromeric channels with KCNT1^25,26^. Interestingly, unlike other ion channels enriched in the prenatal human brain associated with disease (e.g., *SCN3A, GRIN2B*), *KCNT1* is not expressed in neocortical progenitors, key cell types associated with severe brain malformations^13,27^, yet mild malformations have been reported^28^. When considered alongside the postnatal microcephaly observed in *KCNT1* patients^8^, our functional K_Na_1.1 recordings suggest that the prenatal DEE14 pathology is primarily neuronal.

In patient-derived DEE14 ENs and INs, the input-output excitability curve shows an increased maximal spiking response (more APs at high current injections) following ASO treatment, likely mediated by decreased AP amplitude run-down between spikes and reduced depolarization block. Several functions of K_Na_ activation in neurons include the shaping AP spiking that shifts cellular excitability and inter-burst timing^11,29,30^, and enlarging AHP after a single spike to enhance burst firing during prolonged current stimulation^15,31^. Therefore, the observed ASO-associated suppressed excitability in ENs (at minimal stimulation, below 50pA) and primary fetal neurons supports previous models for KCNT1’s direct effect on excitability. However, we found that ASO treatment of both DEE14 and control NGN2 ENs showed an unexpected compensation: increased AHP amplitude. The observed AHP results are paradoxical, as a reduction in the outward K^+^ current by the ASO should also decrease the size of the AHP. Yet, in various neuron subtypes, AHP can be carried by different ion channel currents with overlapping fast, medium, and slow AHP kinetics^16,32^, as observed by the pharmacological isolation of Ca^2+^-activated K^+^ channel (BK and SK) AHP effects in this study. Several other channel mechanisms could contribute to the ASO-induced AHP enhancement, such as homeostatic compensations in NGN2 ENs^33^, activity-dependent compensations^14,34^, as seen with BK channels^35^, or the observed downregulation of *KSR1*, a RAS-RAF-MEK-ERK signaling pathway, which can affect AHP^36^. Regardless, this enhanced AHP is a potential mechanism underlying the increased capacity for spiking at higher current injections in DEE14 and control NGN2 ENs. With the larger AHP amplitude following an AP, high stimulation firing is supported by reducing the cell’s susceptibility to depolarization block, allowing higher frequency firing where cells would otherwise typically begin to saturate. Additionally, while we did not observe significant changes in sodium channel kinetics following ASO treatment, shifts in protein localization of voltage-gated sodium channels, as observed in kcnt1-R455H mice^37^, or sensitivity of SK channels to small changes in excitation^38^, may further contribute to the deficits associated with DEE14. Nevertheless, these findings highlight the ability of AHP modulation to shape cellular physiology, and follow-up studies exploring therapeutic AHP enhancement as a biomarker—particularly in vivo to support complex spiking activity—might provide a basis for understanding a broad range of disorders with disrupted cellular excitability.

How pathogenic K^+^ channel variants can differentially affect various cell types is an emerging area of research^37,39,40^. For example, while NGN2 ENs transcriptionally most closely resemble early cortical ENs^41^, in our study, NGN2 neurons had larger K_Na_1.1 currents than primary human neurons, suggesting that cortical neurons possess different protein co-expression networks or represent different stages of maturation. Here, ASO treatment of primary human non-pathophysiological neurons reduced excitability at suprathreshold current injections. In a different DEE14 GoF variant model (P924L)^42^, less mature induced neurons (<20 days differentiation) were shown to have elevated firing rates, but in this case, due to increased K_Na_. Because our ASO suppresses EN AP firing rates at low current injections, these patients could potentially benefit from our ASO treatment. In contrast to the observed ASO-increased AHP in NGN2 neurons (from both patient and control cell lines), iGABA neurons showed no change in AHP yet demonstrated increased high-frequency firing. Therefore, like other studies^39^, our differential results across NGN2, primary, and iGABA neurons highlight the cell-type-specific functions of K^+^ channels and potential differential pathology of associated *KCNT1* variants. Additionally, this study only investigated one dosing routine across all cell types. It is possible, however, that different cell types, and by extension different individuals, would have unique dosing requirements to achieve certain results. Follow-up studies should be performed to assess potential non-conducting roles of *KCNT1* in both NGN2 and primary human fetal neurons, such as RNA translation, cellular signaling, modulation by voltage-gated sodium channels^43^, and function at the mitochondrial membrane^44^. Exploring these downstream or alternative disease mechanisms could provide additional insights into the role of *KCNT1* in normal development and could inform the design of biomarkers and enable the treatment of K_Na_1.1 GoF phenotypes.

The *KCNT1* enrichment observed within INs from the infant and toddler human brain (most abundant in PVALB^+^ INs) likely supports a key physiological role for K_Na_1.1 in GABAergic refinement of neocortical circuits, a proposed mechanism responsible for EIMFS^9^. Rodent electrophysiology data have shown that cortical GABAergic neurons expressing GoF *KCNT1* variants display lower excitability^37,45^, resulting in a disrupted excitatory/inhibitory balance, which generates network hyperexcitability^45^. Mouse models of infantile and epileptic spasms syndrome show altered Pvalb^+^ interneuron development and GABAergic synaptic dysfunction throughout life^46^. For example, Pvalb^+^ interneurons fail to fire repetitively with high current stimuli and are prone to depolarization block in *kcnt1*-L456F mice^47^. Moreover, differential *kcnt1* pathophysiology exists even within related GABAergic subtypes (SST, PVALB, VIP), resulting in opposing functional deficits^40^. In the context of potential shared interneuronopathy mechanisms, *kcnt1*-ASO can also increase survivability in both *Scn1a* and *Scn8a* mutant mice^48^. Lastly, as humans and mice have different representations of gene expression across different INs^49^, species-specific effects could exist. Taken together, our in vitro work on human ENs and INs and others^42^ suggests a shared in vivo mechanism that may facilitate enhanced network inhibition via a shifted input–output function by ASO reduction of K_Na_1.1.

K_Na_1.1 dysfunction is associated with highly heterogeneous treatment-resistant epilepsy immediately following birth (i.e., EIMFS), and nonselective drugs fail to normalize developmental delay^50–52^. Even with the use of more selective drugs, neonatal epilepsies have poor outcomes^17^, necessitating a shift to targeted genetic therapies^6^. One promising treatment modality in humans to delay disease onset is *in utero* treatment, including for SMA^53^ and for non-brain diseases^54^. For prenatal treatment, diagnosis is a limiting factor for early detection, as current prenatal screening (e.g., cell-free fetal DNA, ~12 PCW) detects large genomic changes (e.g., copy number variations), or less commonly, a targeted single-gene approach^55^, as with recent *in utero* diagnosis and treatment of SMA^53^. For infants not diagnosed prenatally, newborns can receive rapid clinical exome or genome sequencing^56^, offering genetic diagnosis within days. While ASOs in our in vitro study did not result in cell death or gross dysmorphology, intrathecally delivered RNase-H-dependent ASOs have been associated with adverse outcomes, such as hydrocephalus^57,58^, including the *KCNT1* ASOs presented here^1^. Presymptomatic ASO treatment in neonatal kcnt1-p.P924L GoF mice was considered effective and safe, with improvements in cognition and behavior^59^. These findings warrant future studies to explore the use of ASOs in early perinatal clinical treatments for *KCNT1*-associated disorders to provide early neuroprotection against K_Na_1.1-GoF-associated encephalopathy and developmental delay.

### Limitations

In this study, we characterized the intrinsic physiology of DEE14 patient-derived EN and INs and modulation by ASOs; however, we did not explore network-level effects which could influence excitatory/inhibitory balance. In future experiments, mixed EN:IN cultures could improve phenotyping and ASO selection. While we showed functional KCNT1 currents in fetal brain neurons at 15 and 23 PCWs, our in vitro culture maturation system to “28 PCW equivalent” gestation might not reflect the cell fate/maturation conditions observed during brain development in vivo. Therefore, conclusions on the growth in K_Na_ current size and voltage dependence of *KCNT1* currents in vitro over time are limited. Lastly, while our in vitro data revealed a robust ASO-induced KCNT1 knockdown, there could be different homeostatic mechanisms between NGN2-induced neurons and primary neurons, including the compensatory Ca^2^+ -activated AHP. Also, even within the very refined cell types we studied (DEE vs Control NGN2), we observed considerable phenotypic inter-line variability, which might shape decisions for precision medicine at the individual level. However, of critical importance, the convergent ASO effect on AP spiking output of DEE14 INs and ENs was to increase AP firing in response to large current stimuli. Together, the varied ASO response profiles across neuron types suggest future studies should be conducted on multiple model systems, including native tissue.

## Methods

### Ethics, human participants, and sample collection

Human research was conducted according to protocols approved by the institutional review boards of Northwestern University and Boston Children’s Hospital. Studies using patient-derived induced pluripotent stem cells (p.R474H-KCNT1) were conducted with written informed participant/family consent under Boston Children’s Hospital IRB–approved protocols and administered by the Manton Center for Orphan Disease Research Gene Discovery Core, conducted under BCH IRB–approved protocols (IRB-P00035937; IRB-P00037661). No compensation was provided. Human fetal tissue used in this study was obtained as secondary clinical material through the Northwestern’s Pathology Department following pathological clinical collection and workup, and was released for research through the Clinical Specimen Release Request process. The Northwestern University Institutional Review Board reviewed the study “Early developmental disorders of the nervous system; using primary human tissue to study molecular mechanisms of disease” (IRB ID STU00217491) and determined that the proposed activity did not constitute research involving human subjects; therefore, further IRB review and study-specific written informed consent were not required. Investigators did not collect tissue directly from donors, had no direct contact with donors, and received only deidentified specimens after clinical/pathological evaluation. The research team had no access to direct identifiers or to information that could readily be used to ascertain donor identity. Tissue was donated for research purposes via the clinical specimen release protocol following pathological clinical collection and workup, in accordance with institutional procedures governing release of clinical specimens for research. Fetal brain tissue was received after release from clinical pathology, with a maximum post-mortem interval of 4 h. Fetal cases with known anomalies were excluded, with two male samples include in this study. Tissue was transported in ice-cold Hibernate-E medium (Thermo Fisher) for processing in the laboratory. The neonatal (7 months) and 3-year-old brain samples were obtained from the University of Maryland Brain and Tissue Bank of the NIH NeuroBioBank (sample numbers UMBN 4353 and HCT17HEIA029, respectively) and stored at –80 °C until processing.

### Human-derived induced pluripotent stem cell lines with p.R474H-KCNT1

The female donor’s iPSCs were derived from skin fibroblasts and reprogrammed via Sendai virus with a normal karyotype, as previously described^1^. iPSC colonies were maintained in mTESR1 Plus media (Stem Cell Technologies) on Geltrex (Thermo Fisher)-coated plates and passaged every 5–7 days, and the media was changed every other day. The iPSCs were tested as mycoplasma-negative. Pluripotency was confirmed with qRT-PCR, and markers OCT4, NANOG, and TRA1-60 were detected using immunocytochemistry. Publicly available iPSC lines reprogrammed with Sendai virus were used for WT comparisons, including Personal Genome Project cell line 1 (PGP1) and the NIH reference/control cell line KOLF2.1J^60^. PGP1 was primarily used for studies of ASO effect on healthy cells.

### Neurogenin 2 (NGN2) differentiation of hiPSCs

Modified from^61^, cortical neurons were differentiated via lentiviral transduction of a tetracycline-inducible NGN2 cassette, followed by doxycycline induction and neural induction by adding SMAD inhibitors SB431542 (Stem Cell Technologies 1001051), XAV939 (Tocris 374810), and LDN 193189 (Tocris 605310). NGN2-positive cells were selected using puromycin (Gibco). Differentiated neurons were plated onto laminin-treated coverslips (10 µg/ml) at 250k cells/well in 24-well plates and maintained in Neurobasal A media, B27, N2, Glutamax, and non-essential amino acids (Gibco), supplemented with 1:1000 BDNF (Millipore) and 1:5000 laminin (Gibco) at each media change^41^. Half-media changes were performed every 2–3 days. All experiments were repeated at least three times with independent differentiation batches of neurons, and the number of replicates is detailed after each experiment.

### Inhibitory neuron protocol

INs were differentiated using a modified iGABA protocol^24^ via PiggyBac plasmid transfection of a transgene containing the inducible transcription factors ASCL1 and DLX2 (PB-TO-ASCL1-DLX2) (Addgene 182307), followed by doxycycline induction (14 days) and treatment with SMAD inhibitors SB431542 (Stem Cell Technologies 1001051), XAV939 (Tocris 374810), and LDN 193189 (Tocris 605310). ASCL1/DLX2-positive cells were selected using puromycin (Gibco). Differentiated neurons were plated onto laminin-treated coverslips (10 µg/ml) at 250k cells/well in 24-well plates and maintained in Neurobasal A media, B27, N2, Glutamax, and non-essential amino acids (Gibco), supplemented with 1:5,000 laminin (Gibco) at each media change^41^. All differentiation experiments were repeated at least three times with independent batches of neurons, and the number of replicates is detailed after each experiment. Following differentiation, iGABA neurons were analyzed via IHC for GABA and neurons (MAP2), confirming >90% of neurons are GABA+.

### Primary neuronal cultures and neurite analysis

See “human sample collection” in the above methods for fetal collection parameters. Dissociation of primary human neuron cultures was modified from a previous method^27^. Immediately following tissue release from pathology, the neocortex was dissected from two human male samples (15 weeks and 23 weeks post-conception) according to the manufacturer’s protocol (MACS Neural Dissociation Kit, Miltenyi Biotec). Cortical neurons were plated onto laminin-treated coverslips (10 µg/ml) at 250k cells/well in 24-well plates and maintained in Neurobasal Plus media, B27 Plus, and 1:100 pen/strep (Thermo Fisher), supplemented with 1:5000 laminin at each media change. Half-media changes were performed every 2–3 days. Neurite lengths from the soma were traced and measured using ImageJ software.

### Antisense oligonucleotide design, production, and treatment

Gapmer ASOs designed to be complementary to the *KCNT1* gene (ASO) or control NT-ASO with comparable chemistry were synthesized by Microsynth. The *KCNT1*-specific ASO has a 5-10-5 gapmer design with a mixed phosphodiester/phosphorothioate backbone, GToToGoCCTTTGTAGCTGoAoGoGT, without any off-target binding sites in the human transcriptome. The NT-ASO shared a similar 5-10-5 gapmer design, also without any targets in the human transcriptome; see in silico targeting data for ASO selection and selectivity^1^. For NGN2 neurons, ASOs were delivered via gymnosis at 10 µM (free uptake after direct addition of ASOs to culture media). For NGN2 neurons, 10 µM treatment began at DIV 14 to DIV 21, followed by 5 µM ASO treatment from DIV 21 to DIV 28, when patching experiments began. As shown, this treatment significantly reduced *KCNT1* mRNA levels. ASO incubation was maintained via half-media changes for 10–14 days. In the case of primary human neurons, from DIV 21 to DIV 28, 10 µM was maintained instead of 5 µM to ensure robust *KCNT1* knockdown. For iGABA-neurons, 10 µM treatment began from DIV 28 to DIV 32, followed by 5 µM ASO treatment from DIV 32 to DIV 37, when analysis began.

### RNA isolation, cDNA synthesis, qRT-PCR

RNA isolation from iPSC-derived NGN2 neurons was performed using the RNeasy Plus Mini Kit (Qiagen) following the manufacturer’s protocol. The concentration and purity of the RNA samples were quantified using the Agilent Synergy LX Take3 plate. cDNA synthesis was performed using SuperScript IV (Invitrogen) with 100 ng of RNA per reaction. The qRT-PCR protocol was performed with SYBR Green 2× Master Mix (Applied Biosystems), with forward and reverse primers designed for the target gene sequences in purified water (Ambion) on MicroAmp EnduraPlate Optical 96-Well plates (Applied Biosystems). The efficiency of the primers was verified with melting curve analyzes, and the qPCR reaction was conducted with the QuantStudio 6 Pro (Applied Biosystems). Quantification of KCNT1 mRNA expression was performed by normalizing to the endogenous control gene expression and vehicle-treated cells using the delta-delta Ct method. ΔCt ASO or vehicle = CtKCNT1-ASO - CtB-GAPDH-ASO; ΔΔCt = ΔCt ASO – ΔCt vehicle; fold change = 2^(-ΔΔCt). The fold change, expressed as a percentage, served as a quantitative measurement of KCNT1 mRNA expression.

For Poison exon analysis, total RNA from fetal and postnatal brains (Biobank) was extracted using a standard TRIzol-chloroform (Invitrogen, 15596026, Fisher Chemical, BPC298500) protocol followed by the Zymo RNA Clean & Concentrate Kit 5 (Zymo Research, D4004). A sample of adult human cerebral cortex total RNA (Takara Bio, 636561, lot #2407059), pooled from three individuals aged 24–27, was purchased. Three µg of each RNA sample was used for reverse transcription with Maxima H Minus cDNA synthesis master mix (Thermo Fisher, M1662). The resulting cDNA was used for targeted PCR with *KCNT1-*specific primers. To detect alternative splicing events, a total of three pairs of primers spanning partially overlapping regions were used to cover the full-length *KCNT1* transcript: KCNT1_F2:GAGTTTGACGACGGCCAATG; KCNT1_F6:CTCCAACCTGGCCTTCATGT; KCNT1_F7:TTACGTGGTCATCCTGTGCC; KCNT1_R4:CGCATGAGGTCCTGGTCTTT;KCNT1_R7:CTGGCTCAGAGCTGTGTCTC;KCNT1_R8:GTTGGAGCCATTCTCTCGCT. The PCR products were purified using the QIAquick PCR purification kit (Qiagen, #28104). Samples were sent to Plasmidsaurus (San Francisco, CA) for long-read sequencing using the Premium PCR service. At least 250 high-quality reads mapping specifically to *KCNT1* were obtained per sample. We followed a previously described publication for a definition of poison exons in targeted long-read sequencing, using in silico translation to confirm the introduction of a premature termination codon into the reading frame^62^. For each poison exon, the percentage spliced-in was calculated as the percent of reads that included any part of the poison exon region divided by the reads that covered the adjacent canonical exons.

### Fluorescent immunohistochemistry

NGN2, primary human, and iGABA-neurons were analyzed with immunocytochemical staining to confirm cell types and distributions. For immunocytochemical characterization, the cells were first fixed with 4% PFA for 8–20 min at 4 °C. After washing with PBS, non-specific staining was blocked with 10% BSA-PBS-Donkey Serum − 0.3% Triton X-100, and primary antibodies were diluted into 10% BSA-PBS-0.1% Triton X-100 and incubated overnight at 4 °C. See the key resource table (Table S7) for primary antibodies used and their concentrations. After PBS washes, cells were incubated with secondary antibodies (1:500) for 1 h at room temperature and washed three times. On the third wash, nuclei were stained with DAPI (Thermo Fisher Scientific) for 5 min at room temperature. Coverslips were mounted onto charged SuperFrost Plus slides (Fisher). Fluorescent imaging was performed using an Axio Imager M2 microscope (Zeiss, Germany) or a Nikon S10 microscope. The cells were imaged with a 40X objective. Image processing and analysis were completed in ImageJ.

### Patch-clamp of iPSC-derived NGN2 and primary human neurons

Patch-clamp recordings were performed on DIV 26–32. NGN2 neurons were visualized using an upright Olympus BX51 microscope with epifluorescence. Current- and voltage-clamp data were recorded and analyzed with SutterPatch IPA (Sutter Instruments, Sunnyvale, CA). Glass pipettes were formed from borosilicate glass (3–5 MΩ) on a Sutter Puller and filled with a no-sodium intracellular solution to isolate sodium-activated currents: 97.5 mM K-Gluc, 32.5 mM KCl, 10 mM HEPES, 1 mM EGTA, and 2 mM MgCl_2_. The internal solution was adjusted to 295 mOsm and a pH of 7.4 with KOH. The internal solution also contained Alexa-Fluor 488 nm dye to visualize neuron morphology (1 mM, Thermo Fisher). For patching of NGN2 neurons, the extracellular recording solution, ACSF, contained the following: 125 mM NaCl, 2.5 mM KCl, 2 mM CaCl_2_, 1 mM MgCl_2_, 1.25 mM NaH_2_PO_4_, 26 mM NaHCO_3_, 15 mM glucose, 1 mM myo-inositol, 2 mM Na-pyruvate, and 0.4 mM ascorbic acid, pH 7.4 (adjusted with NaOH), 300 mOsm. ACSF solution was continuously oxygenated during experiments with 95% O_2_ and 5% CO_2_ to maintain pH 7.4. For patching of fetal neurons, the extracellular recording solution, ACSF, contained the following: 140 mM NaCl, 5.4 mM KCl, 1 mM CaCl_2_, 1 mM MgCl_2_, 10 mM HEPES, and 10 mM glucose, pH 7.4 with NaOH, 300 mOsm. The sodium substitution external solution (NMDG-ACSF) contained 140 mM NMDG, 5.4 mM KCl, 1 mM CaCl_2_, 1 mM MgCl_2_, 10 mM HEPES, and 10 mM glucose. For the number of cells patched per genotype, see Tables S1–S4.

For voltage-clamp recordings on iPSC-derived neurons, 500 ms voltage pulses beginning at –80 mV and ending at +50 mV (10 mV increments) were used to elicit currents. The outward steady-state current was measured in response to each voltage pulse as the mean of the last 10 ms of the response. For primary cortical neurons, the voltage-clamp routine was slightly modified to begin at –90 mV (instead of –80 mV) and progress in 5 mV increments up to +60 mV to better determine the half-activation potential of KCNT1.

To study the first AP kinetics in current-clamp (rheobase), 40 ms current pulses increasing in 2 pA increments were injected into the neuron until the first AP was achieved. The first AP threshold was calculated by locating the point during depolarization where the first derivative of the voltage vs. time plot was minimized within a 15 ms window before the peak. AP amplitude was calculated as the potential difference between the threshold and the peak. The AHP was defined as the potential difference between the threshold and the voltage minimum within a 10–200 ms window after the peak^16^. For the identification of fAHP, we located the point at which the derivative was closest to zero within an analysis window of 5–20 ms after the peak (time of detection for fAHP and mAHP reported in Table S1). The full width of the AP was defined as the horizontal (temporal) distance between the upstroke and downstroke of the AP at the threshold potential. AP latency was defined as the time delay between the beginning of the current pulse and the time at which the AP threshold was reached. The AHP area was calculated using a trapezoidal Riemann sum with respect to the holding potential (*V*_hold_ = –60 mV) for 200 ms following the AP peak using a step size of ∆*t* = 0.1 ms. sAHP area after prolonged current injection (500 ms), resulting in spiking bursts, was calculated via Riemann sum over 500 ms following the end of the burst. To quantify stimulus-evoked action potential rundown, during current 500 ms pulses, the change in action potential peak amplitude (Δ Amplitude) from first-to-second AP was quantified, with numbers closer to 0 representing limited rundown and more negative Δ Amplitude is more run down (poorer action potential recovery).

### Human brain tissue preparation and spatial mRNA in situ hybridization

See “human sample collection” in the above methods for fetal collection parameters. Brain tissue preparation and spatial mRNA in situ hybridization were performed as previously described by Smith et al.^21,27^. Briefly, following fixation (4% PFA) and cryoprotection (30% sucrose), brains were frozen using isopentane on dry ice. Samples were sectioned at 8–20 μm thickness using a Leica Cryostat, mounted immediately onto warm charged SuperFrost Plus slides (Fisher), and stored at –80 °C. Fluorescent imaging was performed on selected cortical areas using an Axio Imager M2 microscope (Zeiss, Germany) or a Nikon S10 microscope. The cells were imaged with a 40X objective. We followed the manufacturer’s standard protocol for multiplex fluorescent in situ hybridization (Multiplex Version 2 kit, Advanced Cell Diagnostics). See the supplemental materials for in situ probe ACD catalog numbers. Bright-field and fluorescent images were background-corrected using Zen Blue software for center intensity illumination and stitched together. Analysis was performed by quantifying KCNT1 puncta in cell types defined by the presence of at least 10 puncta of specific markers (Fig. S3). We followed manufacturer’s protocol for Click-iT™ Plus TUNEL Assay Kits for In Situ Apoptosis Detection (Thermo Fisher, C10617).

### Bulk human cortex gene expression analysis

The Allen Brain Atlas publishes a rich dataset of cortical gene expression across cortical brain regions from age 8 weeks post-conception to adulthood^20^. BrainSpan data analysis of KCNT1 (chr1:116,915,289–116,952,883, GRCh37/hg19) was performed. RNA-seq expression, measured in RPKM (reads per kilobase exon per million mapped reads), was obtained from the BrainSpan project data and summarized to Gencode v10 exons for all annotated neocortical tissues aged 12 weeks post-conception to 36 years. Brain regions for Fig. 3 include the dorsolateral prefrontal cortex, ventrolateral prefrontal cortex, anterior (rostral) cingulate (medial prefrontal) cortex, orbital frontal cortex, primary motor-sensory cortex, parietal neocortex, posterior (caudal) superior temporal cortex (area 22c), inferolateral temporal cortex (area 20), occipital neocortex, temporal neocortex, primary motor cortex (area 4), primary somatosensory cortex (area S1, areas 3, 1, 2), posteroventral (inferior) parietal cortex, and primary auditory cortex (core).

### Statistical analyses

Statistical analyzes were performed using MATLAB and/or R. Depending on normality, parametric or non-parametric tests were conducted: two-way ANOVA followed by Tukey’s multiple comparisons test or Kruskal–Wallis test followed by Dunn’s multiple comparisons test, with *p*-values adjusted for multiple comparisons. For independent or within-group comparisons, unpaired *t*-tests with two-tailed *p*-values were used. Unless otherwise indicated, *p*-values are presented as follows: ^*^p < 0.05, ^**^p < 0.01, ^***^p < 0.001, and ^****^p < 0.0001. Figure legends include the details of the statistical methods used for the analysis of each dataset. Boxplots show the median values with interquartile range (IQR). Table values are mean ± SEM, and *p*-values are reported between vehicle and ASO-treated conditions unless otherwise noted.

## Supplementary information

Supplementary Information

Transparent Peer Review file

## Source data

Source Data

## Data Availability

Relevant source data are provided within this paper. RNA-sequencing data is available on NCBI Gene Expression Omnibus (GEO) and are available under GEO Series accession GSE297948. The reference resources used in this study include the Genome Reference Consortium Human Build 38 (GRCh38) assembly. See “Source Data” for data series. Source data are provided with this paper.

## Code availability

See Richard Smith Lab GitHub for Analysis code or access to data. https://github.com/Smith-Lab-Neuro.
