# Supplementary material for: RNA targeting therapy for a prenatally enriched potassium channel associated with severe childhood epilepsy and premature death
Source: Nat Commun. 2026 Apr 29;17:5864. doi: 10.1038/s41467-026-72334-7 (PMC13333874; doi:10.1038/s41467-026-72334-7)
Supplement: Supplementary file 1 — Supplementary Information [file 41467_2026_72334_MOESM1_ESM.pdf]

Title: RNA targeting therapy for a prenatally enriched potassium channel associated with severe childhood epilepsy and premature death

Authors: Sean R. Golinski[1], Karla Soriano[1], Alex C. Briegel[1], Madeline C. Burke[1], Sheng Tang[1], Gemma L. Carvill[1], Emma Sherrill[2], Claudia Lentucci[2], Timothy W. Yu[2], Tojo Nakayama[2], Ruilong Hu[1], Richard S. Smith[1]

Affiliations:

1. Northwestern University, Feinberg School of Medicine, Chicago, IL, 60611
2. Division of Genetics and Genomics, Boston Children's Hospital, Boston, MA, 02115

Corresponding Author: Richard S. Smith, PhD, Northwestern University

Telephone: (312) 503-2576

*Correspondence and material requests should be addressed to: Richard.smith@northwestern.edu

Supplemental Figures

Figure S1

KCNT1-p.R474H ENs display large AHPs sensitive to Ca^{2+}-activated BK and SK channel blockers

Figure S2

ASO-treated control ENs exhibit increased AHP amplitude, with small unaffected outward K+ current

Figure S3

ASO-treated DEE14 ENs do not show activation of apoptosis or cell death pathways

Figure S4

ASO-treated WT and KCNT1-p.R474H NGN2 neurons exhibit improved dynamic range of firing sensitive to various K channel blockers.

Figure S5

Prenatal emergence of Slack currents in mid-gestation in primary human neurons

Figure S6

ASO-treated fetal human neurons are sensitive to KCNT1 knockdown

Figure S7

INs from KCNT1-p.R474H patient display altered AP kinetics compared to control

Supplemental Tables

Table S1

Electrophysiology analysis and statistics of ASO-treated KCNT1-p.R474H ENs

Table S2

Electrophysiology values and statistics for excitability and sAHP of ASO-treated KCNT1-p.R474H ENs

Table S3

Electrophysiology analysis and statistics of K_{Na} currents in primary fetal neurons, including ASO knockdown

Table S4

Electrophysiology values and statistics for ASO-treated KCNT1-p.R474H INs

Table S5

RNAseq data, AP genes analyzed for ASO-treated ENs vs. control ENs

Table S6

RNAseq data, All differentially expressed genes for ASO-treated ENs vs. control ENs

Table S7

KEY RESOURCE TABLE

Supplemental Figures

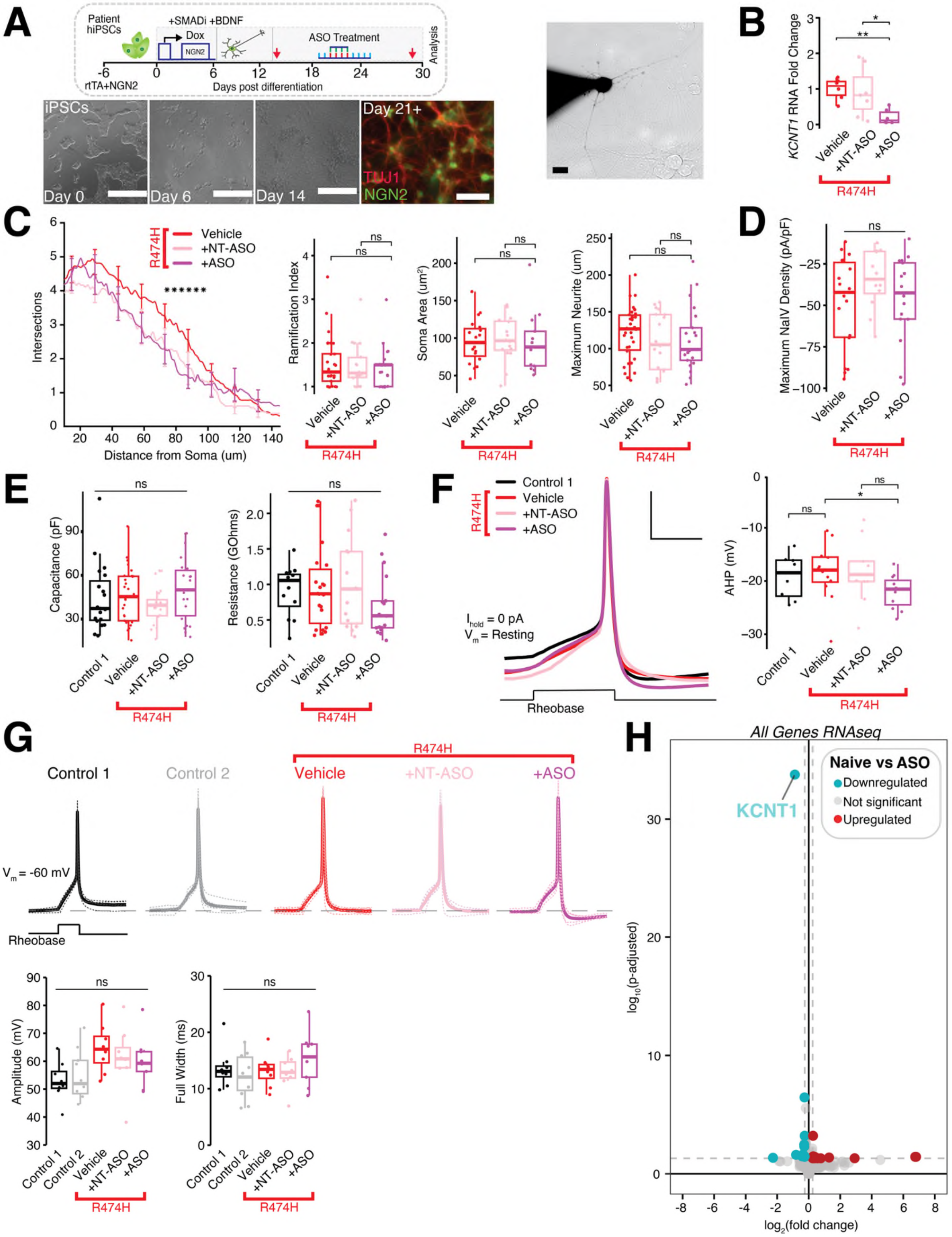

Figure S1. KCNT1-p.R474H ENs display large AHPs sensitive to Ca^{2+}-activated BK and SK channel blockers

A. *Top*, schematic of NGN2 differentiation of iPSCs to neurons from an individual with the *KCNT1*-p.R474H variant**.** *Bottom*, representative images at key steps in the NGN2-directed differentiation protocol and immunofluorescence image showing NGN2 labeling and a neuron-specific marker (TUJ1). Scale bars: Day 0, 750 µm; Days 6 and 14, 150 µm; Day 21, scale bar 50 µm. *Right*, representative image of patched neuron loaded with Alexa-Fluor 488 dye (1 µM). **B.** Quantitative RT-PCR analyses of *KCNT1* in p.R474H NGN2 neurons following treatment with vehicle, ASO, or NT-ASO. Fold changes in KCNT1 expression levels were normalized to GAPDH expression; Wilcoxon test, $p = 0.004$ between vehicle and ASO. **C.** *Left*, Sholl analysis of p.R474H NGN2 neurons ($p < 0.05$ for distances from 80–100 µm from cell soma). *Right*, corresponding ramification index, soma surface area, and longest primary neurite length. No significant differences were observed in ramification index, soma area, or longest primary neurite length. **D.** Capacitance-adjusted values for peak inward sodium current reveal that differences in sodium influx were not the mechanism causing reduced K^+ current in ASO-treated cells. **E.** Analysis of capacitance and input resistance of neurons during patch-clamp analysis, with no significant differences observed in capacitance or resistance ($p = 0.88$, $p = 0.8$, respectively). **F.** *Left*, overlaid average of first action potential for each condition with neurons recorded at resting potential ($I_{hold} = 0$ pA). AHP is increased in p.R474H neurons treated with ASO, compared to vehicle ($p = 0.032$). Scale bars: 20 mV and 50 ms. **G.** *Top*, representative current-clamp recordings of a minimally stimulated first action potential for each condition and overlaid average, (including control lines KOLF [Control 2] and PGP1-NGN2 [Control 1] neurons). Dashed line indicates neurons held at –60 mV. *Bottom*, analysis of AP properties of KOLF, PGP1, and p.R474H NGN2 neurons. See Table S1 for complete values. **H.** Volcano plot of RNA-seq differentially expressed genes for ENs treated with ASO vs. control. Dotted significance line p-adjust < 0.05. See Tables S5 and S6 for RNA-seq statistical values.

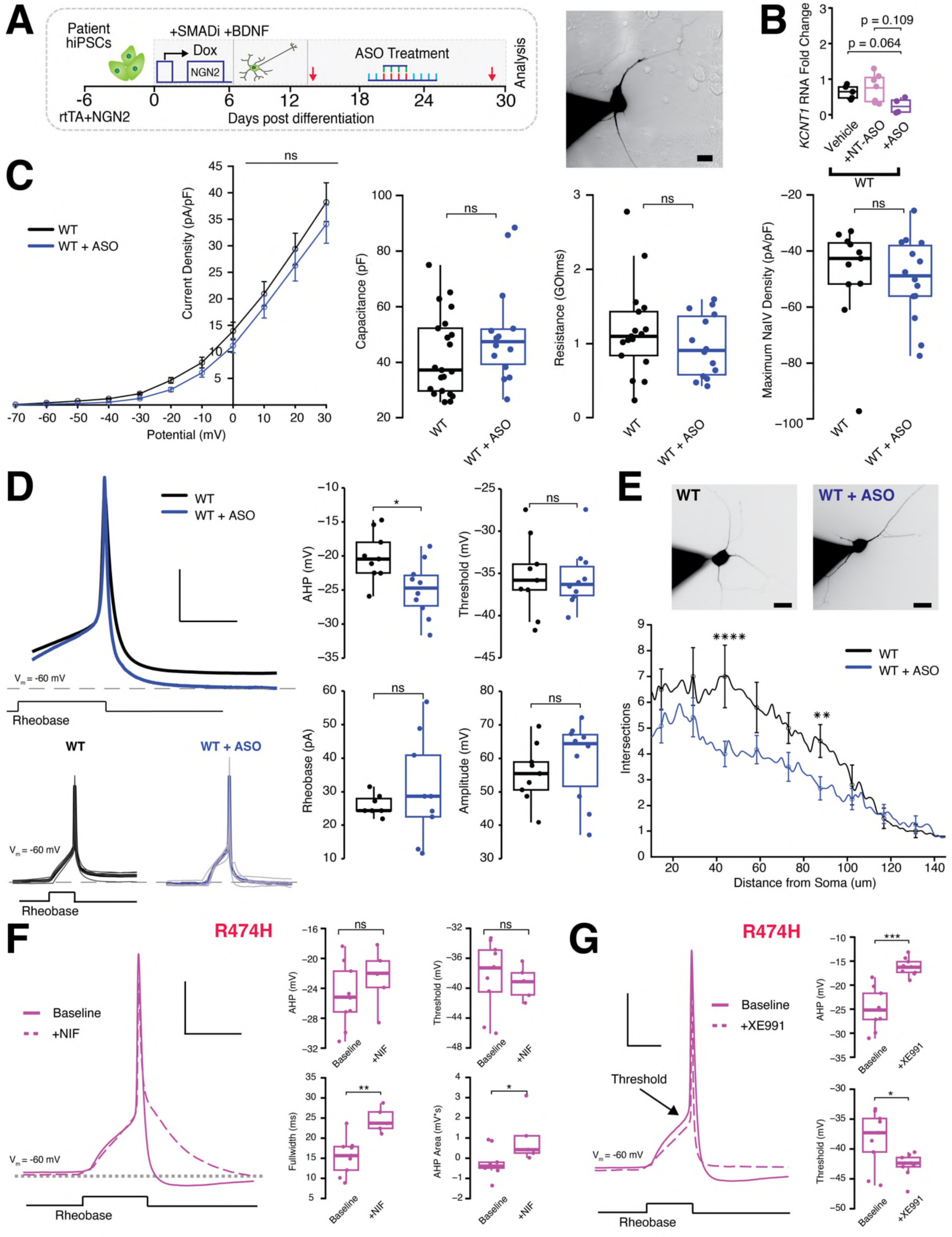

Figure S2. ASO-treated control ENs exhibit unaffected outward K+ current, with increased AHP amplitude and normal excitability curve

A. *Left*, schematic of NGN2 differentiation of iPSCs to neurons from healthy control line PGP1 (WT)**;** Day 0 iPSCs, Day 0–6 doxycycline treatment, Day 14 ASO treatment begins, Day 28 patch-clamp assays. *Right*, representative image of patched WT neuron loaded with Alexa-Fluor 488 dye (1 µM). **B.** Quantitative RT-PCR analyses of *KCNT1* in WT NGN2 neurons following treatment with vehicle, ASO, or NT-ASO. Fold changes in KCNT1 expression levels were normalized to GAPDH expression; Wilcoxon test, $p = 0.064$. **C.** *Left*, IV curve of outward steady-state K^+ current density in WT and WT+ASO-treated neurons. No significant reduction in current was observed in response to ASO treatment for WT neurons. *Right*, average capacitance, resistance, and peak sodium current density for WT and WT+ASO neurons reveal no differences between treatments. See Table S1 for complete values. **D.** *Left*, average first action potential for WT and WT+ASO neurons held at –60 mV (zoomed in; scale bars: 20 mV and 50 ms), and representative recordings overlaid with average. *Right*, ASO treatment increased AHP following a single action potential in WT neurons but did not affect threshold potential, rheobase current, or amplitude. **E.** Representative dye-loaded neurons during patch recordings and Sholl analysis of WT and WT+ASO neurons. Scale bars 20 µm. Small complexity differences observed in ASO-treated neurons at 40–100 µm from the soma ($p < 0.05$). **F.** Average action potential of ASO-treated p.R474H neurons following bath perfusion with calcium channel blocker nifedipine (Nif, 100 µM, dashed) shows disrupted AHP kinetics (measured as AHP area) compared to baseline (solid line), including a significantly greater full width ($p = 0.0025$). However, AHP returned to normal levels within the 200 ms time window following AP peak. Threshold potential was not affected by Nif application. Scale bars: 20 mV and 50 ms. **G.** Average action potential of ASO-treated p.R474H neurons with bath-perfused KCNQ antagonist XE991 (100 µM, dashed) compared to baseline (solid line). *Right*, XE991 perfusion resulted in a significant reduction of AHP and hyperpolarized the AP threshold ($p < 0.001$, $p = 0.032$). Note that the reduction in AHP is partly due to the hyperpolarized threshold from which the AHP is measured. See Table S1 for complete values.

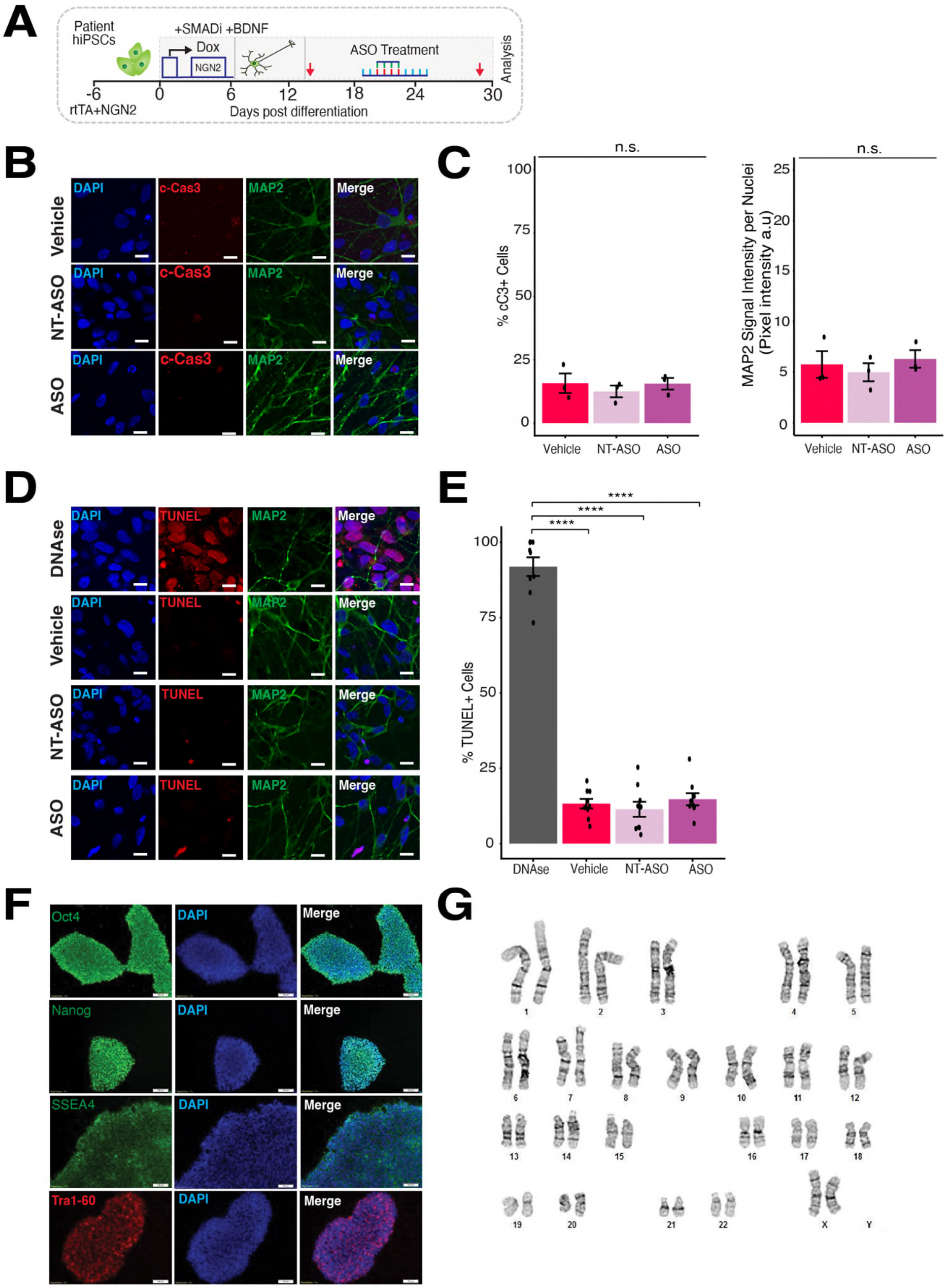

Figure S3. ASO-treated DEE14 ENs do not show activation of apoptosis or cell death pathways

A. Schematic of NGN2 differentiation of iPSCs to neurons from healthy control line PGP1 (WT)**;** Day 0 iPSCs, Day 0–6 doxycycline treatment, Day 14 ASO treatment begins, Day 28 fixation and immunohistochemistry. **B.** Analysis of *KCNT1,* cell type marker MAP2, and apoptosis marker cleaved caspase-3 in differentiated NGN2 neurons. Scale bar 10 µm. **C.** Percentage of c-Cas3$^+$ cells from B. Each bar represents the average percentage of c-Cas3$^+$ cells on three coverslips per condition. $F = 0.231$, $p > 0.05$. Data are presented as mean +/- standard error. MAP2 signal intensity per nucleus: each bar represents the average MAP2 signal intensity per nucleus on three coverslips per condition. $F = 0.578$, $p > 0.05$. Data are presented as mean +/- standard error. **D.** Analysis of *KCNT1,* cell type marker MAP2, and apoptosis via TUNEL in differentiated NGN2 neurons. **E.** Percentage of TUNEL$^+$ cells from D. Each bar represents the average percentage of TUNEL+ cells on three coverslips per condition. $F = 275.3$, ****p = 1.60197E-08, ****p = 2.42515E-07, ****p = 6.67809E-09. Data are presented as mean +/- standard error. **F.** Immunocytochemistry identifying markers of pluripotent stem cells in the KCNT1-p.R474H hIPSCs. Scale bar 100 µm. **G.** Karyotype performed by WiCell for the KCNT1-p.R474H hIPSC cell line, normal karyotype reported (Report # 076824).

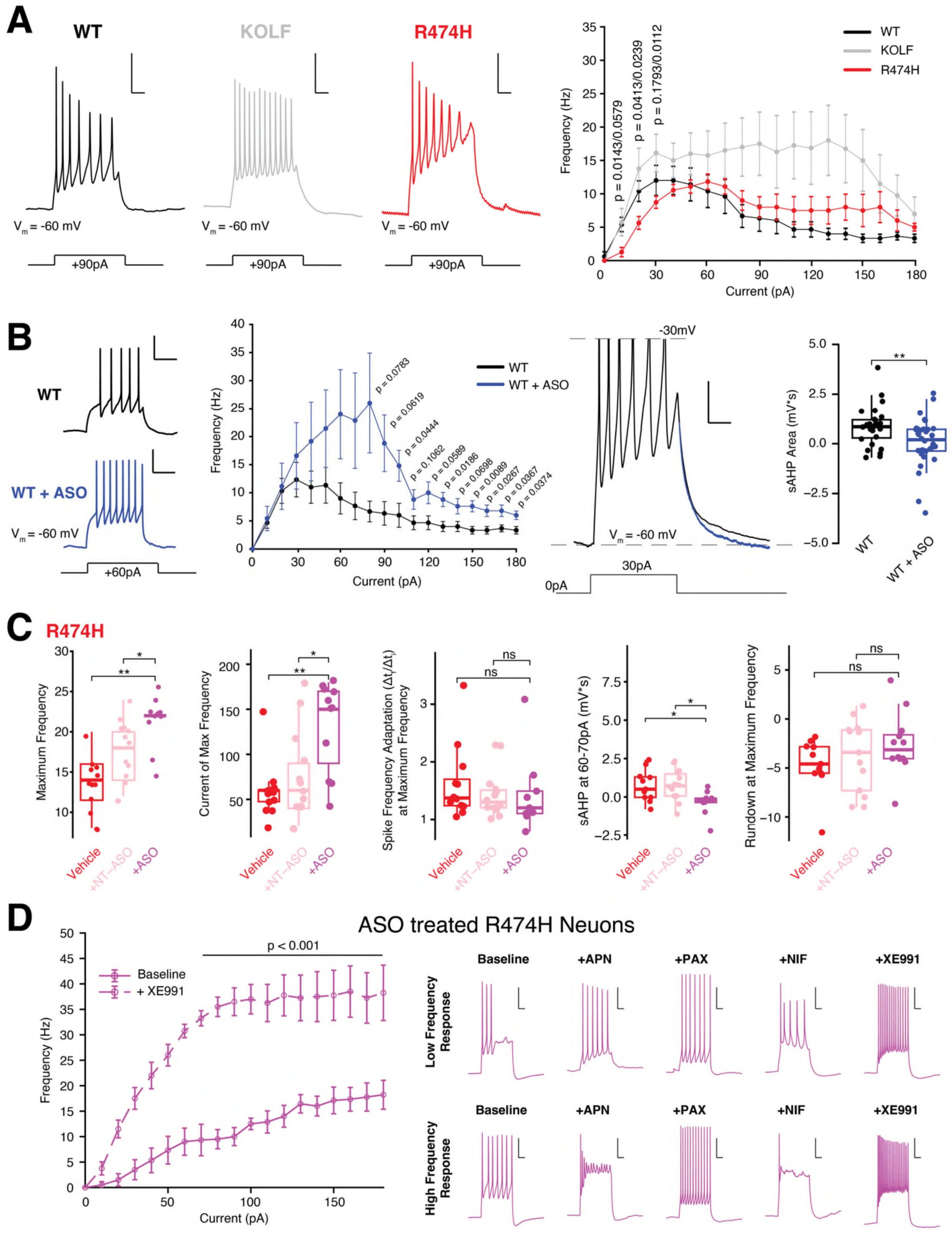

Figure S4. ASO-treated WT and KCNT1-p.R474H NGN2 neurons exhibit improved dynamic range of firing sensitive to various K channel blockers.

A. *Left*, representative spiking traces of KCNT1-p.R474H and two control lines, WT and KOLF, at +90 pA stimulus. Scale bars: 20 mV and 100 ms. *Right*, frequency curve for each condition from –10 to +180 pA in 10 pA steps. P-values are formatted as R474H vs. WT / R474H vs. KOLF. **B.** *Left*, representative spiking traces of WT and WT+ASO conditions at +60 pA. Scale bars: 20 mV and 200 ms. Frequency curve of WT and WT+ASO conditions shows ASO-induced excitability at later current injections, similar to the effect observed in patient ENs. *Right*, ASO increased sAHP following AP burst in WT neurons for frequencies from 6–10 Hz. Frequencies above 10 Hz were excluded due to saturation. Scale bars: 10 mV and 200 ms. **C.** Additional spiking properties of patient ENs (vehicle, NT-ASO, and ASO-treated). **D.** *Left***,** frequency curve of ASO-treated patient neurons with and without bath perfusion of XE991. XE991 dramatically increased excitability at all current injections. *Right*, representative spiking traces of ASO-treated patient ENs at low- and high-frequency responses when bath perfused with various channel blockers (APN, PAX, NIF, XE991). Scale bars: 20 mV and 100 ms.

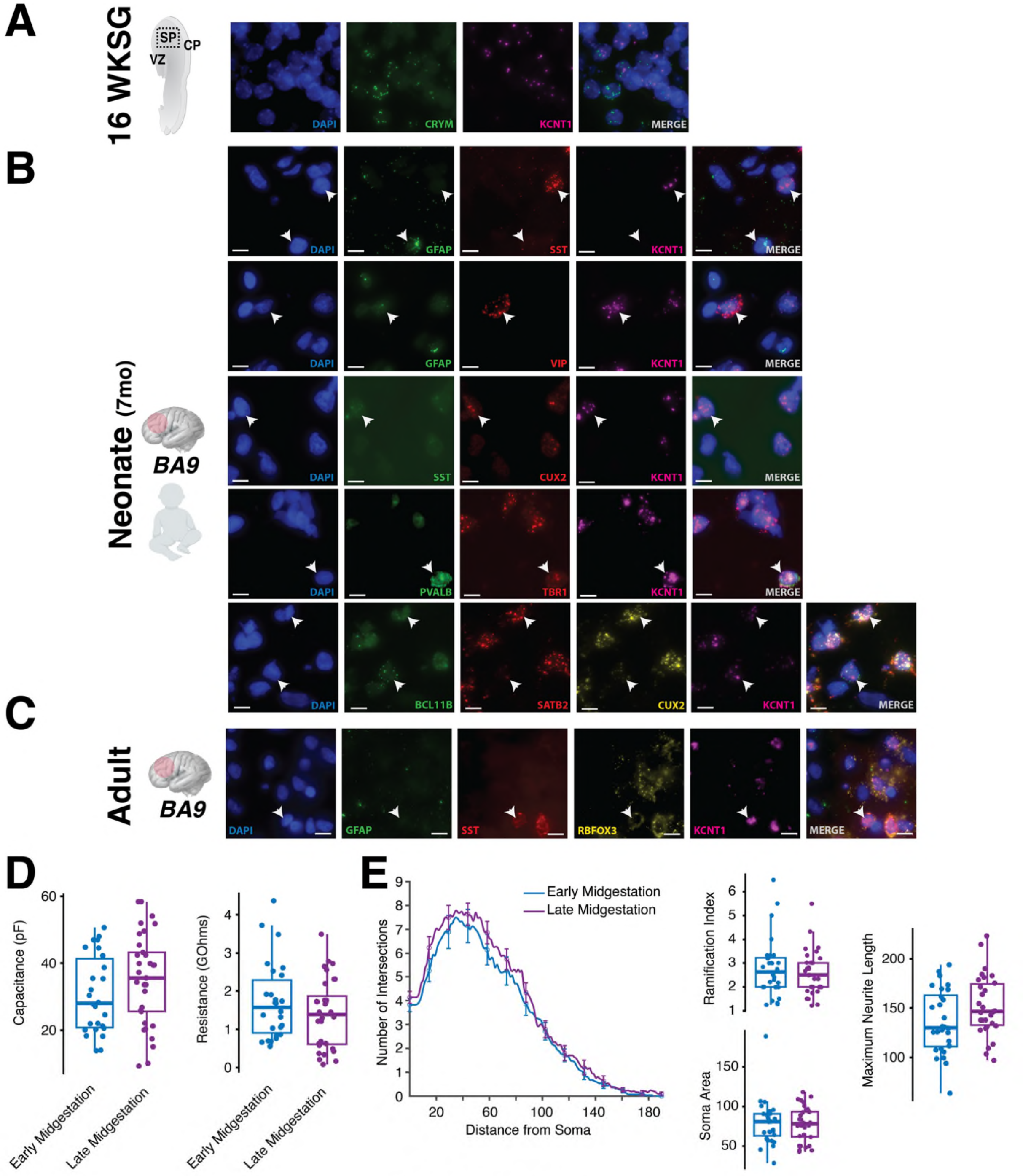

F

Features of Poison Exons

Poison exon	Coordinates (hg38)	Length (nt)
2N	chr9:135715262-135715438	177
4N	chr9:135752379-135752457	79

In silico translations

Canonical KCNT1 (first 5 exons only):
MPLPDGARTPGGVCREARGGGYTNRTFEFDDGQCAPRRPCAGDGALLDTAGFKMSDLDSEVLPLPPRY
RFRDLLLGDPSFQNDDRVQVEFYVNENTFKERLKLFFIKNQRSSLRIRLFNFSLKLLTCLLYIVRVLLDDP
ALGIGCWGCPKQNYSFNDSSSEINWAPIL

KCNT1 with 2N:
MPLPDGARTPGGVCREARGGGYTNRTFEFDDGQCAPRRPCAGDGALLDTAGFKMSDLDSEVLPLPPRY
RFRDLLLGDPSFQNDDS*

KCNT1 with 4N:
MPLPDGARTPGGVCREARGGGYTNRTFEFDDGQCAPRRPCAGDGALLDTAGFKMSDLDSEVLPLPPRY
RFRDLLLGDPSFQNDDRVQVEFYVNENTFKERLKLFFIKNQRSSLRIRLFNFSLKLLTCLLYIVRVLLDDP
ALGIGWSEEGRTH*

Underlined = contribution from poison exon

* = STOP codon

Figure S5. Prenatal emergence of Slack currents in mid-gestation in primary human neurons

A. *KCNT1* RNA *in situ* hybridization of a 16-week gestation (WKSG) coronal brain section from the perisylvian region demonstrates enrichment in the cortical plate (CP) and subplate marker CRYM. CP, cortical plate; VZ, ventricular zone. **B.** Analysis of *KCNT1* and cell type marker expression in the neonatal neocortex (7-month-old), including a pyramidal neuron marker (TBR1), interneuron markers (PV, VIP, and SST), and a glial marker (GFAP). **C.** Analysis of KCNT1 and cell type marker expression in adult neocortex (Brodmann area, BA9), with cell type-specific markers, including RBFOX3 (neurons), SST (interneurons), and GFAP (glia). **D.** Analysis of capacitance and input resistance of early vs. late mid-gestation primary neurons; no significant differences were observed ($p = 0.208$, $p = 0.244$, respectively). **E.** *Left*, Sholl analysis of primary neurons at early and late mid-gestational timepoints. *Right*, corresponding ramification index, soma surface area, and longest primary neurite length. No significant differences were detected in complexity, ramification index, soma area, or longest primary neurite length, suggesting that the cell morphology did not change while in culture (see Table S3 for values). **F.** *Left*, poison exon genomic locations. *Right*, *in silico* translation of poison exons inserted in-frame into the canonical *KCNT1* transcript shows introduction of premature termination codons.

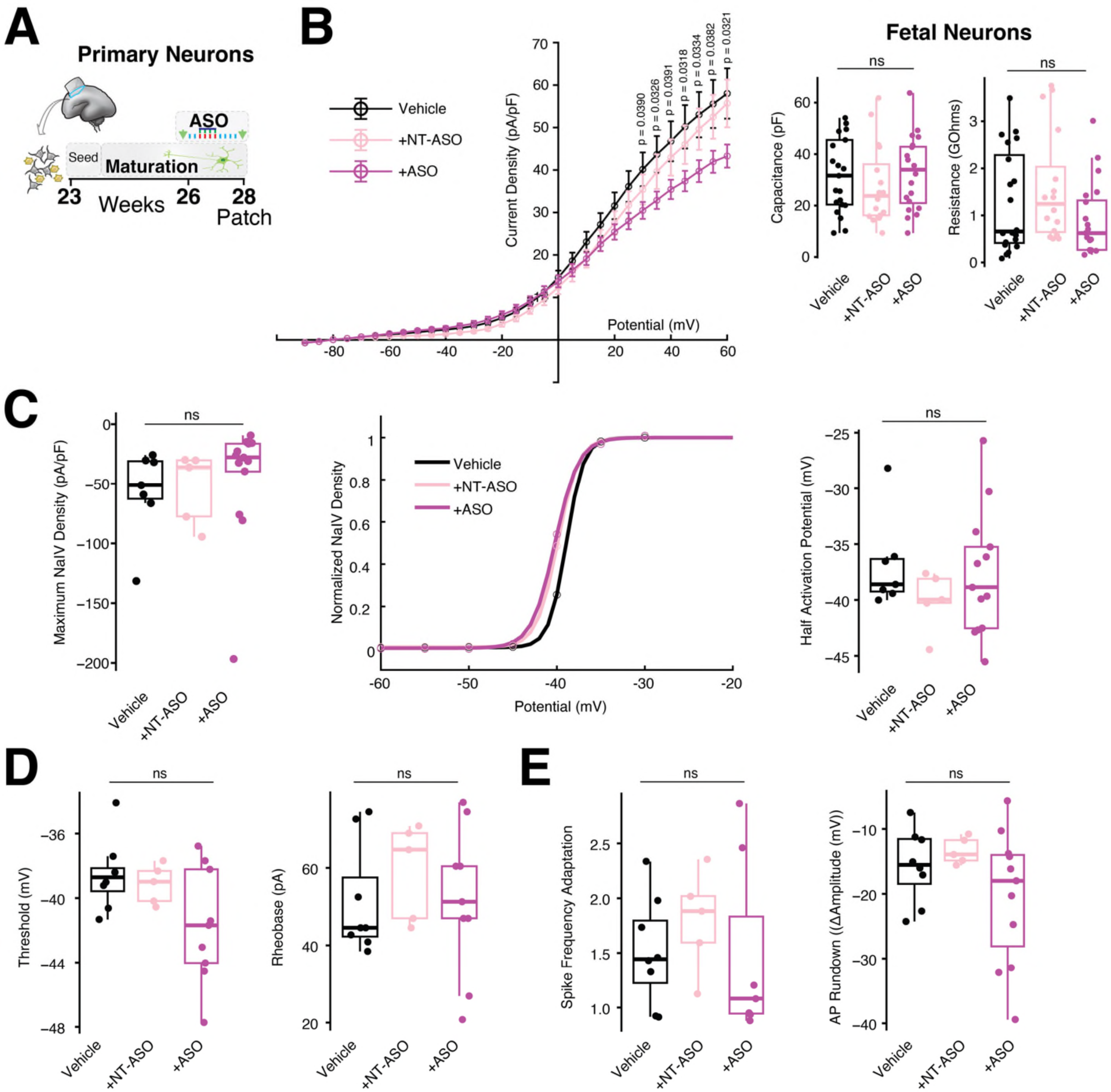

A
Primary Neurons
Seed
Maturation
ASO
23
Weeks
26
28
Patch
B
Vehicle
+NT-ASO
+ASO
Current Density (pA/pF)
Potential (mV)
p = 0.0390
p = 0.0326
p = 0.0391
p = 0.0318
p = 0.0334
p = 0.0382
p = 0.0321
Fetal Neurons
Capacitance (pF)
Resistance (GOhms)
ns
C
Maximum NaIV Density (pA/pF)
Normalized NaIV Density
Half Activation Potential (mV)
D
Threshold (mV)
Rheobase (pA)
E
Spike Frequency Adaptation
AP Rundown ((ΔAmplitude (mV))

Figure S6. ASO knockdown of K_{Na}1.1 (*KCNT1*) in mid-gestation primary human neurons

A. *Top*, schematic of primary neuron isolation from mid-gestation human cortex and maturation timeline with ASO treatment**. B.** Primary fetal neurons at 28 PCW equivalent isolated from a 23 PCW sample exhibited outward K^+ current densities sensitive to ASO knockdown (10 µM, 14 days). ASO treatment did not significantly alter fetal neuron capacitance or input resistance during patch experiments. **C.** ASO treatment did not affect maximum Na^+ current magnitude or half activation potential in fetal neurons (see Table S4). **D,E.** Primary fetal neurons showed comparable average thresholds, rheobase, spike frequency adaptation, and rundown across all conditions (n.s., see Table S3).

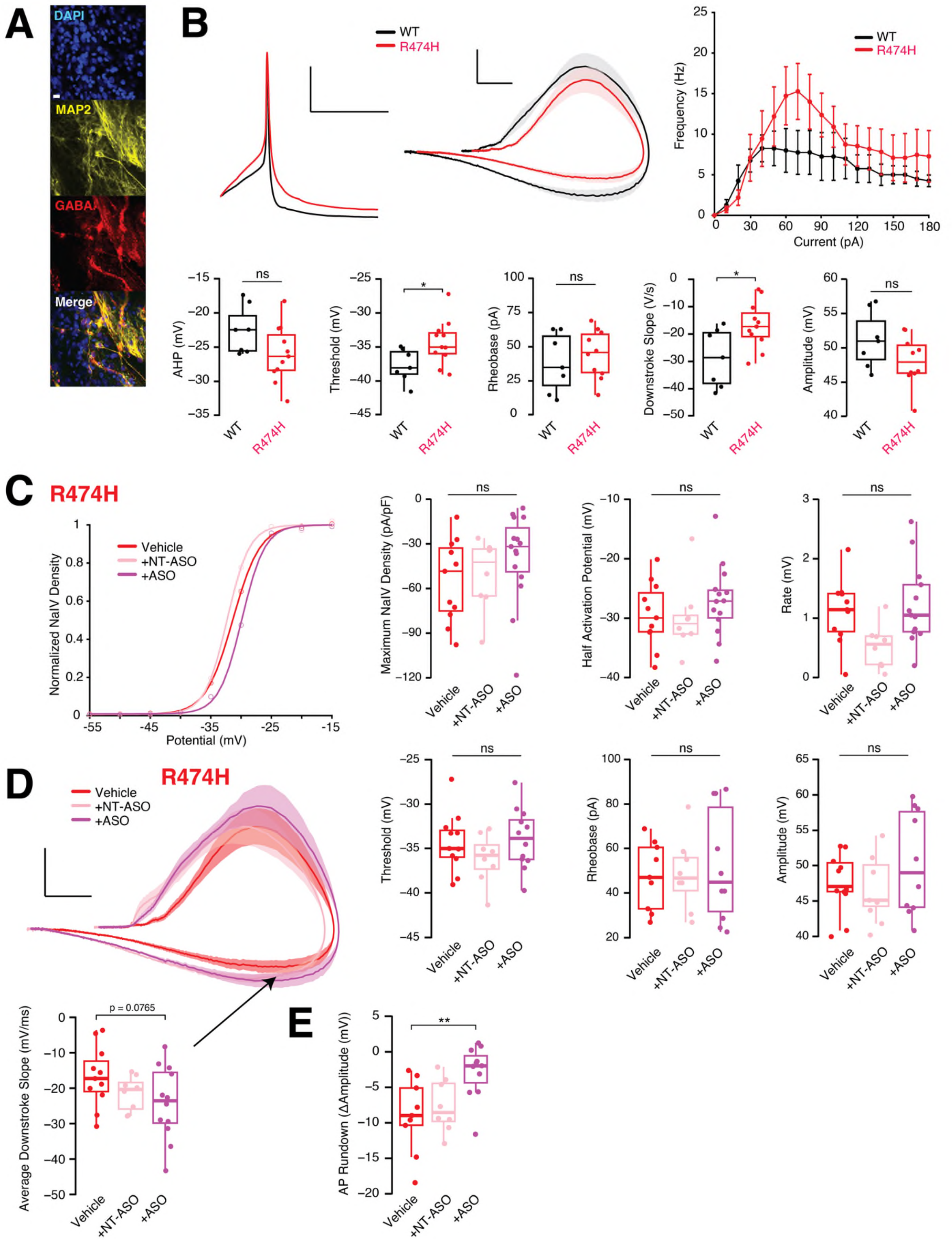

Figure S7. INs from KCNT1-p.R474H patient display altered AP kinetics compared to control

A. Immunofluorescence images from a replicate differentiation using a 35-day iGABA differentiation protocol showing antibody labeling of neuronal-specific marker microtubule-associated protein 2 (MAP2) and GABA marker anti-GABA. Scale bar 10µm. **B.** *Top left*, average AP and phase plane of WT and patient iGABA INs. Scale bars: 20 mV, 100 ms, and 20 V/s and 20 mV, respectively. *Top right*, frequency curves of WT (PGP1) and R474H iGABA INs show increased but not significantly different excitability in patient line from 60–90 pA current injections. *Bottom*, AP properties of patient iGABA INs compared to WT. As shown in the average AP and phase plane, patient neurons exhibit a depolarized threshold potential and slower downstroke. **C.** Sodium channel activation properties of patient iGABA INs indicate ASO treatments had no effect on sodium channel function. **D,E.** Phase plane and AP properties of patient iGABA INs in response to ASO treatments, including rundown. Only AP downstroke displayed a mild difference. Scale bars: 20 mV, 100 ms and 20 V/s and 20 mV, respectively.

	WT	Vehicle	+NT-ASO	+ASO	p-value	Figure
Observations	$N = 26$	$N = 28$	$N = 18$	$N = 24$		
Max I_K (pA/pF)	43.98 ± 3.45	54.43 ± 3.70	56.35 ± 4.14	37.45 ± 3.49	$p = 0.002$ **	1-B
Max I_{Na} (pA/pF)	N/A	-48.61 ± 6.61	-34.46 ± 5.14	-45.46 ± 6.03	$p = 0.988$	S1-D
Capacitance (pF)	44.66 ± 5.29	46.19 ± 3.41	39.67 ± 2.88	50.61 ± 4.59	$p = 0.565$	S1-E
Resistance (GΩ)	0.92 ± 0.10	0.96 ± 0.13	1.01 ± 0.16	0.69 ± 0.11	$p = 0.218$	S1-E

	Vehicle	+NT-ASO	+ASO	p-value	Figure
Observations	$N = 8$	$N = 8$	$N = 9$		
AHP (mV)	-18.19 ± 0.94	-18.90 ± 0.85	-25.05 ± 1.43	$p = 0.001$ **	1-D
Threshold (mV)	-38.76 ± 0.91	-40.36 ± 1.11	-38.35 ± 1.60	$p = 0.831$	1-D
Rheobase (pA)	28.32 ± 4.02	31.73 ± 4.80	46.04 ± 5.66	$p = 0.025$ *	1-D
AHP at RMP (mV)	-18.38 ± 1.57	-18.33 ± 2.06	-22.03 ± 0.96	$p = 0.032$ *	S1-F
Amplitude (mV)	64.72 ± 3.15	60.90 ± 4.14	59.93 ± 2.92	$p = 0.281$	S1-G
Full Width (ms)	13.24 ± 1.06	12.84 ± 1.05	15.40 ± 1.54	$p = 0.279$	S1-G
Latency (ms)	39.36 ± 1.96	35.83 ± 3.63	34.99 ± 2.77	$p = 0.227$	N/A
fAHP Time (ms)	16.49 ± 1.36	19.44 ± 0.48	18.92 ± 0.70	$p = 0.120$	N/A
mAHP Time (ms)	106.80 ± 23.09	106.69 ± 26.77	84.60 ± 22.04	$p = 0.498$	N/A

Vehicle	ACSF	+PAX	+APN	p-value	Figure
Observations	$N = 8$	$N = 11$	$N = 10$		
AHP (mV)	-18.19 ± 0.94	-18.62 ± 0.87	-19.21 ± 1.10	$p = 0.778$, $p = 0.515$	1-E
Threshold (mV)	-38.76 ± 0.91	-40.02 ± 1.03	-38.62 ± 0.77	$p = 0.351$, $p = 0.965$	1-E

+NT-ASO	ACSF	+PAX	+APN	p-value	Figure
Observations	$N = 8$	$N = 9$	$N = 8$		
AHP (mV)	-18.90 ± 0.85	-18.14 ± 1.27	-18.81 ± 1.45	$p = 0.423$, $p = 0.645$	1-E
Threshold (mV)	-40.36 ± 1.11	-39.32 ± 0.90	-38.06 ± 0.53	$p = 0.541$, $p = 0.161$	1-E

+ASO	ACSF	+PAX	+APN	p-value	Figure
Observations	$N = 9$	$N = 13$	$N = 12$		
AHP (mV)	-25.05 ± 1.43	-20.36 ± 0.71	-18.11 ± 1.13	$p = 0.022$, $p = 0.005$ */**	1-E
Threshold (mV)	-38.35 ± 1.60	-39.52 ± 0.56	-39.64 ± 1.03	$p = 0.262$, $p = 0.422$	1-E

	Vehicle	+NT-ASO	+ASO	p-value	Figure
Observations	$N = 6$	$N = 7$	$N = 6$		
Fold Change ($\log_2$)	N/A	0.91 ± 0.27	0.22 ± 0.09	$p = 0.004$, $p = 0.022$ **/*	S1-B

	Vehicle	+NT-ASO	+ASO	p-value	Figure
Observations	$N = 32$	$N = 18$	$N = 21$		
Ramification Index	1.50 ± 0.10	1.50 ± 0.12	1.45 ± 0.10	$p = 0.873$	S1-C
Soma Area (μm^2)	95.62 ± 6.74	98.21 ± 7.46	94.00 ± 11.26	$p = 0.596$	S1-C
Max Neurite (μm)	121.49 ± 6.07	109.70 ± 9.07	112.75 ± 9.39	$p = 0.274$	S1-C

	WT	WT+NT-ASO	WT+ASO	p-value	Figure
Observations	$N = 5$	$N = 7$	$N = 4$		
Fold Change ($\log_2$)	0.66 ± 0.09	0.77 ± 0.16	0.25 ± 0.11	$p = 0.064$, $p = 0.109$	S2-B

	WT	WT+ASO	p-value	Figure
Observations	$N = 25$	$N = 17$		
Max I_K (pA/pF)	38.22 ± 3.65	34.08 ± 3.61	$p = 0.474$	S2-C
Max I_{Na} (pA/pF)	-53.37 ± 7.17	-44.01 ± 4.52	$p = 0.256$	S2-C
Capacitance (pF)	41.40 ± 4.40	53.73 ± 4.49	$p = 0.067$	S2-C
Resistance (GΩ)	1.18 ± 0.15	1.17 ± 0.24	$p = 0.542$	S2-C

	WT	WT+ASO	p-value	Figure
Observations	$N = 9$	$N = 10$		
AHP (mV)	-20.07 ± 1.19	-24.93 ± 1.27	$p = 0.013$ *	S2-D
Threshold (mV)	-35.35 ± 1.52	-35.59 ± 1.11	$p = 0.896$	S2-D
Rheobase (pA)	22.26 ± 2.72	28.42 ± 5.08	$p = 0.315$	S2-D
Amplitude (mV)	55.49 ± 2.86	59.27 ± 3.77	$p = 0.444$	S2-D

	WT	WT+ASO	p-value	Figure
Observations	$N = 10$	$N = 12$		
Ramification Index	1.87 ± 0.26	1.66 ± 0.16	$p = 0.482$	N/A

+ASO	ACSF	+NIF	+XE991	p-value	Figure
Observations	$N = 9$	$N = 5$	$N = 9$		
AHP (mV)	-25.05 ± 1.43	-22.79 ± 0.63	-16.22 ± 0.61	$p = 0.364$, $p < 0.001$ ns/***	S2-F/S2-G
Threshold (mV)	-38.35 ± 1.60	-39.28 ± 1.03	-42.72 ± 0.65	$p = 0.438$, $p = 0.032$ ns/*	S2-F/S2-G
Full Width (ms)	15.40 ± 1.54	24.31 ± 1.42	12.46 ± 1.71	$p = 0.007$, $p = 0.221$ **/ns	S2-F
AHP Area (mV·s)	-0.22 ± 0.24	0.98 ± 0.56	0.43 ± 0.16	$p = 0.029$, $p = 0.039$ */*	S2-F

Table S1: Electrophysiology analysis and statistics of ASO-treated KCNT1-p.R474H ENs (corresponding to Figures 1, S1, and S2). N specifies the number of cells assayed. Data

presented as mean +/- SEM. Two-sample two-tailed Wilcoxon test (box plots) or two-sample two-tailed t-test performed, depending on sample size and normality. All p-values are rounded to three significant figures and adjustments for multiple comparisons were considered where appropriate.

	Vehicle	+NT-ASO	+ASO	p-value	Figure
Observations	$N = 11$	$N = 13$	$N = 12$		
Frequency at 180 pA (Hz)	5.00 ± 0.60	7.60 ± 2.93	18.22 ± 2.17	$p = 0.006$ **	2-C
AP Rundown ΔAmplitude (mV)	-9.22 ± 1.08	-7.21 ± 1.45	-4.25 ± 1.41	$p = 0.011$ *	2-D
Spike Frequency Adaptation	1.45 ± 0.07	1.35 ± 0.09	1.33 ± 0.10	$p = 0.235$	2-D
sAHP (6-8 Hz) (mV·s)	1.45 ± 0.28	1.18 ± 0.13	0.10 ± 0.23	$p = 0.002$ **	2-E
sAHP (10-14 Hz) (mV·s)	1.05 ± 0.24	0.52 ± 0.19	-0.01 ± 0.20	$p < 0.001$ ***	2-E
sAHP (16-18 Hz) (mV·s)	0.46 ± 0.26	0.08 ± 0.17	-0.51 ± 0.11	$p = 0.002$ **	2-E

Vehicle	ACSF	+PAX	+APN	p-value	Figure
Observations	$N = 11$	$N = 8$	$N = 11$		
Frequency at 180 pA (Hz)	5.00 ± 0.60	22.57 ± 4.54	3.82 ± 1.63	$p = 0.026$, $p = 0.682$ */ns	2-F

+NT-ASO	ACSF	+PAX	+APN	p-value	Figure
Observations	$N = 13$	$N = 6$	$N = 7$		
Frequency at 180 pA (Hz)	7.60 ± 2.15	25.20 ± 7.96	4.86 ± 2.26	$p = 0.022$, $p = 0.446$ */ns	2-F

+ASO	ACSF	+PAX	+APN	p-value	Figure
Observations	$N = 12$	$N = 10$	$N = 10$		
Frequency at 20 pA (Hz)	1.50 ± 0.93	9.60 ± 1.33	9.00 ± 1.58	$p < 0.001$, $p < 0.001$ ***/***	2-F
Frequency at 100 pA (Hz)	12.50 ± 0.86	24.80 ± 3.80	4.80 ± 1.58	$p = 0.013$, $p = 0.002$ */**	2-F
Frequency at 180 pA (Hz)	18.22 ± 2.17	25.00 ± 5.17	3.00 ± 0.45	$p = 0.271$, $p < 0.001$ ns/***	2-F

	WT	KOLF	R474H (Vehicle)	p-value	Figure
Observations	$N = 6$	$N = 8$	$N = 11$		
Frequency at 10 pA (Hz)	4.67 ± 0.92	5.75 ± 2.05	1.27 ± 0.70	$p = 0.014$, $p = 0.058$ */ns	S4-A
Frequency at 20 pA (Hz)	10.33 ± 2.05	13.75 ± 3.08	5.64 ± 0.96	$p = 0.041$, $p = 0.024$ */*	S4-A
Frequency at 30 pA (Hz)	12.33 ± 2.85	17.00 ± 2.72	8.73 ± 0.90	$p = 0.179$, $p = 0.011$ ns/*	S4-A
Frequency at 180 pA (Hz)	3.33 ± 0.67	7.00 ± 2.52	5.00 ± 0.60	$p = 0.185$, $p = 0.654$	S4-A

	WT	WT+ASO	p-value	Figure
Observations	$N = 6$	$N = 7$		
Frequency at 180 pA (Hz)	3.33 ± 0.67	6.00 ± 0.76	$p = 0.037$ *	S4-B
Maximum Frequency (Hz)	14.33 ± 2.70	28.29 ± 7.93	$p = 0.148$	N/A
Spike Frequency Adaptation	1.26 ± 0.12	2.03 ± 0.76	$p = 0.376$	N/A
sAHP (6-10 Hz) (mV·s)	0.85 ± 0.19	$0.09 \pm XX$	$p = 0.006$ **	S4-B

	Vehicle	+NT-ASO	+ASO	p-value	Figure
Observations	$N = 11$	$N = 13$	$N = 12$		
Maximum Frequency (Hz)	14.36 ± 0.80	17.23 ± 0.98	20.33 ± 1.32	$p = 0.003$ **	S4-C
Max Stim Current (pA)	59.09 ± 9.86	73.85 ± 14.12	126.67 ± 14.84	$p = 0.001$ **	S4-C

+ASO	ACSF	+NIF	+XE991	p-value	Figure
Observations	$N = 12$	$N = 5$	$N = 8$		
Frequency at 180 pA (Hz)	18.22 ± 2.17	N/A	38.25 ± 7.44	N/A, $p = 0.017$ *	S4-D

Table S2: Electrophysiology values and statistics for excitability and sAHP of ASO-treated KCNT1-p.R474H ENs (corresponding to Figures 2 and S4). N specifies the number of cells assayed. Data presented as mean +/- SEM. Two-sample two-tailed

Wilcoxon test (box plots) or two-sample two-tailed t-test performed, depending on sample size and normality. All p-values are rounded to three significant figures and adjustments for multiple comparisons were considered where appropriate.

Fetal Neurons	Early Midgestation	Late Midgestation	p-value	Figure
Observations	$N = 26$	$N = 27$		
Max I_K (pA/pF)	49.97 ± 4.12	53.03 ± 5.35	$p = 0.886$	3-E
Max $I_{K_{Na}}$ (pA/pF)	14.15 ± 6.48	23.00 ± 7.27	$p = 0.409$	3-E
$I_{K_{Na}}$ at 0 mV (pA/pF)	4.15 ± 1.06	11.35 ± 1.99	$p = 0.003$ **	3-E
$V_{1/2}$ of $I_{K_{Na}}$ (mV)	10.03 ± 1.70	0.39 ± 1.14	N/A	3-E

Fetal Neurons	Early Midgestation	Late Midgestation	p-value	Figure
Observations	$N = 28$	$N = 30$		
Ramification Index	2.79 ± 0.24	2.59 ± 0.17	$p = 0.634$	S1-E
Soma Area (μm^2)	79.47 ± 5.43	78.12 ± 4.10	$p = 1.000$	S1-E
Max Neurite (μm)	136.12 ± 6.12	152.37 ± 5.58	$p = 0.063$	S1-E

Fetal Neurons	Early Midgestation	Late Midgestation	p-value	Figure
Observations	$N = 26$	$N = 27$		
Capacitance (pF)	30.58 ± 2.28	33.78 ± 2.71	$p = 0.208$	S1-D
Resistance (GΩ)	1.73 ± 0.20	1.36 ± 0.19	$p = 0.244$	S1-D

23 WPC Sample	Vehicle	+NT-ASO	+ASO	p-value	Figure
Observations	$N = 21$	$N = 16$	$N = 20$		
Capacitance (pF)	31.97 ± 3.20	27.89 ± 3.89	32.63 ± 3.15	$p = 0.990$	4-C
Resistance (GΩ)	1.28 ± 0.24	1.60 ± 0.30	0.90 ± 0.17	$p = 0.409$	N/A
Max I_K (pA/pF)	58.02 ± 5.93	55.69 ± 5.61	43.28 ± 2.72	$p = 0.032$ *	4-C

15 WPC Sample	Vehicle	+NT-ASO	+ASO	p-value	Figure
Observations	$N = 30$	$N = 17$	$N = 22$		
Ramification Index	2.59 ± 0.17	2.42 ± 0.21	2.54 ± 0.24	$p = 0.669$	4-B
Soma Area (μm^2)	78.12 ± 4.10	76.66 ± 7.34	80.26 ± 6.05	$p = 0.920$	4-B
Max Neurite (μm)	152.37 ± 5.58	127.83 ± 10.11	123.79 ± 8.79	$p = 0.050$, $p = 0.027$ */*	4-B

15 WPC Sample	Vehicle	+NT-ASO	+ASO	p-value	Figure
Observations	$N = 8$	$N = 5$	$N = 9$		
Rheobase (pA)	51.35 ± 5.05	59.20 ± 5.58	51.68 ± 6.37	$p = 0.969$	S6-D
Threshold (mV)	-38.56 ± 0.78	-39.14 ± 0.54	-41.68 ± 1.20	$p = 0.052$	S6-D
Amplitude (mV)	67.28 ± 3.97	62.45 ± 2.12	72.95 ± 5.63	$p = 0.434$	N/A
AHP (mV)	-18.98 ± 1.48	-19.29 ± 0.61	-17.32 ± 1.05	$p = 0.365$	4-D
Half Width (ms)	2.50 ± 0.21	2.83 ± 0.24	2.86 ± 0.46	$p = 0.508$	N/A
Latency (ms)	28.98 ± 4.13	34.87 ± 2.21	35.55 ± 2.03	$p = 0.160$	N/A
ADP Tau	13.70 ± 0.86	15.92 ± 1.38	15.82 ± 1.55	$p = 0.266$	N/A

Table S3: Electrophysiology analysis and statistics of K_{Na} currents in primary fetal neurons, including ASO knockdown (corresponding to Figure 3, 4, and S6). N specifies

the number of cells assayed. Data presented as mean +/- SEM. Two-sample two-tailed Wilcoxon test (box plots) or two-sample two-tailed t-test performed, depending on sample size and normality. All p-values are rounded to three significant figures and adjustments for multiple comparisons were considered where appropriate.

iGABA Neurons	WT	Vehicle	p-value	Figure
Observations	$N = 8$	$N = 11$		
Max I_K (pA/pF)	40.40 ± 6.86	61.59 ± 6.88	$p = 0.031$ *	5-C
Capacitance (pF)	50.66 ± 3.86	48.46 ± 5.31	$p = 0.759$	5-C
Resistance (GΩ)	1.22 ± 0.21	1.13 ± 0.35	$p = 0.841$	N/A

iGABA Neurons	Vehicle	+NT-ASO	+ASO	p-value	Figure
Observations	$N = 11$	$N = 8$	$N = 15$		
Max I_K (pA/pF)	61.59 ± 6.88	58.53 ± 5.65	45.57 ± 6.17	$p = 0.024$	5-D
Capacitance (pF)	48.46 ± 5.31	44.33 ± 3.83	42.19 ± 4.32	$p = 0.365$	5-D
Resistance (GΩ)	1.13 ± 0.35	1.12 ± 0.25	1.47 ± 0.33	$p = 0.484$	N/A

iGABA Neurons	Vehicle	+NT-ASO	+ASO	p-value	Figure
Observations	$N = 12$	$N = 10$	$N = 23$		
Ramification Index	2.12 ± 0.22	2.07 ± 0.32	2.36 ± 0.32	$p = 0.620$	5-E
Max Neurite (μm)	118.98 ± 9.43	116.48 ± 9.50	116.57 ± 6.11	$p = 0.826$	5-E

iGABA Neurons	Vehicle	+NT-ASO	+ASO	p-value	Figure
Observations	$N = 11$	$N = 8$	$N = 12$		
AHP (mV)	-25.98 ± 1.24	-24.25 ± 0.97	-26.37 ± 1.61	$p = 0.852$	5-F
Threshold (mV)	-34.32 ± 1.00	-36.15 ± 0.98	-33.91 ± 0.98	$p = 0.773$	5-F
Rheobase (pA)	50.27 ± 7.78	48.45 ± 5.79	54.52 ± 8.95	$p = 0.726$	5-F
Amplitude (mV)	46.27 ± 2.11	46.16 ± 1.74	47.90 ± 2.51	$p = 0.627$	5-F
Downstroke Slope (mV/ms)	-16.80 ± 2.57	-21.53 ± 1.73	-24.18 ± 2.97	$p = 0.077$	5-F

iGABA Neurons	Vehicle	+NT-ASO	+ASO	p-value	Figure
Observations	$N = 11$	$N = 8$	$N = 12$		
Frequency at 100 pA (Hz)	13.09 ± 2.52	7.5 ± 1.05	22.67 ± 3.54	$p = 0.034$ *	5-G
Frequency at 180 pA (Hz)	8.73 ± 3.17	5.00 ± 0.76	21.5 ± 5.14	$p = 0.065$	5-G
AP Rundown ΔAmplitude (mV)	-9.01 ± 1.56	-7.61 ± 1.13	-2.79 ± 1.12	$p = 0.006$ **	5-G

Table S4: Electrophysiology values and statistics for ASO-treated KCNT1-p.R474H INs (corresponding to Figures 5 and S6). N specifies the number of cells assayed. Data presented as mean +/- SEM. Two-sample two-tailed Wilcoxon test (box plots) or two-sample two-tailed t-test performed, depending on sample size and normality. All p-values are rounded to three significant figures and adjustments for multiple comparisons were considered where appropriate.

GENE	baseMean	log2FoldChai	lfcSE	pvalue	padj	diffexpressed	delabel
CACNA1A	12341.1667	-0.0476122	0.05763003	0.05422611	0.70462335	NO	CACNA1A
CACNA1C	9511.10312	-0.0666291	0.04764177	0.01897107	0.50402158	NO	CACNA1C
CACNA1I	9335.45463	-0.0237209	0.03126509	0.18029622	0.96101416	NO	CACNA1I
CLCN2	1921.71204	0.01039898	0.03532747	0.404732	0.99680175	NO	CLCN2
CLCN4	45092.4558	-0.0468156	0.02879011	0.04511213	0.67494918	NO	CLCN4
GAPDH	325696.322	0.00345	0.01922567	0.83299562	0.99680175	NO	GAPDH
GPI	68889.184	0.01291161	0.02216633	0.42389168	0.99680175	NO	GPI
HCN1	5593.82774	-0.2080837	0.05702033	1.29E-05	0.02514354	NO	HCN1
HCN2	17920.9695	0.03690536	0.04487693	0.0892403	0.81674617	NO	HCN2
HCN3	11529.6982	0.00206242	0.02907957	0.90879793	0.99680175	NO	HCN3
HCN4	8263.89286	-0.00899	0.03345997	0.53231656	0.99680175	NO	HCN4
KCNA1	2440.66609	-0.0081317	0.03461674	0.52487596	0.99680175	NO	KCNA1
KCNA2	15617.0687	-0.1146764	0.06346595	0.00393506	0.29185245	NO	KCNA2
KCNA3	2034.44098	-0.0064947	0.03438876	0.57075077	0.99680175	NO	KCNA3
KCNA4	2039.53229	-0.0005806	0.03393326	0.96551452	0.99680175	NO	KCNA4
KCNB1	14181.5745	-0.0642899	0.06434818	0.03579471	0.62222352	NO	KCNB1
KCNB2	1484.99555	-0.0146563	0.0376799	0.16717826	0.94496995	NO	KCNB2
KCNC1	17527.4668	-0.0753306	0.04674114	0.01269794	0.42910553	NO	KCNC1
KCNC2	13463.657	-0.1135143	0.0560678	0.00265182	0.24940202	NO	KCNC2
KCNC3	5625.59232	-0.067695	0.06632113	0.0313985	0.59383338	NO	KCNC3
KCNC4	11679.8063	-0.0121291	0.02825824	0.48236046	0.99680175	NO	KCNC4
KCND1	4303.83955	0.03272794	0.04439725	0.10729247	0.86303378	NO	KCND1
KCND2	6559.81056	-0.0366642	0.045256	0.10217995	0.85035069	NO	KCND2
KCND3	20054.6615	-0.0065003	0.02739865	0.79909409	0.99680175	NO	KCND3
KCNJ2	1199.55008	-0.0032757	0.03561297	0.64472271	0.99680175	NO	KCNJ2
KCNJ3	3496.4602	-0.2374545	0.11175476	0.00124376	0.18237085	NO	KCNJ3
KCNJ5	657.668699	0.00333229	0.03589195	0.64424349	0.99680175	NO	KCNJ5
KCNMA1	12569.9313	-0.0014098	0.02928763	0.94860458	0.99680175	NO	KCNMA1
KCNMB1	89.6707655	-0.0007332	0.03633601	0.78676471	0.99680175	NO	KCNMB1
KCNMB2	861.338136	0.00794407	0.03634786	0.41709871	0.99680175	NO	KCNMB2
KCNMB3	48.7561741	-0.0014531	0.03640227	0.53087167	0.99680175	NO	KCNMB3
KCNMB4	5139.30857	0.02088939	0.03616599	0.2503039	0.99680175	NO	KCNMB4
KCNN1	3138.54184	-0.0012847	0.03455773	0.9073338	0.99680175	NO	KCNN1
KCNN2	359.219469	0.00166033	0.03612043	0.74364202	0.99680175	NO	KCNN2
KCNN3	1194.83362	0.00130577	0.0349454	0.9015089	0.99680175	NO	KCNN3
KCNN4	93.3589667	-0.0029122	0.03648459	0.31593807	0.99680175	NO	KCNN4
KCNQ2	73483.3057	0.00980761	0.02346562	0.37859899	0.99680175	NO	KCNQ2
KCNQ3	29513.2812	-0.1798784	0.14930131	0.00789865	0.36312044	NO	KCNQ3
KCNQ4	204.728275	0.00539856	0.03670636	0.26272253	0.99680175	NO	KCNQ4
KCNQ5	535.50981	-0.0056516	0.03598044	0.51156942	0.99680175	NO	KCNQ5
KCNT1	3445.91978	-0.8796788	0.06854893	4.08E-39	1.71E-34	DOWN	KCNT1
ORAI1	912.963191	-0.0051983	0.03577385	0.54930576	0.99680175	NO	ORAI1
RPLP0	76718.1841	0.0714708	0.0340112	0.00570047	0.33154847	NO	RPLP0
SCN1A	5487.20526	-0.0108264	0.037816	0.1830985	0.96422206	NO	SCN1A
SCN2A	45728.7642	-0.046698	0.03591924	0.04776546	0.68589395	NO	SCN2A
SCN3A	66012.9543	-0.0752587	0.03825394	0.0061955	0.33304769	NO	SCN3A
SCN8A	18964.6701	-0.1470233	0.06235501	0.00066333	0.13521941	NO	SCN8A
SCN9A	31194.0008	-0.050532	0.03837963	0.03799323	0.63510018	NO	SCN9A
STIM1	9802.25548	0.03205882	0.04156363	0.13472777	0.90771429	NO	STIM1

Table S5. RNAseq data, AP genes analyzed for ASO-treated ENs vs. control ENs

ALL SIG GENE	baseMean	log2FoldChai	lfcSE	pvalue	padj	diffexpressed
ADGRG4	1883.69736	-0.412176	0.12096859	2.52E-05	0.03423138	DOWN
ANKRD19P	592.561035	0.5559084	0.18047998	7.24E-05	0.04966497	UP
ARRDC4	2960.20516	0.28455355	0.08353697	2.79E-05	0.03423138	UP
CACNG8	11460.0306	-0.2875544	0.06690572	8.34E-07	0.00348714	DOWN
CHRM3	2206.05035	-0.2590534	0.07846691	4.12E-05	0.03742329	DOWN
EPS8L1	2730.27357	-0.360353	0.09840088	1.00E-05	0.02328637	DOWN
FLT4	131.28024	1.29140388	0.4050613	4.89E-05	0.04260604	UP
FOXD4L3	28.2799529	-2.2665068	0.72157415	5.67E-05	0.04486234	DOWN
GATM	1025.31216	0.38070178	0.12240718	6.87E-05	0.04946321	UP
HMGB1P6	2318.69422	0.28949235	0.09097759	5.89E-05	0.04550457	UP
KCNT1	3445.91978	-0.8796788	0.06854893	4.08E-39	1.71E-34	DOWN
KSR1	10265.2595	-0.2690856	0.04283686	1.70E-11	3.55E-07	DOWN
LOC124901924	279.037812	0.76126795	0.24818099	7.12E-05	0.04966497	UP
LOC727884	10.7610494	6.80885205	2.81848862	3.76E-05	0.0373303	UP
MACROD2	8815.66799	-0.2555988	0.05321802	7.43E-08	0.0006208	DOWN
MPHOSPH10	4427.70834	0.27266341	0.0563951	6.52E-08	0.0006208	UP
MVP	2587.18829	0.37767636	0.11448412	3.80E-05	0.0373303	UP
NDRG1	6326.61614	0.31176219	0.09312336	3.46E-05	0.0373303	UP
NPBWR2	24.7555079	2.91347213	0.94536014	6.67E-05	0.04900165	UP
NPC2	2587.90418	0.25987904	0.08326108	7.52E-05	0.04991136	UP
PCDH19	10796.9142	-0.281792	0.06670894	1.33E-06	0.00504509	DOWN
RPS27P29	8.10668476	6.75458666	2.80120117	3.84E-05	0.0373303	UP
RTP5	346.573489	-0.8008236	0.22301019	1.21E-05	0.02514354	DOWN
SERPING1	2445.88965	-0.2625466	0.07633817	2.38E-05	0.03423138	DOWN
SLC6A6	6479.00283	-0.2599406	0.0767132	3.03E-05	0.03493742	DOWN

Table S6. RNAseq data, All differentially expressed genes for ASO-treated ENs vs. control ENs

Table S7. KEY RESOURCES TABLE

REAGENT or RESOURCE	SOURCE	IDENTIFIER
**Antibodies and Probes**		
Rabbit anti-TUJ1 (beta-III tubulin)	ABCAM	AB18207
Mouse anti-MAP2	Invitrogen	MA512823
Rat anti- GFAP	Invitrogen	13-0300
Rabbit anti-GABA	Invitrogen	PA5-32241
VIM (*in situ*)	ACD	310441
GFAP (*in situ*)	ACD	311801
RBFOX3 (*in situ*)	ACD	415591
EOMES (*in situ*)	ACD	429691
KCNT1 (*in situ*)	ACD	545541
SST (*in situ*)	ACD	310591
BCL11B (*in situ*)	ACD	425561
HOPX (*in situ*)	ACD	423001
CUX2 (*in situ*)	ACD	425581
PVALB (*in situ*)	ACD	422181
TBR1 (*in situ*)	ACD	425571
VIP (*in situ*)	ACD	452751
4',6-Diamidino-2-Phenylindole, Dihydrochloride (DAPI)	Thermo Scientific	62248
Alexa Fluor 488, 594, 650 donkey anti-Mouse/Rabbit/Rat/Chicken	Invitrogen	A327XX, A12924, A78952
Chicken anti-MAP2	Invitrogen	PA1-10005
Rabbit anti-Cleaved Caspase-3	Cell Signaling Technology	9661S

**Biological Samples**		
Human Fetal Tissue (Primary)	This paper	
Human Postnatal Tissue	BioBank	UMBN 4353
Human Infant Tissue (Frozen/Fixed)	BioBank	HCT17HEIA029
**Chemicals, Peptides, and Recombinant Proteins**		
Hibernate E	Gibco	A1247601
Dulbecco's modified Eagle’s medium F-12	Gibco	21331-020
BrainPhys Media	Stem Cell Tech	05790
Fetal bovine serum	Gibco	35011CV
Poly-ornithine	Sigma	P4957-50ML
Laminin	Thermo	23017015
Neurobasal plus	Thermo	A3582901
Neurobasal	Gibco	21103049
Glutamax	Gibco	35050061
NEAA	Gibco	11140050
N2	Thermo	17502048
B27	Thermo	17504044
Y27632 (ROCK inhibitor)	StemCell Tech	72305
FGF	Thermo	PHG0264
EGF	Thermo	PHG0314
SB431542	Tocris	1614

XAV939	Tocris	3748
BDNF	Millipore	GF301
NT3	PeproTech	45003
Puromycin	Gibco	A1113803
Doxycycline	Fisher	BP26531
Apamin	Cole-Parmer	UX-79751-71
Paxilline	Tocris	2006
TRIzol Reagent	Invitrogen	15596026
Chloroform (Ethanol as Preservative/Certified ACS)	Fisher Chemical	BPC298500
**Commercial Assays**		
RNAscope Multiplex In situ kit	Advanced Cell Diagnostic	323110
**Deposited Data**		
Bulk Cortical Transcriptome	Allen Brain Atlas: BrainSpan project data	www.developing humanbrain.org
RNAseq: NGN2 Neurons treated with ASO	Nakayama et al, 2026	
**Experimental Models: Cell Lines**		
PGP1	Personal Genome Project	

KOLF	Pantazis CB, et al. 2022.	doi:10.1016/j.stem.2022.11.004
R474H	Nakayama T, et al 2026	
Primary Neurons	This paper	
**Plasmids**		
PTet-O-Ngn2-puro	Addgene	52047
PLenti CMV rtTA3 Blast	Addgene	26429
PB-TO-ASCL1-DLX2	Addgene	182307
**Kits and other reagents**		
RNeasy micro kit	QIAGEN	74004
M-PER mammalian protein extraction	Thermo	78501
SuperScript IV first strand synthesis system	Invitrogen	18091050
SYBR Green Master Mix	Applied Biosystems	A25742
DNA Clean & Concentrator-5	Zymo Research	D4004
Maxima H Minus cDNA Synthesis Master Mix	Thermo	M1662
QIAquick PCR purification kit	Qiagen	28104
Click-iT™ Plus TUNEL Assay Kits for In Situ Apoptosis Detection	Thermo	C10617
Zymo RNA Clean & Concentrate Kit 5	Zymo Research	R1013
Maxima H Minus First Strand cDNA Synthesis Kit	Thermo Fisher	M1662
QIAquick PCR Purification Kit	Qiagen	28104

Software and Algorithms		
Fiji/ImageJ	Rasband, W.S., ImageJ, U. S. National Institutes of Health, Maryland	http://imagej.nih.gov/ij/
Zen Black/Blue	Zeiss	
Igor Pro 7	Wave Metrics	
Adobe Illustrator CC 2017	Adobe	
**Other**		
SuperFrost Plus slides	Fisher Scientific	
ImmEdge Pen	Vector Labs	H-4000
Zeiss AXIO observer	Zeiss	
Zeiss LSM700 confocal microscope	Zeiss	
Leica Research Cryostat	Leica	
Sutterpatch IPA	Sutter Instruments	
QuantStudio 6 Pro	Applied Biosystems	
Synergy LX	Agilent	
