# Supplementary material for: RNA targeting therapy for a prenatally enriched potassium channel associated with severe childhood epilepsy and premature death
Source: Nat Commun. 2026 Apr 29;17:5864. doi: 10.1038/s41467-026-72334-7 (PMC13333874; doi:10.1038/s41467-026-72334-7)
Supplement: Supplementary file 2 — Transparent Peer Review file [file 41467_2026_72334_MOESM2_ESM.pdf]

nature portfolio

Peer Review File

RNA targeting therapy for a prenatally enriched potassium channel associated with severe childhood epilepsy and premature death

Corresponding Author: Dr Richard Smith

This file contains all reviewer reports in order by version, followed by all author rebuttals in order by version.

Version 0:

Reviewer comments:

Reviewer #1

(Remarks to the Author)
Sean and colleagues reported the electrophysiological onset of KNa1.1 in human brain development and the cellular basis for the first-in-human application of a therapeutic RNA targeting KCNT1-associated epilepsy in newborns. In human-induced neurons heterozygous for a pathogenic KCNT1 variant (p.R474H), antisense oligonucleotide (ASO) treatment reduced gain-of-functon(GOF) KNa1.1 currents, increased after-hyperpolarization amplitude, and improved the dynamic range of neuronal burst firing. They also demonstrated the feasibility of ASO targeting the KNa1.1 channel in mid-gestational primary neocortical neurons, thus establishing a developmental basis for KCNT1-associated pathophysiology and drug interventions prior to the postnatal onset of epileptic encephalopathy. Here, I have a few concerns that should be addressed before publication.

Majors :

1. Figure 1B and Figure 4C: The authors need to prove whether ASO directly inhibited the sodium-dependent potassium current. The authors found that ASO inhibited steady-state outward K+ currents in KCNT1-p.R474H neurons and human fetal primary neurons, in Figure 1B and Figure 4C, respectively. However, the steady-state K+ current contained multiple components and could not prove the direct effect of ASO on KCNT1-induced currents or sodium-dependent potassium currents.

2. Figure 1C and Figure 2A: Based on previous research, different potassium channels mediate afterhyperpolarization (AHP) at distinct time scales and are categorized as fast afterhyperpolarization (fAHP, activated in 1–5 ms), medium afterhyperpolarization (mAHP, activated between 10–300 ms), and slow afterhyperpolarization (sAHP, activated between 0.5-multiple seconds) . The mAHP amplitude defined in Figure 1C is a mixture of fAHP and mAHP, while the sAHP defined in Figure 2A is a mixture of mAHP and sAHP. BK channels mainly contributes to fAHP, and SK channels mainly contributes to mAHP and sAHP, which can explain that the antagonists of both BK channels and SK channels can affect the mAHP amplitude depicted in Figure 1E. Moreover, the definination and quantification of sAHP need to be explained in the method.

3. Figure 1C-D: the authors found that ASO affects the characteristics of mAHP. What are the components of mAHP in KCNT1-p.R474H neurons? In hippocampal CA1 neurons, HCN, SK, Kv7 channels contribute to mAHP, while BK mainly contributes to fAHP. Why did the authors only use BK and SK antagonists to treat KCNT1-p.R474H neurons? Meanwhile, KCNT1channel directly contributes to both mAHP and sAHP, and ASO inhibition of KCNT1 could directly change the characteristics of mAHP and sAHP. The conclusion that ASO affects mAHP through BK and SK channels seems unreasonable.

4. Figure 1C-G: ASO treatment appears to increase mAHP through calcium-activated potassium channels (BK and SK) in Figure 1C-G. The effects of ASO on BK and SK channels raise concerns about the specificity of ASO for KCNT1 targeting. The authors should address the mechanism of action (MOA) of the ASO treatment in greater detail, particularly its potential off-target effects.

5. Figure 2B: it is necessary to supplement the action potential burst of WT neurons to demonstrate that ASO can restore the

abnormalities in neuronal excitability caused by mutations.

6. The differences in ASO' effects on mAHP between primary neurons and NGN2-derived neurons requires further explanation.

7. In vivo models would provide more evidence to confirm the efficacy of ASO for KCNT1-related epilepsy.

Minors：
1. Page 8: The article “Antisense oligonucleotide therapy for KCNT1 epilepsy of infancy with migrating focal seizures” has been cited several times in this manuscript, but it is not yet online at present.
2. Figure 1B should include a schematic diagram of voltage-clamp stimulation, and Fig 1C needs to label the holding potential and current injection potocol.
3. Figure 1G is not cited in the manuscript.
4. Figure S2A: The last panel is deficient in the ordinate.

Reviewer #2

(Remarks to the Author)
There are some interesting and important results presented in this manuscript. These fall into two separate categories. The results that are presented in the second half of the manuscript (but the first part of the abstract) are potentially the most straightforward and important. Using both RNA profiling and patch clamp recordings, the authors show that KNa1.1 (KCNT1) channels are expressed in human brain in both excitatory and inhibitory neurons at mid gestation They demonstrate that suppression of these channels using an ASO reduces the ability of the immature neurons to fire at high rates, suggesting that, under normal conditions, the biological role of these channels is to promote repetitive firing. These experiments provide a firm basis for making decision about the optimal timing to begin therapeutic treatments for conditions caused by gain-of-function mutations in KCNT1. Because there is currently much work in this therapeutic area, the findings will be particularly relevant for these efforts.
The other part of the work, which is presented first in the body of the results section, is also very interesting, clearly presented and carried out rigorously. I believe, however, that it is a little more difficult to interpret, and could benefit from a clearer statement about the different possibilities. It describes the effects of the ASO that suppresses KNCT1 expression on ionic currents and firing patterns of neurons differentiated from iPSC cells of a patient with the KCNT1-pR474H mutation. The neurons were differentiated using lentiviral-mediated overexpression of NGN2. The effect of the ASO was quite different from what was found with the human neurons in primary culture. The ASO was found to have a complex effect on excitability, suppressing firing in response to small depolarizing currents but increasing firing in response to larger stimuli. This was accompanied by an increase in both medium and slow afterhyperpolarizations (mAHP and sAHP) that follow a single action potential or a train of action potentials. Pharmacological experiments indicated that these effects of the ASO were due to increases in BK and SK calcium-activated potassium currents.
The rationale for testing an ASO is that suppression of KCNT1 expression may reverse the effect of a gain-of-function mutation. It is possible, for example, that reducing the levels of KCNT1 proteins promotes the expression of other channels by a mechanism distinct from lowering KNa1.1 activity. One difficulty, therefore, in interpreting the effects of the ASO on the KCNT1-pR474H cells is that it not known if the mutation itself produces a loss of BK and SK currents. If these were the case, this would provide a powerful case for the utility of these ASOs. While the manuscript provides some data (Fig. S1G) that the ASO also increases the mAHP in a control NGN2 cell line, there is insufficient information to determine whether the properties of control cells are distinct from those of the KCNT1-pR474H cells. From the present data, therefore, it is not possible to determine if the ASO: 1) reverses the effects of the gain-of-function mutation, 2) produces changes in ion channel expression that are distinct from the effects of the increased KNa current or 3) has effects that are specific to NGN2-differentiated cells but not seen in native neurons. Some of this could be addressed by more explicit statements about these potential effects of the ASO, or by providing more information on control cells.

Specific comments:

1) The work found that treatment of human primary neurons with the ASO reduces their excitability, suggesting that the normal role of KNa channels in these neurons is to increase their ability to fire at high rates. An increased ability to fire at high rates with increased KNa current has been found for another KCNT1 gain-of-function mutation in human iPSC-derived neurons (Quraishi et al., J. Neurosci., 39:7438, 2019) and also in cortical pyramidal neurons of mice expressing the same KCNT1 mutation studied here (Wu et al. Cell Reports 43:113904, 2024). This suggests that the work with the human primary neurons likely reflects effects on excitatory neurons, and it would be appropriate to cite both these previous publications in the manuscript.

2) The study of cortical neurons in mice with the corresponding KCNT1-pR474H mutation (Wu et al.,) found that, in contrast to pyramidal cells, action potential firing in inhibitory neurons was suppressed by the increased KNa1.1 current. This was also found in a mouse study of a different Kcnt1 mutation that is referenced in the third paragraph on page 20 (Shore et al.). While the manuscript describes the NGN2-differentiated KCNT1-pR474H neurons as “cortical excitatory neuron”, the more complex response of these cells to the ASO suggest that their phenotype may more closely resemble a hybrid of responses found in native excitatory and inhibitory neurons. Readers would be helped by an explicit clarification of the expected responses of gain-of-function and ASO suppression in different native neuronal types.

3) Text in the middle of the abstract of this manuscript makes the statement that the ASO reduced seizure occurrence and

severity. These results are not presented in this manuscript, but instead are described in a different manuscript that is related to the present work and is provided as supporting information. This information should not be placed in the "results" section of the abstract itself. In addition, the statement about the effects of the ASO on one of the patients (2nd paragraph, page 19) is unclear to the reader in the absence of this unpublished information.

Minor comments:

- A clarification is required for how the measurements of KCNT1 mRNA were quantified for Supplemental figure 1B. The legend state that gene expression levels in vehicle-treated cells were adjusted to one, but the figure itself shows a fold change of less than one for vehicle treatment in control.

- The text at the top of page 10 refers to figure S1D for the effect of the ASO on the mAHP in an cell line from a healthy individual. Presumably this is meant to be figure S1G.

-It is stated that on page 10 that nifedipine did not affect mAHP amplitude in ASO-treated neurons. The related figure (S1H), however, shows that the AHP is severely disrupted by nifedipine. The fact that the membrane potential returns to baseline within 200 msec of the end of the spike does not fully justify the statement that the mAHP is unaffected.

Reviewer #3

(Remarks to the Author)
Recommendation: Major revisions

This manuscript by Golinski et al evaluates the potential of antisense oligonucleotides (ASOs) for prenatal enriched potassium channel associated epilepsy. While this paper offers interesting insights into potential prenatal and early infantile ASO-mediated therapeutic options for EIMFS, there are both major and minor concerns that should be considered as listed below:

Major concerns
1. Many NGN2 neuron Ephys + morphology results in Figure 1 seem to be similar to experiments performed in Nature Medicine paper that was given to reviewers as related materials (Fig 2). Were these experiments performed by a different lab than what is reported in the Nature Medicine manuscript? Were they at least independent differentiations? Much of this Nature Communications manuscript seems like it is supplemental work that did not make it into the Nature Medicine manuscript and instead was put together in a new paper. There is nothing wrong with that, BUT, it should be very clear to the reviewers and the readers if this is "extra" data from the same set of experiments detailed in the Nature Medicine work, or if these are independent experiments. If they are not independent experiments, parts of this manuscript could be misconstrued as replication of the Nature Medicine findings, when in fact, it is not.
2. The authors refer to p.R474H neurons as mature at 14 days in culture, whereas most of the field would likely argue maturity is not reached until Days 21-28 at the very earliest (PMID: 29791859; PMID: 39255791). Typically, this is defined by the presence of active synapses and ephys network activities. The authors do not show neuronal activity until Day 28, so I suggest they remove the verbiage saying neurons are mature at 14 days OR show immunostaining and/or ephys to demonstrate functional maturation.
3. While the authors have assessed ASO induced changes to neuronal morphology, I am wondering if the authors have tested for other toxic factors such as cell viability? If so, please provide that data along with the neuronal morphology. This is important given that the patients described in the Nature Medicine had side effects that could potentially be a result of neuron death (e.g. enlarged ventricles).
4. In Figure 1B, the authors compare WT, Veh, Patient+NT-ASO, and Patient+ASO neurons for all assays. They present p-values that denote differences between Patient+NT-ASO, and Patient+ASO. It is unclear if there are any statistical differences between the WT control and the Patient+NT-ASO lines. If there are no differences, one could ask what exact phenotype are the authors trying to rescue? Along these same lines, the authors do not include the WT control in any of the subsequent assays. Again, leading one to ask, is the ASO rescuing these phenotypes to WT levels if there are indeed real differences that need to be corrected.
5. Even though no observable changes are seen in MAP2, GFAP, and Tuj1 in Figure 4A, but there is no quantification to support this claim. Even pixel signal intensity is sufficient to make this assertion, but representative images is not adequate.
6. It is fundamentally unclear what the authors' take home message is regarding the utility of the NGN2 models described early in the paper when the later experiments in primary neurons give the impression that the NGN2 model is not valid. The authors should make it clear if the NGN2 data is valuable to their conclusions, otherwise this data should be be removed. Alternatively, if the authors feel the need to make claims about NGN2 vs primary models, they should either do side-by-side comparisons or at the very least present these two models in the same section of the paper.
7. The quinidine experiments seemed uninformative, incomplete, and seemingly out of nowhere. Please either explain why these experiments are relevant by giving more context behind the motivation for these experiments in the grand scheme of the project's goals or cut them out entirely.
8. What is the purpose of Figure 5? It shows the KCNY1 prenatal localization and postnatal and ASO. This figure is not discussed at all, and there is no legend. Was this included by mistake?

Minor:
1. At some places, the figure call outs in the text do not match the actual figure panel. For example, the authors write - "However, the KCNT1 signal was negative in glial fibrillary acidic protein (GFAP) cells throughout the deep to superficial

cortex (Figure 3B)". But, figure 3B does not contain any GFAP panel. Please double check figure panels and text.
2. Figure panels should be mentioned in the text in the same order they are presented in the figure. For example, in Figure 1, Rheobase, mAHP, and threshold are discussed in this order in the text but not in the figure itself. This is also true for Figure 3C which is discussed after Figure 3E.
3. Again, there needs to be more consistency between figures and text. Eg, Fig. 1B shows WT-Neurons, but the results text only refers to this PGP-1 cell line. This can create confusion for the reader. The authors refer to apamin as APN in the text, but in Figure 1E it is referred to as Apa. Please double check figure panels and text for consistency.
4. Some figures have no reference at all in the text (e.g. Figure 1G). Please provide context for all figures included in the panels.
5. Although the statistical section in the methods says that "Figure legends include details of the statistical methods used for analysis", the figure legends just state the p-value but not the actual test carried out for specific figures. Please include information of the statistical testing carried out for each figure in the figure legends.
6. Figure 3A shows KCNT1 expression up to 15 yrs of age, which they refer to as adulthood. It seems the more correct term should be juvenile/teenage/adolescent.
7. Many parts of this paper are written in a way that is only accessible by experts in ephys. Nature Comms is a broad audience journal, so this needs to be addressed, especially when it comes to explanations of experimental design and interpretation. For example, "Moreover, the KCNQ2 channel antagonist XE991 did not reduce AP excitability in ASO-treated neurons, but instead increased high-frequency firing." The authors do not mention what KCNQ2 is and why exactly the use of its antagonist would yield this conclusion. In addition, see are above comment about the quinidine experiments and how they were not sufficiently described.
8. Grammar mistake: "Patient-derived iPSCs use NGN2 induction to generate a cortical-like excitatory neuron…". The iPSCs do not use NGN2 induction, the NGN2 induction is performed on the iPSCs.

Reviewer #4

(Remarks to the Author)
Golinski et al present a study entitled: Gene therapy for targeting a prenatally enriched potassium channel associated with severe childhood epilepsy and premature death. The authors report the electrophysiological onset of KNa1.1 in human brain development and the cellular basis for a first-in-human application of an ASO targeting KCNT1-associated EIMFS. The manuscript is clearly written and the data presentation is appropriate. The reviewer has a few comments and concerns.

Gene therapy is mentioned in the title. The reviewer wouldn't consider an ASO as gene therapy.

P3. According to the most recent classification by the ILAE, epileptic encephalopathy should be renamed as developmental and epileptic encephalopathy.

The abstract may be a bit misleading since the authors are mentioning the results of a clinical trial that is not a part of this study. If the results are relevant to mention in the abstract, then specify that it was a previously conducted study.

In the introduction the authors are referring to a Nature Medicine paper describing the results of a clinical trial with two children with KCNT1-related EIMFS, who were successfully treated with an ASO. The reviewer can't find that reference, has the paper been published? The results of a small ASO trial with two children with KCNT1 has previously been mentioned in New York Times. In that study both children developed severe hydrocephalus, and one child died because of this adverse event. Are the authors referring to the same study?, if yes then maybe this should be mentioned.

The authors are mentioning that half of KCNT1 EIMFS kids do not survive past the age of 3 years. The reviewer agree that there is an increased mortality in this patient population, but according to a large study of more than 150 KCNT1 EIMFS cases the mortality rate is not as high as 50% (PMID: 34114611).

It is unclear if CNV analysis has been performed in the p.(R474H) hIPSCs, and whether isogenic controls have been generated.

The obtained results suggests that ASO targeting of perinatal KNa1.1 GoF current might help limit disease progression compared with postnatal treatment. Do the authors have any suggestions on how these children can be diagnosed prenatally?

Reviewer #5

(Remarks to the Author)
KCNT1-linked early infantile developmental and epileptic encephalopathy (EIEE14, EIFMS) is a devastating disease caused by gain-of-functon(GoF) de novo variants in KCNT1, encoding KNa1.1, resulting in increased outward K+ currents and reduced excitability of cortical GABAergic interneurons, disrupted E:I balance, and overall network hyperexcitability. Here, iPSC NGN2-differentiated cortical-like excitatory neurons generated from a patient with a GoF KCNT1 variant, p.R474H, showed blunted excitability including abnormal action potential (AP) AHP kinetics, such that neurons cannot

maintain AP firing at high current injections. The authors investigate the mechanism of action of a recently developed, first-in-human, KCNT1-targeted RNAse-H activating ASO that previously showed significant reductions in seizure frequency and severity in two EIEE14 patients. They show ASO efficacy and specificity in lowering outward K+ currents in both patient-derived iPSC NGN2 neurons and primary human fetal neurons from mid-gestation neocortex. Because KCNT1 mRNA is shown to be expressed during mid-gestation brain development in humans, the authors make a case for early therapeutic intervention with prenatal ASO delivery, prior to full disease manifestation.

1. For completeness, the authors should include the OMIM term for KCNT1 linked early infantile developmental and epileptic encephalopathy, DEE14.

2. The authors do not indicate how many iPSC lines were generated from the patient samples. Due to the well-known high variability of iPSC neuron data, at least two lines from each patient/control sample should be generated and tested.

3. NGN2 differentiation skips over critical neuronal developmental time points. Because a goal of this work is to model an early infantile, possibly fetal, brain disorder, NGN2 differentiation may not be the most appropriate method. In contrast, dual SMAD differentiation, which more accurately models early brain development, would be more appropriate. This differentiation issue may explain why ASO treatment of fetal neurons did not recapitulate the NGN2 neuron response of increased mAHP – although being able to show this result in more than one iPSC cell line, as discussed above, may also be a factor.

4. What is the rationale for the use of iPSC pyramidal neurons for this study? How do the authors propose that expression of a GoF KCNT1 variant in cortical pyramidal neurons, resulting in reduced intrinsic excitability, contributes to epileptogenesis? Previous work in mice showed predominant KCNT1 expression in PV+ and SOM+ interneurons, with near-absence in other interneuron subsets (e.g. VIP+), with intermediate expression in excitatory pyramidal neurons. Others have shown that GoF KCNT1 variants linked to DEE14 significantly impact inhibitory interneuron excitability and disrupt E:I balance. Thus, this study would have greater impact if the authors included a study of inhibitory iPSC neurons in the presence and absence of ASO. While PV+ interneurons are difficult to generate in 2-D culture, many groups have successfully generated SOM+ interneurons.

5. What are the effects of ASO treatment on synaptic activity between patient neurons, especially on restoring inhibitory tone? Is the synaptic activity of control neurons altered by ASO treatment?

6. Does ASO treatment of iPSC or human neocortical neurons in culture impact/delay the maturation of GABAergic signaling from excitatory to inhibitory?

7. What is the specificity of the ASO for reducing KCNT1 mRNA vs. other K+ channel mRNAs and Na+ channel mRNAs?

8. Page 10 – Figure 1G does not appear to be discussed. This section lacks a concluding statement.

9. Does the expression pattern of KNa1.1 protein follow the KCNT1 mRNA expression pattern shown for human prenatal cortical neurons? With recent new knowledge regarding the alternative splicing of ion channel mRNAs, including the inclusion of nonsense-mediated decay/poison exons, this is an important question.

10. Fig. 3C appears out of order in the text of the results section.

11. What is the effect of quinidine on patient iPSC neurons?

Minor: The red, pink, magenta color scheme is sometimes difficult to discern, and the light pink traces want to fade into the background. I would use a more dynamic color scheme.

Reviewer #6

(Remarks to the Author)
I co-reviewed this manuscript with one of the reviewers who provided the listed reports. This is part of the Nature Communications initiative to facilitate training in peer review and to provide appropriate recognition for Early Career Researchers who co-review manuscripts.

Version 1:

Reviewer comments:

Reviewer #1

(Remarks to the Author)
I have carefully reviewed the authors’ detailed point-by-point responses and the accompanying revised manuscript. I am satisfied that the authors have thoroughly addressed all of my comments and concerns, including through the addition of new experiments, clarifications of methodology, and improved data presentation.

In my view, the revised manuscript now meets the standards for publication in Nature Communications. I therefore have no further concerns and recommend the manuscript for publication.

Reviewer #2

(Remarks to the Author)
The authors have added a substantial amount of new data to this manuscript. As presented, however, the overall message remains confusing. Specifically, the effects of a KCNT1 ASO have now been tested on three different cell types, and these effects of the ASO are different for each cell type. Superficially, this could lead one to conclude that such effects on human neurons are variable and unpredictable and would do a disservice to the development of these reagents for new therapeutic approaches. Nevertheless, the observed findings do make some sense in the context of what is currently known about the role of KCNT1 channels in different types of cortical neurons. The manuscript could therefore be improved by some careful rewriting, and by coupling its conclusions to what is already known of the role of KCNT1 channels in excitatory and inhibitory neurons.

Specific comments:

1) Probably the most important contribution of this study is the work with primary human neurons and the characterization of the prenatal emergence of KNa1.1 currents. Application of the ASO to these neurons results in a marked suppression of current-induced firing. These data are shown in figure 4E but (surprisingly!) are not mentioned at all in the results section.

2) The major stated conclusion of the manuscript is that ASO treatment increases action potential firing in neurons. This is stated in the "in brief" section, the abstract and in the first paragraph of the discussion ("increased action potential burst firing", "increased high-frequency spiking and burst properties" and "increase the maximal response for input-output excitability curves", respectively.) This is misleading because the effect of the ASO on human primary neurons was exactly the opposite; As stated above, in these neurons their major effect was to strongly suppress excitability.

3) Previous work from several groups with mouse models has shown that gain-of-function KCNT1 mutations suppress excitability of cortical inhibitory interneurons. The effect on excitatory pyramidal neurons is different in that, depending on the mutation, the gain-of-function either increases excitability or has neutral effects. The finding in the present work that the ASO increases the excitability of the IN-type cells and suppresses that of the primary cortical neurons, which are most likely excitatory cells, is fully consistent with this. The finding that the ASO also increases maximal firing rate of the EN-type cells appears, at first sight, to be inconsistent with this. But, as shown in this study, the ASO does suppress firing of the EN-type cells in response to lower current stimuli, consistent with both the primary neurons in this study and all the mouse work. The anomalous effect of the ASO on firing with stronger stimulation of the EN-cells may perhaps be explained by the uncertain nature of these cells (see comment 4).

4) The first three pages of the results section focus heavily on the effect of the ASO to induce an increase in a calcium-dependent afterhyperpolarization in NGN2-differentiated, putative excitatory neurons. This was not observed in either of the other two cell types. While pharmacological studies provided some clues, the nature and mechanism of this effect were not established. It remains a possibility, therefore, that this is a peculiarity of this cell differentiation protocol, and may not be relevant to the actions of ASO in normal neurons. While these findings may be worth reporting, it does not make sense to make this a major focus of the manuscript. It could potentially be put in supplemental data with appropriate caveats about significance.

5) In the first section of the results (p. 8-9) it is reported that the KCNT1 ASO reduced K+ current in KCNT1-p,R474H ENs but did not reduce K+ current in control PGP-1 ENs. This is in direct contrast to the effects of the ASO on primary human neurons, which, as stated above, are most likely excitatory neurons and in which the ASO suppressed K+ current despite the lack of channel mutation. A comparison of figures S1B and S2B shows, however, that there was much less knockdown of KCNT1 in the control PGP-1 ENs (S2B) compared to the mutant cells (S1B), such that levels of KCNT1 mRNA in one of the control samples appear identical to that in the untreated cells. This seems a likely explanation for the lack of effect in the control cells, as work from numerous groups indicates that KNa currents are present in control excitatory neurons (although it is also likely that the ENs are not representative of native neurons). This should be addressed, either experimentally or in discussion.

6) Previous work has shown that Na channel expression can be regulated by KCNT1. The results section (p.8) states that levels of inward sodium current density in KCNT1-p,R474H ENs were not altered by the ASO. Nevertheless, figure S1D shows that the amplitude of sodium current varies over an order of magnitude in different cells, making it difficult to make a firm statement on this. It would be helpful to know if there is similar variability in the K+ current (The error bars in the IV plot of figure 1B suggest there is not, but individual values are not shown in bar graph form). It might also be helpful to provide Na^+ current quantification for the human fetal neurons in response to the ASO.

Minor comments:

7) How do the authors validate that their electrophysiological recordings were performed on specific neuronal subtypes? For example, although Figure 5B shows robust GABA staining via immunohistochemistry, it is not clear how they ensured that the recorded cells were indeed GABAergic neurons, as there appear to be numerous cells that are negative for anti-GABA .

8) Page 8, line 3. Figure S2C should be S2D.

9) Page 19, second line- Figure 5B should be 5C.

10) Page 19. Improved rundown presumably means less rundown rather than more rundown? This should be clarified and/or a figure added to show what is meant.

11) The section about poison exons (p.15) is confusing because it is placed in the section on cell-type expression during development. It either needs a better opening rationale or to be transferred to a different section or to the discussion.

12) The sentence beginning " Analysis of the neocortex …" in the same section (p.15) has no verb.

13) The sentence in the discussion beginning "Follow-up studies of non-conducting…" (p.22) has no verb.

14) Last line of first paragraph in introduction (p.6); onset of encephalopathy.

Reviewer #3

(Remarks to the Author)
The authors have addressed all concerns.

Reviewer #4

(Remarks to the Author)
The authors have successfully addressed all the issues raised by the reviewer.

Reviewer #5

(Remarks to the Author)
I am satisfied with the Revised manuscript and have no further comments.

Reviewer #6

(Remarks to the Author)
I co-reviewed this manuscript with one of the reviewers who provided the listed reports. This is part of the Nature Communications initiative to facilitate training in peer review and to provide appropriate recognition for Early Career Researchers who co-review manuscripts.

Open Access This Peer Review File is licensed under a Creative Commons Attribution 4.0 International License, which permits use, sharing, adaptation, distribution and reproduction in any medium or format, as long as you give appropriate

credit to the original author(s) and the source, provide a link to the Creative Commons license, and indicate if changes were made.
In cases where reviewers are anonymous, credit should be given to 'Anonymous Referee' and the source.
The images or other third party material in this Peer Review File are included in the article’s Creative Commons license, unless indicated otherwise in a credit line to the material. If material is not included in the article’s Creative Commons license and your intended use is not permitted by statutory regulation or exceeds the permitted use, you will need to obtain permission directly from the copyright holder.
To view a copy of this license, visit https://creativecommons.org/licenses/by/4.0/

Golinski et al, Manuscript NCOMMS-24-63041A

REVIEWER COMMENTS

Reviewer #1

Sean and colleagues reported the electrophysiological onset of KNa1.1 in human brain development and the cellular basis for the first-in-human application of a therapeutic RNA targeting KCNT1-associated epilepsy in newborns. In human-induced neurons heterozygous for a pathogenic KCNT1 variant (p.R474H), antisense oligonucleotide (ASO) treatment reduced gain-of-functon(GOF) KNa1.1 currents, increased after-hyperpolarization amplitude, and improved the dynamic range of neuronal burst firing. They also demonstrated the feasibility of ASO targeting the KNa1.1 channel in mid-gestational primary neocortical neurons, thus establishing a developmental basis for KCNT1-associated pathophysiology and drug interventions prior to the postnatal onset of epileptic encephalopathy. Here, I have a few concerns that should be addressed before publication.

- We thank Reviewer 1 for their comments. We performed new experiments to address concerns and added text for clarity throughout the manuscript. Please see below a line-by-line review of the additions to help with clarifying changes.

Majors：

1. Figure 1B and Figure 4C: The authors need to prove whether ASO directly inhibited the sodium-dependent potassium current. The authors found that ASO inhibited steady-state outward K+ currents in KCNT1-p.R474H neurons and human fetal primary neurons, in Figure 1B and Figure 4C, respectively. However, the steady-state K+ current contained multiple components and could not prove the direct effect of ASO on KCNT1-induced currents or sodium-dependent potassium currents.

- Thank you for the opportunity to clarify this important point on the selectivity of our ASO. We hope our new data clarify that the lead ASO selectively reduces only the KCNT1 portion of the outward K^+ current, leaving other K^+ currents unaffected. We also now add a reference to the clinical manuscript “Nakayama et al., in revision” with *Nature Medicine* for *in silico* modeling and selection of a highly selective ASO from over 100 compounds to one lead compound.

- In Figure 1B, we first show that the outward K^+ current is increased in KCNT1-p.R474H neurons, and that treatment with the selective ASO reduces the large gain-of-function (GoF) outward current to levels comparable to wild-type (WT) outward K^+ channels (Figure 1B).

-To further validate the specificity of ASO-mediated knockdown in excitatory neurons (ENs) with normal levels of K_{Na}, we performed new voltage-clamp analyses using ASO-treated WT <u>control neurons (PGP1 line).</u> In these additional experiments, we did not observe a significant reduction in the outward K^+ component (new Figure S2C, $P = 0.48$), demonstrating that the ASO significantly reduces the outward current only in the presence of the large GoF KCNT1 current found in p.R474H patient neurons.

- To further support the selectivity of ASO-mediated knockdown, we now provide new RNA-sequencing (RNA-seq) data assessing the expression of all K^+ channel genes, as well as other ion channels underlying action potential kinetics, following ASO treatment of ENs. These data indicate no changes in the expression of any other K^+ channels or action potential-related genes, while KCNT1 is selectively knocked down (new Figures 1F & S1H). Also non-targeting ASO (NT-

ASO) does not alter KCNT1 expression in pR474H or control ENs (Figure S1B and S2B). We also provide new transcriptomic data tables of ASO-treated ENs, show a comparative cellular transcriptomic state in our cells (Tables S5, S6).
- Lastly, we provide data from a different cell type differentiation approach using a modified iGABA inhibitory neuron induction protocol[1]. New voltage-clamp recordings from p.R474H inhibitory iGABA neurons show that a GoF outward K^+ current (Figures 5C and D) is also reduced with the ASO, suggesting that the ASO-mediated knockdown is not a differentiation protocol-specific effect (i.e., NGN2 vs. iGABA).

2. Figure 1C and Figure 2A: Based on previous research, different potassium channels mediate afterhyperpolarization (AHP) at distinct time scales and are categorized as fast afterhyperpolarization (fAHP, activated in 1–5 ms), medium afterhyperpolarization (mAHP, activated between 10–300 ms), and slow afterhyperpolarization (sAHP, activated between 0.5-multiple seconds). The mAHP amplitude defined in Figure 1C is a mixture of fAHP and mAHP, while the sAHP defined in Figure 2A is a mixture of mAHP and sAHP. BK channels mainly contributes to fAHP, and SK channels mainly contributes to mAHP and sAHP, which can explain that the antagonists of both BK channels and SK channels can affect the mAHP amplitude depicted in Figure 1E. Moreover, the definination and quantification of sAHP need to be explained in the method.

- Thank you for this suggestion. To avoid confusion between fAHP and mAHP classification and temporally overlapping ionic conductances, we rename this more generally as "AHP" in the primary figures and clearly identify the analysis window in Figure 1C (10–200 ms following the action potential peak, measured in reference to threshold). Our analysis windows are now clearly stated in the Methods, based on the Andrade and Foehring review. [2]
- We also now state in the text that the ASO-increased AHP was sensitive to both BK and SK antagonists because the AHP consists of distinct components that could have slightly overlapping influence in our analysis window (fAHP and mAHP, 10–200 ms). For readers interested in specific effects of ASO during the fAHP and mAHP windows, we point to Table S1. Of note, when we analyzed the first derivative in the initial 20 ms after the AP peak (i.e., the presumed fAHP window), we did not observe a significant difference in the fAHP inflection time, suggesting that the ASO does not alter the fAHP.
- We have clarified the definition and analysis parameters of the sAHP in the figure and have added text describing its calculation in the Methods.
- We also added a paragraph to the Discussion addressing the limitations of ASO knockdown and acknowledging that neurons can compensate for ion channel disruptions. Importantly, the output of the neurons (sustained action potential firing) is corrected across both inhibitory (new Figure 5) and excitatory neurons.

3. Figure 1C-D: the authors found that ASO affects the characteristics of mAHP. What are the components of mAHP in KCNT1-p.R474H neurons? In hippocampal CA1 neurons, HCN, SK, Kv7 channels contribute to mAHP, while BK mainly contributes to fAHP. Why did the authors only use BK and SK antagonists to treat KCNT1-p.R474H neurons? Meanwhile, KCNT1channel directly contributes to both mAHP and sAHP, and ASO inhibition of KCNT1 could directly change the characteristics of mAHP and sAHP. The conclusion that ASO affects mAHP through BK and SK channels seems unreasonable.

- We concur with the reviewer's comment: ionic conductances underlying the mAHP and sAHP in neurons overlap between various channels, and the AHP is carried differentially by ion channel subtypes in different cell types (see also answer #2). As we did not observe significant changes in the AHP between WT control (PGP1) and R474H neurons, we turned our attention to alternative channels known to affect the AHP (channels with clean pharmacology), such as BK, SK, and Kv7. Our experiments using bath perfusion of apamin, a highly selective blocker of SK, showed that the AHP phenotype in ASO-treated cells could be blocked, while apamin had no effect on vehicle-treated or NT-ASO-treated cells. These findings led us to hypothesize that SK likely contributes to the ASO-induced increased AHP (and BK, to a lesser extent, as shown by paxilline treatment). While our initial pharmacology experiments targeted both BK and SK channels as possible explanations for the larger AHP following ASO treatment, our broader goal is to highlight that even selective ASO modulation can result in homeostatic[3] compensation with calcium-activated K^+ channels, as we have observed with other NGN2 lines. To this end, we provide patch-clamp data assessing excitability in ASO-treated R474H ENs in the presence of blockers for common K^+ channel AHP-associated proteins (Figure S4: BK, SK, KCNQ2). These data show that while BK and SK blockers attenuate the ASO effect, KCNQ2 blockers do not, further supporting our choice of targets.

- Of note, we now include data from ASO-treated patient ENs exposed to the Kv7 blocker XE991. Similar to apamin, XE991 treatment resulted in a significant reduction in the AHP. Yet unlike apamin, the excitability curve improved following XE991 application. Despite this observation, we are hesitant to make any claims regarding the role of Kv7 in ASO-induced changes in AHP, because XE991 also significantly affects the AP threshold, which is our reference potential for AHP measurements. Kv7 channels underlie the non-inactivating M-current, which affects not only the AHP but also the resting membrane potential and threshold. Therefore, while XE991 reduced the AHP to a similar degree as apamin, this effect was confounded by the XE991-induced hyperpolarized threshold shift. We included these new findings and expanded the Discussion to acknowledge that several channels could be compensating for ASO-induced AHP changes.

4. Figure 1C-G: ASO treatment appears to increase mAHP through calcium-activated potassium channels (BK and SK) in Figure 1C-G. The effects of ASO on BK and SK channels raise concerns about the specificity of ASO for KCNT1 targeting. The authors should address the mechanism of action (MOA) of the ASO treatment in greater detail, particularly its potential off-target effects.

- We added new RNA-seq data to show the full transcriptomic profile of ASO-treated ENs, including an analysis of other action potential-associated genes, all of which are unchanged following ASO treatment (Figures 1F and S1H). In the Discussion, we now include mechanistic insight into how downregulation of KCNT1 by ASO might lead to compensatory responses.
- To address potential adverse off-target effects of RNase H, we performed apoptosis (TUNEL and cleaved caspase-3) and cell health (neurite density) analyses, which demonstrate that there are no differences across treatment conditions: vehicle, NT-ASO, and ASO (new Figures S3B and S3D).
- We also now clearly point to our *Nature Medicine* paper (Nakayama et al.), which provides *in silico* modeling data.

5. Figure 2B: it is necessary to supplement the action potential burst of WT neurons to demonstrate that ASO can restore the abnormalities in neuronal excitability caused by mutations.

- To address the reviewer's concern, we provide a new data figure on the effect of ASO on WT NGN2 neurons, during minimal stimulation (new Figure S2), and during spiking (new Figure S4B).

6. The differences in ASO' effects on mAHP between primary neurons and NGN2-derived neurons requires further explanation.
- We now evaluate the effects of ASO treatment on NGN2 ENs from both p.R474H patient lines and healthy controls (PGP1), both of which show an increase in the AHP (see Figures 1C and S2D). In contrast, ASO-treated primary neurons did not show changes in the AHP following ASO treatment.
- To further explore how different cell types might respond differentially to ASO treatment, we expanded our analysis to include iGABA neurons from R474H individuals (Figures 5 and S7). We found that iGABA neuron AHPs were unaffected by ASO treatment, yet ASO treatment improved high-frequency firing (new Figures 5 and S7).
- We have added text to the Discussion highlighting that NGN2, primary, and iGABA neurons represent different cell types and developmental stages. These differences likely contribute to variations in compensation[4], as K^+ channel mutations are known to affect cell types differentially[5].

7. In vivo models would provide more evidence to confirm the efficacy of ASO for KCNT1-related epilepsy.
- We hope to follow up this work with a KCNT1 GoF mouse model to evaluate how early prenatal dosing can improve seizure onset and early death.

Minors :
1. Page 8: The article "Antisense oligonucleotide therapy for KCNT1 epilepsy of infancy with migrating focal seizures" has been cited several times in this manuscript, but it is not yet online at present.
- The clinical trial paper (Nakayama et al.) has been resubmitted following revision at *Nature Medicine* (Smith lab is a middle co-author). A copy of the manuscript can be obtained from the *Nature Communications* Senior Editor, Ekaterini Maria Lyras, who is coordinating the publication. The Nakayama et al. publication will be published shortly.

2. Figure 1B should include a schematic diagram of voltage-clamp stimulation, and Fig 1C needs to label the holding potential and current injection protocol.
- We now include these details and the holding potentials throughout the paper.

3. Figure 1G is not cited in the manuscript.
- We have removed Figure 1G and replaced it with new RNA-seq data.

4. Figure S2A: The last panel is deficient in the ordinate.
- We include new Figures S2 and S4, where we present the ordinate data comparing patient neurons to two control lines (PGP1 and KOLF); see also new data in Table S1 for comparison.
- We include new data in Figure S2 on WT neurons treated with ASO, demonstrating no effect on the outward K^+ current, while ASO still induced an AHP in WT ENs.

Reviewer #2 (There are some interesting and important results presented in this manuscript. These fall into two separate categories. The results that are presented in the second half of the manuscript (but the first part of the abstract) are potentially the most straightforward and important. Using both RNA profiling and patch clamp recordings, the authors show that KNa1.1 (KCNT1) channels are expressed in human brain in both excitatory and inhibitory neurons at mid gestation They demonstrate that suppression of these channels using an ASO reduces the ability of the immature neurons to fire at high rates, suggesting that, under normal conditions, the biological role of these channels is to promote repetitive firing. These experiments provide a firm basis for making decision about the optimal timing to begin therapeutic treatments for conditions caused by gain-of-function mutations in KCNT1. Because there is currently much work in this therapeutic area, the findings will be particularly relevant for these efforts.
The other part of the work, which is presented first in the body of the results section, is also very interesting, clearly presented and carried out rigorously. I believe, however, that it is a little more difficult to interpret, and could benefit from a clearer statement about the different possibilities. It describes the effects of the ASO that suppresses KNCT1 expression on ionic currents and firing patterns of neurons differentiated from iPSC cells of a patient with the KCNT1-pR474H mutation. The neurons were differentiated using lentiviral-mediated overexpression of NGN2. The effect of the ASO was quite different from what was found with the human neurons in primary culture. The ASO was found to have a complex effect on excitability, suppressing firing in response to small depolarizing currents but increasing firing in response to larger stimuli. This was accompanied by an increase in both medium and slow afterhyperpolarizations (mAHP and sAHP) that follow a single action potential or a train of action potentials. Pharmacological experiments indicated that these effects of the ASO were due to increases in BK and SK calcium-activated potassium currents.
The rationale for testing an ASO is that suppression of KCNT1 expression may reverse the effect of a gain-of-function mutation. It is possible, for example, that reducing the levels of KCNT1 proteins promotes the expression of other channels by a mechanism distinct from lowering KNa1.1 activity. One difficulty, therefore, in interpreting the effects of the ASO on the KCNT1-pR474H cells is that it not known if the mutation itself produces a loss of BK and SK currents. If these were the case, this would provide a powerful case for the utility of these ASOs. While the manuscript provides some data (Fig. S1G) that the ASO also increases the mAHP in a control NGN2 cell line, there is insufficient information to determine whether the properties of control cells are distinct from those of the KCNT1-pR474H cells. From the present data, therefore, it is not possible to determine if the ASO: 1) reverses the effects of the gain-of-function mutation, 2) produces changes in ion channel expression that are distinct from the effects of the increased KNa current or 3) has effects that are specific to NGN2-differentiated cells but not seen in native neurons. Some of this could be addressed by more explicit statements about these potential effects of the ASO, <u>or by providing more information on control cells.</u>

- We thank Reviewer 2 for their comments. We conducted new experiments to show the effect of the ASO on control excitatory neurons (ENs) as well as on patient-derived inhibitory neurons (INs) to assess ASO reduction in gain of function effects vs ASO on normal KCNT1. Please see below a line-by-line review of the additions to help with clarifying changes.

Specific comments:

1) The work found that treatment of human primary neurons with the ASO reduces their

excitability, suggesting that the normal role of KNa channels in these neurons is to increase their ability to fire at high rates. An increased ability to fire at high rates with increased KNa current has been found for another KCNT1 gain-of-function mutation in human iPSC-derived neurons (Quraishi et al., J. Neurosci., 39:7438, 2019) and also in cortical pyramidal neurons of mice expressing the same KCNT1 mutation studied here (Wu et al. Cell Reports 43:113904, 2024). This suggests that the work with the human primary neurons likely reflects effects on excitatory neurons, and it would be appropriate to cite both these previous publications in the manuscript.
- We have modified the Discussion to properly address these points. We also address these papers in the context of our new GABAergic data, where we observe differential effects on iGABA versus NGN2 neurons.

2) The study of cortical neurons in mice with the corresponding KCNT1-pR474H mutation (Wu et al.,) found that, in contrast to pyramidal cells, action potential firing in inhibitory neurons was suppressed by the increased KNa1.1 current. This was also found in a mouse study of a different Kcnt1 mutation that is referenced in the third paragraph on page 20 (Shore et al.). While the manuscript describes the NGN2-differentiated KCNT1-pR474H neurons as "cortical excitatory neuron", the more complex response of these cells to the ASO suggest that their phenotype may more closely resemble a hybrid of responses found in native excitatory and inhibitory neurons. Readers would be helped by an explicit clarification of the expected responses of gain-of-function and ASO suppression in different native neuronal types.
- Thank you for this suggestion. We now provide new data to better highlight the effect of ASO on WT (or non-disease) neurons during single spikes (Figure S2), as well as during high-frequency spiking (new Figure S4B).
- To further explore native neuronal types, we also provide new data on iGABA inhibitory neurons (new Figures 5 and S7), where we show GoF outward K^+ currents and the effect of ASO on GABAergic neurons, resulting in no effect on AHP yet improved high-frequency firing.
- We added text to the Discussion, including potential differences between human cells and mouse models in KCNT1 expression.

3) Text in the middle of the abstract of this manuscript makes the statement that the ASO reduced seizure occurrence and severity. These results are not presented in this manuscript, but instead are described in a different manuscript that is related to the present work and is provided as supporting information. This information should not be placed in the "results" section of the abstract itself. In addition, the statement about the effects of the ASO on one of the patients (2nd paragraph, page 19) is unclear to the reader in the absence of this unpublished information.

- We added a clear citation to the abstract for this referenced work, positioning it early in the text before the results. The clinical trial paper has been resubmitted following revision at *Nature Medicine* (we are middle co-authors). A copy of the manuscript can be obtained from the *Nature Communications* Senior Editor, Ekaterini Maria Lyras, who is coordinating the publication.

Minor comments:

- A clarification is required for how the measurements of KCNT1 mRNA were quantified for Supplemental figure 1B. The legend state that gene expression levels in vehicle-treated cells were adjusted to one, but the figure itself shows a fold change of less than one for vehicle treatment in control.

- We regret this was not made clear and have clarified the figure and methods. To further support the qRT-PCR data, we now show new RNA-seq data (Figure 1F, S1H, Tables S5–S6). These new data confirm previous PCR findings, demonstrating that KCNT1 is selectively targeted without any off-target changes in gene expression.

- The text at the top of page 10 refers to figure S1D for the effect of the ASO on the mAHP in an cell line from a healthy individual. Presumably this is meant to be figure S1G.
- Thank you for pointing this out. We removed this finding and added new data in Figures S2 and S4, highlighting the ASO effects on control cells with normal KCNT1.

-It is stated that on page 10 that nifedipine did not affect mAHP amplitude in ASO-treated neurons. The related figure (S1H), however, shows that the AHP is severely disrupted by nifedipine. The fact that the membrane potential returns to baseline within 200 msec of the end of the spike does not fully justify the statement that the mAHP is unaffected.
- In our original submission, we attempted to keep all the AHP analyses within the same time window. We now include new evidence of disrupted AHP kinetics in nifedipine-treated cells and show an analysis of the AHP area, reflecting the current with respect to the baseline, to highlight the different rate at which nifedipine-treated cells recover to baseline (Figure S2F). This AHP area is significantly different from cells not treated with nifedipine, as shown in the trace. Additionally, we provide pharmacologal data (including nifedipine) for ASO-treated ENs and high-frequency firing (Figure S4D).

Reviewer #3 (Remarks to the Author):
This manuscript by Golinski et al evaluates the potential of antisense oligonucleotides (ASOs) for prenatal enriched potassium channel associated epilepsy. While this paper offers interesting insights into potential prenatal and early infantile ASO-mediated therapeutic options for EIMFS, there are both major and minor concerns that should be considered as listed below:

- We thank Reviewer 3 for their comments. We performed new experiments to address concerns and added text for clarity throughout the manuscript. Please see below a line-by-line review of the additions to help with clarifying changes.

Recommendation: Major revisions

This manuscript by Golinski et al evaluates the potential of antisense oligonucleotides (ASOs) for prenatal enriched potassium channel associated epilepsy. While this paper offers interesting insights into potential prenatal and early infantile ASO-mediated therapeutic options for EIMFS, there are both major and minor concerns that should be considered as listed below:

Major concerns
1. Many NGN2 neuron Ephys + morphology results in Figure 1 seem to be similar to experiments performed in Nature Medicine paper that was given to reviewers as related materials (Fig 2). Were these experiments performed by a different lab than what is reported in the Nature Medicine manuscript? Were they at least independent differentiations? Much of this Nature Communications manuscript seems like it is supplemental work that did not make it into the Nature Medicine manuscript and instead was put together in a new paper. There is nothing wrong with that, BUT, it should be very clear to the reviewers and the readers if this is "extra" data from the same set of experiments detailed in the Nature Medicine work, or if these are independent experiments. If they are not independent experiments, parts of this manuscript could be misconstrued as replication of the Nature Medicine findings, when in fact, it is not.
- We apologize for the lack of clarity on the differences between this submission and the clinical manuscript. All patch-clamp data are new for this manuscript. The experiments were designed differently, including longer tissue culture dosing in more mature cells, different cell types, pharmacological isolation, cell viability assays, complex spiking analyses, AHP window analysis, experiments at resting V_m and -60 mV, as well as different experimental parameters used for stimulating cells. The only experiment we replicated to demonstrate reproducibility and reliability of the voltage-clamp studies is the ASO NGN2 EN outward current knockdown (Figure 1B).

2. The authors refer to p.R474H neurons as mature at 14 days in culture, whereas most of the field would likely argue maturity is not reached until Days 21-28 at the very earliest (PMID: 29791859; PMID: 39255791). Typically, this is defined by the presence of active synapses and ephys network activities. The authors do not show neuronal activity until Day 28, so I suggest they remove the verbiage saying neurons are mature at 14 days OR show immunostaining and/or ephys to demonstrate functional maturation.
- We agree with the conclusions of the two papers above and regret the lack of clarity in our choice of "mature" vs. "maturing." Previous studies of KCNT1 were performed at this early stage, and it was our intention to highlight that we do not patch NGN2 neurons until approximately day 28 post-differentiation, by which point we expect near full maturation. To clarify this, we have revised the wording, modified the schematics accordingly (see Figure 1A, top), and replaced the term

“maturation” with “days post-differentiation.” Our NGN2 protocol is based on Nehme et al., 2018[6]. Critically, as this study focused on intrinsic excitability, we did not assess synaptic features.

3. While the authors have assessed ASO induced changes to neuronal morphology, I am wondering if the authors have tested for other toxic factors such as cell viability? If so, please provide that data along This is important given that the patients described in the Nature Medicine had side effects that could potentially be a result of neuron death (e.g. enlarged ventricles).
- We now include Click-iT EDU staining (apoptosis) and cleaved caspase-3 staining of vehicle-, ASO-, and NT-ASO-dosed ENs. We did not observe increased signal for apoptosis between these conditions (new Figure S3).
- We also added a new cell type in the neuromorphological analysis: ASO-treated iGABA neurons. Analysis of neurites in these iGABA INs (new Figure 5E) revealed no gross neuromorphological differences following ASO treatment.
- In new RNA-seq data, we found no transcriptomic enrichment for cell stress or death pathways in NGN2 ENs following ASO treatment (Figure S1H, Tables S6).

4. In Figure 1B, the authors compare WT, Veh, Patient+NT-ASO, and Patient+ASO neurons for all assays. They present p-values that denote differences between Patient+NT-ASO, and Patient+ASO. It is unclear if there are any statistical differences between the WT control and the Patient+NT-ASO lines. If there are no differences, one could ask what exact phenotype are the authors trying to rescue? Along these same lines, the authors do not include the WT control in any of the subsequent assays. Again, leading one to ask, is the ASO rescuing these phenotypes to WT levels if there are indeed real differences that need to be corrected.
- We found no statistical difference in outward steady-state currents between ASO-treated R474H ENs and untreated WT ENs. This finding suggests that the GoF observed in R474H neurons is quite large compared to the resting KCNT1 in ENs. To this end, we performed new experiments where we applied ASO to WT neurons, showing that the ASO is not able to significantly reduce outward currents (new Figure S2C) and resulted in only small improvements in AP spiking (new Figure S4C).

5. Even though no observable changes are seen in MAP2, GFAP, and Tuj1 in Figure 4A, but there is no quantification to support this claim. Even pixel signal intensity is sufficient to make this assertion, but representative images is not adequate.
- To address this concern, we performed blinded neurite density analysis using a MAP2 marker across conditions (Figure S2) and demonstrated no differences between treatment conditions—vehicle, NT-ASO, and ASO—in MAP2 neurite signals (new Figure S3B).

6. It is fundamentally unclear what the authors’ take home message is regarding the utility of the NGN2 models described early in the paper when the later experiments in primary neurons give the impression that the NGN2 model is not valid. The authors should make it clear if the NGN2 data is valuable to their conclusions, otherwise this data should be be removed. Alternatively, if the authors feel the need to make claims about NGN2 vs primary models, they should either do side-by-side comparisons or at the very least present these two models in the same section of the paper.
- We apologize for the lack of clarity in interpreting the NGN2 neuron data in the context of disease modeling. To this end, we have revised the text to tone down the limitations of any one cell type

and to highlight the utility of each type. As our fetal neurons likely represent an earlier time point than NGN2 iNeurons, both neuron types offer utility in interpreting how ASOs might result in different response profiles across cell types and developmental stages (most critically, how an ASO would perform in a preterm infant vs. a one-year-old). We also include new iGABA neuron data (Figure 5), which show ASO responses in a different cell type, highlighting both shared and divergent ASO response profiles compared to NGN2 ENs. We also add new data for control WT NGN2 iNeurons to show how ASOs modulate WT control cells (new Figure S2). We expanded the discussion content on mouse model KCNT1 cell-type-specific pathophysiology and how K^+ channels differentially affect different cell types[5,7–9] .

7. The quinidine experiments seemed uninformative, incomplete, and seemingly out of nowhere. Please either explain why these experiments are relevant by giving more context behind the motivation for these experiments in the grand scheme of the project's goals or cut them out entirely.
- We regret that the description of the rationale for our quinidine experiments was not clear. As quinidine is commonly prescribed to DEE14 children, we thought it would be useful to demonstrate the pharmacological effects of this small molecule on fetal cells, for infants born preterm with seizures. Nevertheless, upon further discussion with the editor, we have removed the quinidine data from the paper to keep the focus on ASOs. We hope to make these data available in a follow-up study for clinicians.

8. What is the purpose of Figure 5? It shows the KCNY1 prenatal localization and postnatal and ASO. This figure is not discussed at all, and there is no legend. Was this included by mistake?
- We apologize for the confusion; Figure 5 was not intended to be a data figure, but rather a graphical abstract to rapidly and visually represent the paper's findings to the broad *Nature Communication* readership. This graph is no longer included with the primary data figures. We now present a new Figure 5 (induced INs and ASO treatment.)

Minor:
1. At some places, the figure call outs in the text do not match the actual figure panel. For example, the authors write - "However, the KCNT1 signal was negative in glial fibrillary acidic protein (GFAP) cells throughout the deep to superficial cortex (Figure 3B)". But, figure 3B does not contain any GFAP panel. Please double check figure panels and text.
- We apologize for this oversight. The manuscript has been corrected to clarify that GFAP staining is part of Figure S5B.

2. Figure panels should be mentioned in the text in the same order they are presented in the figure. For example, in Figure 1, Rheobase, mAHP, and threshold are discussed in this order in the text but not in the figure itself. This is also true for Figure 3C which is discussed after Figure 3E.
- For most of the analysis in Figure 1, the rheobase stimulation protocol (1–2 pA steps) was used to evoke a minimal AHP and threshold. Therefore, the rheobase, AHP, and threshold data (presented as average action potentials across all cells) in Figure 1C, as well as the values presented as bar graphs in Figure 1D, follow this stimulation. We hope this clarifies why rheobase is mentioned first.
- As per the reviewer's suggestion, we have reformatted Figure 3 to reflect the flow of the results text.

3. Again, there needs to be more consistency between figures and text. Eg, Fig. 1B shows WT-Neurons, but the results text only refers to this PGP-1 cell line. This can create confusion for the reader. The authors refer to apamin as APN in the text, but in Figure 1E it is referred to as Apa. Please double check figure panels and text for consistency.
- We consider the PGP1 cell line to be a WT line. In Figure 1E, we included "wild-type" (in lieu of PGP1 or "control"), as *Nature Communications* readers might not be familiar with the name PGP1 or might interpret "control" as referring to baseline p.R474H. We now clearly state in the figure legend that the wild-type line is PGP1.
- The APN acronym is now consistently presented throughout the text and figures. We apologize for the earlier inclusion of the APA abbreviation (another common acronym for apamin).

4. Some figures have no reference at all in the text (e.g. Figure 1G). Please provide context for all figures included in the panels.
- We apologize for this oversight. Panel 1G has been removed and replaced with new data.

5. Although the statistical section in the methods says that "Figure legends include details of the statistical methods used for analysis", the figure legends just state the p-value but not the actual test carried out for specific figures. Please include information of the statistical testing carried out for each figure in the figure legends.
- Thank you. We have added the missing text to the figure legend.
- We also report relevant p-values within the figures wherever possible to help readers interpret levels of significance.
- All values can be found in Tables 1–4.

6. Figure 3A shows KCNT1 expression up to 15 yrs of age, which they refer to as adulthood. It seems the more correct term should be juvenile/teenage/adolescent.
- We apologize for this oversight; we now refer to this late time point as "late-adolescent."

7. Many parts of this paper are written in a way that is only accessible by experts in ephys. Nature Comms is a broad audience journal, so this needs to be addressed, especially when it comes to explanations of experimental design and interpretation. For example, "Moreover, the KCNQ2 channel antagonist XE991 did not reduce AP excitability in ASO-treated neurons, but instead increased high-frequency firing." The authors do not mention what KCNQ2 is and why exactly the use of its antagonist would yield this conclusion. In addition, see are above comment about the quinidine experiments and how they were not sufficiently described.
- We have improved the descriptions of the patch-clamp terminology.

8. Grammar mistake: "Patient-derived iPSCs use NGN2 induction to generate a cortical-like excitatory neuron…". The iPSCs do not use NGN2 induction, the NGN2 induction is performed on the iPSCs.
- We regret this oversight and have corrected it.

Reviewer #4 (Remarks to the Author):

Golinski et al present a study entitled: Gene therapy for targeting a prenatally enriched potassium channel associated with severe childhood epilepsy and premature death. The authors report the electrophysiological onset of KNa1.1 in human brain development and the cellular basis for a first-in-human application of an ASO targeting KCNT1-associated EIMFS. The manuscript is clearly written and the data presentation is appropriate. The reviewer has a few comments and concerns.
- Thank you Reviewer 4 for the comments and concerns. We performed new experiments to address concerns and added text for clarity throughout the manuscript. Please see below a line-by-line review of the additions to help with clarifying changes.

Gene therapy is mentioned in the title. The reviewer wouldn't consider an ASO as gene therapy.
- We have revised the title to say, "RNA targeting therapy."

P3. According to the most recent classification by the ILAE, epileptic encephalopathy should be renamed as developmental and epileptic encephalopathy.
- We have modified this to reflect the recent classification: "DEVELOPMENTAL AND EPILEPTIC ENCEPHALOPATHY 14; DEE14."

The abstract may be a bit misleading since the authors are mentioning the results of a clinical trial that is not a part of this study. If the results are relevant to mention in the abstract, then specify that it was a previously conducted study.
- We apologize for the confusion; our intention was to cite the clinical paper by Nakayama et al., which is expected to be published within the next two months. We have updated the text accordingly.

In the introduction the authors are referring to a Nature Medicine paper describing the results of a clinical trial with two children with KCNT1-related EIMFS, who were successfully treated with an ASO. The reviewer can't find that reference, has the paper been published? The results of a small ASO trial with two children with KCNT1 has previously been mentioned in New York Times. In that study both children developed severe hydrocephalus, and one child died because of this adverse event. Are the authors referring to the same study?, if yes then maybe this should be mentioned.
- The clinical paper describing these two children is currently under revision at *Nature Medicine* (Nakayama et al.). The latest copy of the manuscript can be provided by the *Nature Communications* Senior Editor, Ekaterini Maria Lyras, PhD. In that paper, clinical profiles, seizure features, and ventricular/immune response monitoring are extensively discussed.
- We have added new text to our discussion to highlight concerns about hydrocephalus and to describe the new cell death/stress analyses we have performed to evaluate ASO effects on tissue culture health (new Figure S3).

The authors are mentioning that half of KCNT1 EIMFS kids do not survive past the age of 3 years. The reviewer agree that there is an increased mortality in this patient population, but according to a large study of more than 150 KCNT1 EIMFS cases the mortality rate is not as high as 50% (PMID: 34114611).
- We have adjusted the text throughout the manuscript to reflect the increased mortality rates reported in this larger study.

It is unclear if CNV analysis has been performed in the p.(R474H) hIPSCs, and whether isogenic controls have been generated.
- We now include karyotype analysis in Figure S3G.
- The p.R474H variant has been broadly documented for over 10 years as a highly penetrant monogenic disorder that results in GoF pathology across multiple children. The ASO reduced seizures in two children from different families. Therefore, we sought to therapeutically profile the ASO across several cell types, including WT control lines, to show the effect of the ASO on both patient and normal neurophysiology.
- In a time of limited resources, we find that highly penetrant, known monogenic disease variants are sufficient to drive key advances in precision therapeutics—both rapidly and in depth. Nevertheless, we recognize the value of CRISPR isogenic controls for other diseases we study in the lab (e.g., iPSCs from childhood-onset schizophrenia cases), where polygenic and genetic background considerations play a greater role, requiring multiple isogenic lines to study effects. After considering these factors, we decided not to pursue an isogenic CRISPR knock-in approach to correct the allele or to generate a new line from a control background.

The obtained results suggests that ASO targeting of perinatal KNa1.1 GoF current might help limit disease progression compared with postnatal treatment. Do the authors have any suggestions on how these children can be diagnosed prenatally?
- While we suggest perinatal treatments, a key takeaway remains the importance of timely perinatal intervention due to likely prenatal pathologies. We have added new text in the Discussion to highlight non-invasive prenatal testing and targeted prenatal testing. Notably, just this past month, the first prenatally identified neurodevelopmental disease was described (Finkel et al., 2025, *NEJM*)[10]. This pathology was subsequently treated *in utero*, and the child exhibited no major symptoms postnatally, supporting our enthusiasm for perinatal therapeutics.
- To this end, as preterm infants increasingly undergo rapid next-generation sequencing with clinical exome analysis at birth, this enables timely and targeted interventions.
- We have added text to the Discussion to address the therapeutic potential and the current state of the field.

Reviewer #5 (Remarks to the Author):

KCNT1-linked early infantile developmental and epileptic encephalopathy (EIEE14, EIFMS) is a devastating disease caused by gain-of-functon(GoF) de novo variants in KCNT1, encoding KNa1.1, resulting in increased outward K+ currents and reduced excitability of cortical GABAergic interneurons, disrupted E:I balance, and overall network hyperexcitability. Here, iPSC NGN2-differentiated cortical-like excitatory neurons generated from a patient with a GoF KCNT1 variant, p.R474H, showed blunted excitability including abnormal action potential (AP) AHP kinetics, such that neurons cannot maintain AP firing at high current injections. The authors investigate the mechanism of action of a recently developed, first-in-human, KCNT1-targeted RNAse-H activating ASO that previously showed significant reductions in seizure frequency and severity in two EIEE14 patients. They show ASO efficacy and specificity in lowering outward K+ currents in both patient-derived iPSC NGN2 neurons and primary human fetal neurons from mid-gestation neocortex. Because KCNT1 mRNA is shown to be expressed during mid-gestation brain development in humans, the authors make a case for early therapeutic intervention with prenatal ASO delivery, prior to full disease manifestation.

- We thank Reviewer 5 for their comments. We performed new experiments to address concerns and added text for clarity throughout the manuscript. Please see below a line-by-line review of the additions to help with clarifying changes.

1. For completeness, the authors should include the OMIM term for KCNT1 linked early infantile developmental and epileptic encephalopathy, DEE14.
- We now include "DEE14" throughout.

2. The authors do not indicate how many iPSC lines were generated from the patient samples..
- We have validated the ASO on one patient iPSC line (p.R474H).
- For reasons explained in our response to Reviewer #4 above, we sought to deeply profile the ASO across several developmental cell types (iNeuron, iGABA-neuron, primary neurons) and a control line to show the effect of the ASO on both patient and normal neurophysiology.
- Importantly, a central emphasis of our paper is the non-allele-specific nature of this knockdown (and therefore its potential for broad use across other GoF KCNT1 patients with other alleles, such as P924L) and that the ASO can be used to dose other cells and lines. Therefore, our inclusion of PGP1 control lines, iGABA neurons, and primary human control neurons demonstrates the ASO effect on various cell lines, as opposed to focusing on several clones from one differentiation strategy.
- Each differentiation was an independent NGN2 transduction (a different viral transduction on a different day), with a minimum of three differentiations for NGN2 neurons. We also performed three differentiations for iGABA neurons using the PiggyBac iGABA approach (see numbers reported in the Methods).

3. NGN2 differentiation skips over critical neuronal developmental time points. Because a goal of this work is to model an early infantile, possibly fetal, brain disorder, NGN2 differentiation may not be the most appropriate method. In contrast, dual SMAD differentiation, which more accurately models early brain development, would be more appropriate. This differentiation issue may explain why ASO treatment of fetal neurons did not recapitulate the NGN2 neuron response of increased mAHP – although being able to show this result in more than one iPSC cell line, as discussed above, may also be a factor.
- For NGN2 differentiations, we combined dual SMAD inhibition—an approach we adopted from Nehme and colleagues—to improve circuit physiology[6]. We now clarify this in the figures and Methods.
- We now present new data with control NGN2 ENs (Figures S2 and S4), as well as iGABA IN differentiations, to support that the ASO results in different AHP effects across cell types. We add text to the Discussion regarding ASO cell type-specific effects.

4. What is the rationale for the use of iPSC pyramidal neurons for this study? How do the authors propose that expression of a GoF KCNT1 variant in cortical pyramidal neurons, resulting in reduced intrinsic excitability, contributes to epileptogenesis? Previous work in mice showed predominant KCNT1 expression in PV+ and SOM+ interneurons, with near-absence in other interneuron subsets (e.g. VIP+), with intermediate expression in excitatory pyramidal neurons. Others have shown that GoF KCNT1 variants linked to DEE14 significantly impact inhibitory interneuron excitability and disrupt E:I balance. Thus, this study would have greater impact if the authors included a study of inhibitory iPSC neurons in the presence and absence of ASO. While

PV+ interneurons are difficult to generate in 2-D culture, many groups have successfully generated SOM+ interneurons.

- We now add iGABA differentiations (Figures 5 and S7) and better highlight the cell type-specific ASO effects in iGABA vs. NGN2 vs. primary neurons.
- Regarding INs vs. ENs *in vivo*, a key question remains: even if KCNT1 has considerably lower expression in ENs compared to INs, a GoF mutation of large effect can still greatly destabilize EN physiology. Therefore, while ENs may have a decreased firing rate compared to the faster-spiking INs, the GoF effects might be even more poorly buffered by the intrinsic properties of ENs.
- Regarding the choice of iPSC-derived pyramidal neurons, a key component of our study is the developmental aspect. Our data clearly show that *KCNT1* is present in early pyramidal neurons in the fetal and infant cortex, and NGN2 cells are the most suitable differentiation strategy for these neurons.
- Lastly, regarding differences in mice and human cortical connectivity, developmental timing, and differential KCNT1 expression profiles across interneuron subtypes: see the graph of KCNT1 expression in inhibitory cell types from the neocortex (right panel), where we analyzed RNA data from various species based on Krienen et al., 2020[11]. These findings highlight the difficulty in mapping *in vivo* and *in vitro* models across species for rodent-based preclinical therapeutics. We have added text to the Discussion to address this.

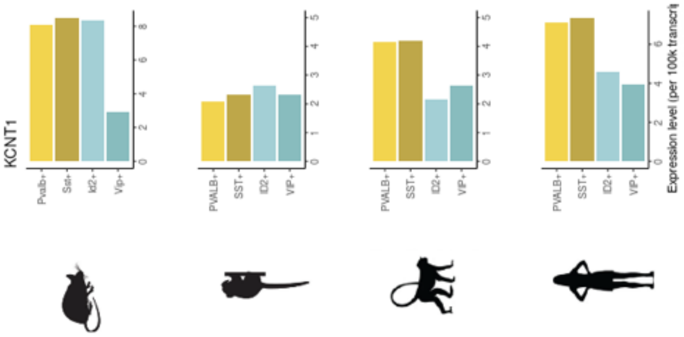

5. What are the effects of ASO treatment on synaptic activity between patient neurons, especially on restoring inhibitory tone? Is the synaptic activity of control neurons altered by ASO treatment?

- As our focus is on early development—when the prenatal brain has fewer synapses—we limited the scope of this study to intrinsic excitability and the ASO effects on individual neuron types, cell morphology changes, and developmental implications.
- We did not explore synaptic tone in ENs and INs, and we now comment on this limitation in the Discussion. We are enthusiastic about follow-up studies involving mixed cultures of pathogenic ENs and INs to evaluate synaptic activity and E:I ratios.

6. Does ASO treatment of iPSC or human neocortical neurons in culture impact/delay the maturation of GABAergic signaling from excitatory to inhibitory?

- For ASO treatments, we typically wait until intrinsic excitability is nearly fully established. In our hands, neurons stabilize in AP firing kinetics/waveform (after ~16 days for NGN2 and ~30 days for iGABA) before ASO treatment. Of note, previous analyses of NGN2 neurons were conducted before 20 days *in vitro*. A key distinction in our protocol is ensuring that our neurons are more mature before treatment.
- Additionally, our new RNA-seq data comparing ASO-treated NGN2 ENs indicate a comparable transcriptomic profile (Figures 1H, S1H; Tables S5–S6). If ASO treatment were significantly altering cell state, we would expect this to be reflected in the RNA-seq data.
- Regarding effects on GABAergic neurons, our new iGABA ASO experiments show limited changes in AP waveforms (new Figure S7D) following ASO treatment, yet we observe reduced high-frequency firing of iGABA INs.

7. What is the specificity of the ASO for reducing KCNT1 mRNA vs. other K+ channel mRNAs and Na+ channel mRNAs?
- Thank you for the opportunity to clarify this important point regarding the selectivity of our ASO. We hope our new data clarify that the lead ASO selectively reduces only the KCNT1 portion of the outward K^+ current, leaving other K^+ currents unaffected. We also now add a reference to the clinical manuscript "Nakayama et al., in revision" with *Nature Medicine*, which details *in silico* modeling and selection of a highly selective ASO from over 100 compounds to one lead compound.
- We added new RNA-seq data to demonstrate the ASO's effects: KCNT1 is the only ion channel gene significantly changed (new Figure 1F, Table S5). See the complete gene list in Tables S6.
- In addition to RNA-seq data, we provide new quantification of Na^+ current levels between ASO-treated cells and conditions: NGN2, PGP1, and iGABA (Figures S1D, S2C, S7C).

8. Page 10 – Figure 1G does not appear to be discussed. This section lacks a concluding statement.
- We have removed the schematic from Figure 1G and replaced it with new RNA-seq data.

9. Does the expression pattern of KNa1.1 protein follow the KCNT1 mRNA expression pattern shown for human prenatal cortical neurons? With recent new knowledge regarding the alternative splicing of ion channel mRNAs, including the inclusion of nonsense-mediated decay/poison exons, this is an important question.
- To ensure we are assessing functional KNa1.1 (not just RNA expression), we performed patch-clamp analysis of prenatal primary neurons (Figure 4C) and found a reduction in sodium-subtracted K_{Na} outward currents. We also used our highly selective ASO to demonstrate KCNT1 activity in primary fetal neurons. These data suggest that KNa1.1 protein is being produced at significant levels and is functional at this early mid-gestation developmental stage.
- To further evaluate the alternative splicing of *KCNT1* at the transcript level, we provide new targeted long-read sequencing data at four points during brain development (Figure 3C; four time points: fetal at 23 PCW, 1 month old, 3 years old, and adult). These results indicate the rare inclusion of poison exons at all time points examined, suggesting that they are unlikely to substantially contribute to the regulation of *KCNT1* transcript abundance, and that other gene regulation mechanisms likely account for the developmental increase in KCNT1. We have added these findings to the text, Methods, new Figure 3D, and Figure S5.

10. Fig. 3C appears out of order in the text of the results section.
- Thank you for this suggestion. We have reordered the results and now present the infant/toddler KCNT1 expression localization in neocortical inhibitory and excitatory neurons as part of Figure 5A.

11. What is the effect of quinidine on patient iPSC neurons?
- Upon further discussion with the editor, we removed the quinidine data from the manuscript to focus the paper on RNA mechanisms of knockdown. In brief, quinidine drastically reduces outward K^+ currents in every cell line we have tested—control and disease hiPSC neurons, and primary neurons—likely due to its non-selective nature, which affects channels beyond KCNT1. See also our response to Reviewer #3, Point 7.

Minor: The red, pink, magenta color scheme is sometimes difficult to discern, and the light pink traces want to fade into the background. I would use a more dynamic color scheme

- The color scheme was recommended by the journal guidelines (red for pathogenic cells, and shades of red for related conditions, e.g., pink and purple).
- For consistency, non-pathogenic control hiPSC lines (KOLF, PGP1) or primary neurons are shown in shades of black/gray.
- For clarity throughout, only pathogenic neurons are displayed in shades of red.

Reviewer #6 (Remarks to the Author):

I co-reviewed this manuscript with one of the reviewers who provided the listed reports. This is part of the Nature Communications initiative to facilitate training in peer review and to provide appropriate recognition for Early Career Researchers who co-review manuscripts.

- Thank you Reviewer 6 for your comments. As a new PI, these review opportunities are very helpful.

1 Colasante, G. *et al. Cell Stem Cell* 17, 719–734 (2015)

2 Andrade, R. *et al. Front. Cell. Neurosci.* 6, 47 (2012)

3 Simkin, D. *et al. eLife* 10, e64434 (2021)

4 Marder, E. *et al. Nat. Rev. Neurosci.* 7, 563–574 (2006)

5 Koch, N. A. *et al. Front. Neurol.* 14, 1194811 (2023)

6 Nehme, R. *et al. Cell Rep.* 23, 2509–2523 (2018)

7 Wu, J. *et al. Cell Rep.* 43, 113904 (2024)

8 Shore, A. N. *et al.* (2024)

9 Shore, A. N. *et al. Cell Rep.* 33, 108303 (2020)

10 Finkel, R. S. *et al. N. Engl. J. Med.* 392, 1138–1140 (2025)

11 Krienen, F. M. *et al. Nature* 586, 262–269 (2020)

Reviewer #1
Thank you for your careful review and comments to improve the manuscript

Reviewer #2 (Remarks to the Author):

The authors have added a substantial amount of new data to this manuscript. As presented, however, the overall message remains confusing. Specifically, the effects of a KCNT1 ASO have now been tested on three different cell types, and these effects of the ASO are different for each cell type. Superficially, this could lead one to conclude that such effects on human neurons are variable and unpredictable and would do a disservice to the development of these reagents for new therapeutic approaches. Nevertheless, the observed findings do make some sense in the context of what is currently known about the role of KCNT1 channels in different types of cortical neurons. The manuscript could therefore be improved by some careful rewriting, and by coupling its conclusions to what is already known of the role of KCNT1 channels in excitatory and inhibitory neurons.

Specific comments:

1) Probably the most important contribution of this study is the work with primary human neurons and the characterization of the prenatal emergence of KNa1.1 currents. Application of the ASO to these neurons results in a marked suppression of current-induced firing. These data are shown in figure 4E but (surprisingly!) are not mentioned at all in the results section.

Thank you for your attention to this important point of KCNT1 functions in native fetal primary neurons. We have now added explicit Results text describing ASO-mediated KCNT1 knockdown in primary fetal neurons and the associated suppression of current induced repetitive firing (Fig. 4E), and we added a summary sentence in the Abstract,In Brief to reflect this directionality. We also expanded the Discussion to explicitly contrast the primary-neuron phenotype with the induced neuron models.

2) The major stated conclusion of the manuscript is that ASO treatment increases action potential firing in neurons. This is stated in the “in brief” section, the abstract and in the first paragraph of the discussion (“increased action potential burst firing”, “increased high-frequency spiking and burst properties” and “increase the maximal response for input-output excitability curves”, respectively.) This is misleading because the effect of the ASO on human primary neurons was exactly the opposite; As stated above, in these neurons their major effect was to strongly suppress excitability.

We revised the Abstract and discussion to state that ASO effects are **cell-type dependent**: suppressing excitability in primary fetal neurons (a similar effect to low current stimulations NGN2 ENs), while increasing AP firing at high current stim in

iGABA and in NGN2 ENs. We edited the abstract and discussion paragraph accordingly.

3) Previous work from several groups with mouse models has shown that gain-of-function KCNT1 mutations suppress excitability of cortical inhibitory interneurons. The effect on excitatory pyramidal neurons is different in that, depending on the mutation, the gain-of-function either increases excitability or has neutral effects. The finding in the present work that the ASO increases the excitability of the IN-type cells and suppresses that of the primary cortical neurons, which are most likely excitatory cells, is fully consistent with this. The finding that the ASO also increases maximal firing rate of the EN-type cells appears, at first sight, to be inconsistent with this. But, as shown in this study, the ASO does suppress firing of the EN-type cells in response to lower current stimuli, consistent with both the primary neurons in this study and all the mouse work. The anomalous effect of the ASO on firing with stronger stimulation of the EN-cells may perhaps be explained by the uncertain nature of these cells (see comment 4).

We add new text to discussion to further highlight this, clarifying that ASO treated ENs at lower stimulation more similarly reflect classic models of KCNT1 contributions to excitability.

4) The first three pages of the results section focus heavily on the effect of the ASO to induce an increase in a calcium-dependent afterhyperpolarization in NGN2-differentiated, putative excitatory neurons. This was not observed in either of the other two cell types. While pharmacological studies provided some clues, the nature and mechanism of this effect were not established. It remains a possibility, therefore, that this is a peculiarity of this cell differentiation protocol, and may not be relevant to the actions of ASO in normal neurons. While these findings may be worth reporting, it does not make sense to make this a major focus of the manuscript. It could potentially be put in supplemental data with appropriate caveats about significance.

We have modified main figure title and results to better highlight the focus on the ASO effect on the output of the cells. For example, see Figure 1 and text, changed from *“ASO-treated KCNT1-p.R474H neurons display large AHPs sensitive to Ca2+-activated BK and SK channel blockers*” to “*ASO-treated KCNT1-p.R474H neurons display large AHPs*”. We also add text to discussion to highlight limitations that *in vitro* models might have for interpreting differences in the context of compensations.

5) In the first section of the results (p. 8-9) it is reported that the KCNT1 ASO reduced K+ current in KCNT1-p,R474H ENs but did not reduce K+ current in control PGP-1 ENs. This is in direct contrast to the effects of the ASO on primary human neurons, which, as stated above, are most likely excitatory neurons and in which the ASO suppressed K+ current despite the lack of channel mutation. A comparison of figures S1B and S2B shows, however, that there was much less knockdown of KCNT1 in the control PGP-1 ENs (S2B) compared to that levels of KCNT1 mRNA in one of the control

samples appear identical to the mutant cells (S1B), such that in the untreated cells. This seems a likely explanation for the lack of effect in the control cells, as work from numerous groups indicates that KNa currents are present in control excitatory neurons (although it is also likely that the ENs are not representative of native neurons). This should be addressed, either experimentally or in discussion.

We have added discussion and results text noting the modest reduction in K+ current observed in PGP control neurons compared to the greater degree of KCNT1 GoF mutant transcript suppression in patient neurons, which would be expected to produce a larger proportional decrease in outward K+ current, accounting for the clearer effect in the mutant cells. More specifically, regarding ASO knock down of RNA levels in Figures S1B and S2B, we note that the reduction in KCNT1 mRNA in control neurons approached near significance (P = 0.06) by qRT-PCR, yet did not approach the highly significant reduction observed in the patient neurons of P=0.004. Nonetheless, to address the KCNT1 RNA knockdown and potential compensations, we included new RNA-seq data in Figure 1F as part of the revision, which more clearly demonstrate KCNT1-selective knockdown in ASO-treated control neurons (not patient). Together, these data support the interpretation that the smaller transcript knockdown in control NGN2 ENs yields a correspondingly smaller reduction in outward K+ current, whereas the larger knockdown in the GoF line produces a clearer electrophysiologic effect.

Moreover, while NGN2-derived excitatory neurons are expected to be relatively “homogeneous” in response kinetics, as noted by reviewer, we observed variability in ASO responsiveness both within the same cell lines differentiations and between cell lines. This underscores the importance of using independent control cell lines, including the well-characterized control line for comparison (PGP1). We have added text to the *Discussion* emphasizing that ASO dosing requirements may differ between cell lines, and by extension, between individuals. We also expanded the *Limitations* section to highlight that even among the refined neuronal cell types examined (DEE versus control NGN2 neurons), we noted considerable phenotypic variability that may inform future precision-medicine approaches.

6) Previous work has shown that Na channel expression can be regulated by KCNT1. The results section (p.8) states that levels of inward sodium current density in KCNT1-p,R474H ENs were not altered by the ASO. Nevertheless, figure S1D shows that the amplitude of sodium current varies over an order of magnitude in different cells, making it difficult to make a firm statement on this. It **would be helpful to know if there is similar variability in the K+ current (The error bars in the IV plot of figure 1B suggest there is not, but individual values are not shown in bar graph form).** It might also be helpful to provide Na^+ current quantification for the human fetal neurons in response to the ASO.

The variability in the maximum sodium current shown in S1D is comparable to the variability in the potassium current shown in Figure 1B. The discrepancy you point out is a result of how the data are presented in the figures. In S1D, the data are shown as a boxplot since we are only presenting the maximum value. Conventionally, boxplots are presented as median +/- IQR, with whiskers spanning the range of the data excluding outliers. Conversely, the data shown in Figure 1B are over a range of membrane potentials, and are thus a line graph presented as mean +/- SEM. For these data, the SEM is smaller than the IQR, giving the appearance of less variability in the potassium currents than the sodium currents even though they're comparable. We represent Max K in this alternative format above for comparison.

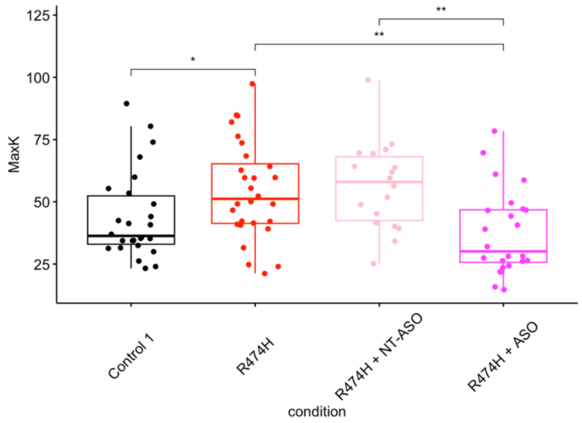

We now highlight Na^+ current quantification in the human fetal neurons in response to the ASO, comparing knockdown data (Figure S6C) in main result section, with max sodium current density and half-activation potentials($V_{1/2}$) not significantly different from control.

Minor comments:

7) How do the authors validate that their electrophysiological recordings were performed on specific neuronal subtypes? For example, although Figure 5B shows robust GABA staining via immunohistochemistry, it is not clear how they ensured that the recorded cells were indeed GABAergic neurons, as there appear to be numerous cells that are negative for anti-GABA .
-Thank you for your attention to this, in the selected 5B IHC image, 100% of MAP2+ cells (neuronal marker) are also high expressers for GABA signal. We add new text starting our iGABA protocol produced > 90% GABA+ signal in MAP2+ cells. Therefore, with our specific patch clamp inclusion criteria for mature neuron neurophysiology (robust sodium and potassium currents, matured action potential kinetics, and known AP phase plane features), and fluorescent dye in the internal solution to show morphology, we are confident we are recording from neurons, of which >90% are GABAergic.
-Last, the Piggy-Bac transposon based delivery system for ASCL1/DLX provides robust iGABA cultures compared to previous generation viral methods.

8) Page 8, line 3. Figure S2C should be S2D.
Corrected. Thank you

9) Page 19, second line- Figure 5B should be 5C.

Fixed. Thank you

10) Page 19. Improved rundown presumably means less rundown rather than more rundown? This should be clarified and/or a figure added to show what is meant.
We clarify with added text to the results, as well as more definition in methods for rundown.

11) The section about poison exons (p.15) is confusing because it is placed in the section on cell-type expression during development. It either needs a better opening rationale or to be transferred to a different section or to the discussion.

We added an opening rationale to clarify why poison-exon–containing KCNT1 isoforms were analyzed in this developmental-expression section: to test whether these isoforms appear to undergo substantial degradation (e.g., via NMD) during development and to evaluate whether they could plausibly represent future ASO/splicing targets. Our data indicate poison-exon–containing transcripts are detectable and not appreciably depleted, suggesting these exons are unlikely to be strong NMD substrates and may be less compelling therapeutic targets.

12) The sentence beginning " Analysis of the neocortex …" in the same section (p.15) has no verb.
We fixed this. Thank you

13) The sentence in the discussion beginning "Follow-up studies of non-conducting…" (p.22) has no verb.
Fixed, thank you.

14) Last line of first paragraph in introduction (p.6); onset of encephalopathy.
Corrected to 'onset of encephalopathy.' Thank you

Reviewer #3
Thank you for your careful review and comments to improve the manuscript

Reviewer #4
Thank you for your careful review and comments to improve the manuscript

Reviewer #5
Thank you for your careful review and comments to improve the manuscript

Reviewer #6
Thank you for your careful review and comments to improve the manuscript.
